# Reductive Coupling of a Diazoalkane Derivative Promoted by a Potassium Aluminyl and Elimination of Dinitrogen to Generate a Reactive Aluminium Ketimide

**DOI:** 10.1002/chem.202302903

**Published:** 2023-11-07

**Authors:** Matthew J. Evans, Mathew D. Anker, Claire L. McMullin, Martyn P. Coles

**Affiliations:** ^1^ School of Chemical and Physical Sciences Victoria University of Wellington P.O. Box 600 Wellington 6012 New Zealand; ^2^ Department of Chemistry University of Bath Bath BA2 7AY UK

**Keywords:** aluminyl, diazomethane, guanidinate, ketimide, reductive coupling

## Abstract

The reaction of 9‐diazo‐9*H*‐fluorene (fluN_2_) with the potassium aluminyl K[Al(NON)] ([NON]^2−^=[O(SiMe_2_NDipp)_2_]^2−^, Dipp=2,6‐*i*Pr_2_C_6_H_3_) affords K[Al(NON)(κ*N* ^1^,*N* ^3^‐{(fluN_2_)_2_})] (**1**). Structural analysis shows a near planar 1,4‐di(9*H*‐fluoren‐9‐ylidene)tetraazadiide ligand that chelates to the aluminium. The thermally induced elimination of dinitrogen from **1** affords the neutral aluminium ketimide complex, Al(NON)(N=flu)(THF) (**2**) and the 1,2‐di(9*H*‐fluoren‐9‐yl)diazene dianion as the potassium salt, [K_2_(THF)_3_][fluN=Nflu] (**3**). The reaction of **2** with *N*,*N’*‐di*iso*propylcarbodiimide (*i*PrN=C=N*i*Pr) affords the aluminium guanidinate complex, Al(NON){N(*i*Pr)C(N=CMe_2_)N(CHflu)} (**4**), showing a rare example of reactivity at a metal ketimide ligand. Density functional theory (DFT) calculations have been used to examine the bonding in the newly formed [(fluN_2_)_2_]^2−^ ligand in **1** and the ketimide bonding in **2**. The mechanism leading to the formation of **4** has also been studied using this technique.

## Graphical abstract

The potassium aluminyl K[Al(NON)] reductively couples 9‐diazo‐9*H*‐fluorene (fluN_2_) to generate a highly coloured species containing the [fluNNNNflu]^2−^ ligand. Thermal elimination of N_2_ and loss of doubly reduced 1,2‐di(9*H*‐fluoren‐9‐yl)diazene [fluN=Nflu]^2−^ as the potassium salt generates a neutral aluminium ketimide, which undergoes cycloaddition and hydrogen transfer to form the corresponding guanidinate complex.

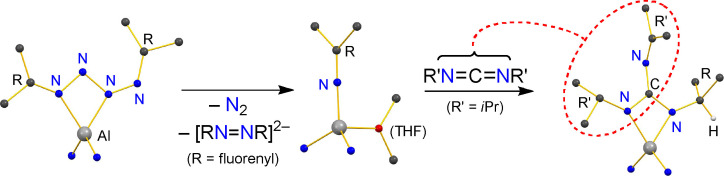

## Introduction

In recent years the application of low valent main group complexes for the activation of small molecules has emerged as a fundamental area of scientific research.^[1]^ In many regards, the reactivity of these compounds may be considered as being equivalent to (or in some cases surpassing) that of the more traditionally studied transition metal complexes.^[2]^ Aluminyl systems, comprised of a negatively charged Al(I) component charge balanced by a group 1 metal cation, are a family of highly reactive main‐group compounds that have recently been established in this respect.^[3]^ Our contribution to this area has focussed on the M[Al(NON)] system ([NON]^2−^=[O(SiMe_2_NDipp)_2_]^2−^, M = Li, Na, K, Rb, Cs),^[4]^ with most studies to date conducted with the potassium variant, K[Al(NON)].

**Figure 1:**
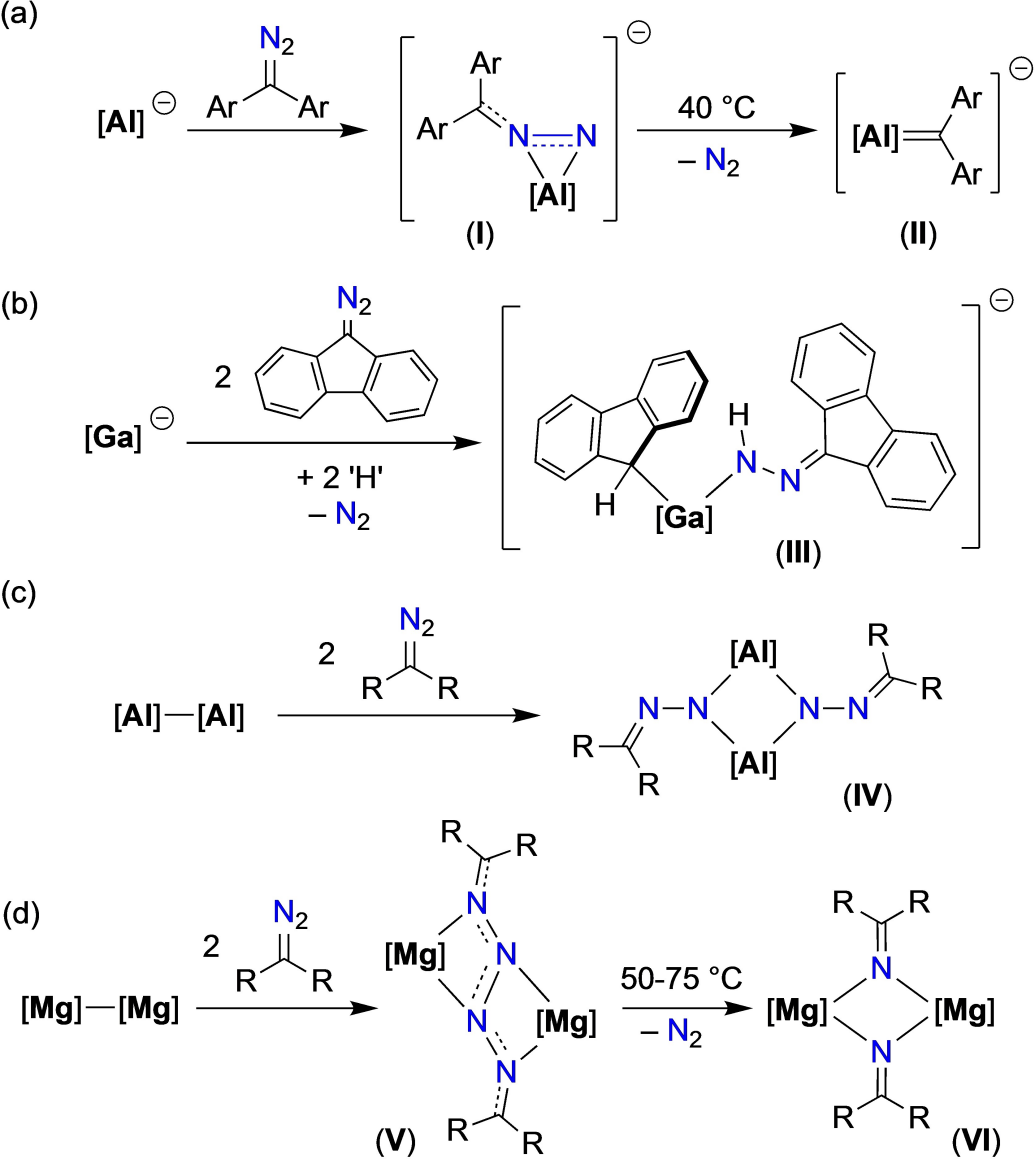
Reactivity of main group compounds with diazomethane derivatives. (a) [**Al**]=[Al(bo2e‐C^Ar^N^Dipp^)]^−^; (b) [**Ga**]=[Ga(DAB^Dipp^)]^−^; (c) [**Al**]=[Al(_Me_DAB^Dipp^)], R_2_CN_2_=(Me_3_Si)(H)CN_2_ and [**Al**]=[Al(BIAN^Dipp^)], R_2_CN_2_=Ph_2_CN_2_; (d) [**Mg**]=Mg(BDI^Mes^), R_2_CN_2_=Ph_2_CN_2_ and fluN_2_. See text for definitions of ligands.

The development of aluminyl compounds has facilitated access to several avenues of reactivity that were previously difficult to study for neutral low‐valent aluminium compounds. One such area is the isolation of compounds containing Al=E multiple bonds, which although previously noted for neutral Al(I) systems,^[5]^ were at the time largely derived from unanticipated reactions.^[6]^ Taking the low valent aluminyl anion as a precursor to Al=E bond formation using an oxidative addition approach, our group and others have reported examples of Al=O,^[7]^ Al=S,^[8]^ Al=Se,^[9]^ Al=Te,^[10]^ and Al=NR^[11]^ bonds. In this context, Kinjo and co‐workers examined the reaction of the lithium salt of a cyclic (alkyl)(amino)aluminyl anion, Li[Al(bo2e‐C^Ar^N^Dipp^)] ([bo2e‐C^Ar^N^Dipp^]^2−^=[(bicyclo[2.2.2]oct‐2‐ene)C(Ar)_2_N(Dipp)]^2−^, Ar=3,5‐*t*Bu_2_C_6_H_3_) with the bulky diaryldiazoalkane, Ar_2_CN_2_ as a route to Al=CR_2_ systems (Figure 1a).^[12]^ The initial product of the reaction (**I**) contained a three‐membered diazo‐aluminium ring. Under mild thermal conditions this compound underwent the anticipated thermal elimination of N_2_ to afford a product containing a short Al−C bond (**II**), shown by DFT analysis to contain an exocyclic Al=C group with significant π‐bonding character. Intrigued by this reaction, we wished to examine the reactivity of our K[Al(NON)] system towards diazoalkanes.

Previous work studying the reaction of main group systems with diazoalkanes showed several possible outcomes for the reaction, in addition to that affording **II**. For example, the reaction of a potassium gallyl with two equivalents of 9‐diazo‐9*H*‐fluorene (fluN_2_) in the presence of 18‐crown‐6 (18‐c‐6) proceeds in an ill‐defined reaction to afford a low yield of [K(18‐c‐6)][Ga(DAB^Dipp^){N(H)N=flu}] (DAB^Dipp^=[C(H)NDipp]_2_) (**III**, Figure 1b).^[13]^ The crystallographically characterized product showed loss of N_2_ from one equivalent of fluN_2_ and incorporation of ‘H’ at both the *C*‐9 position of a flu‐substituent and within a [N(H)N=flu]^−^ ligand, believed to originate from the reaction solvent. In contrast, the reaction of redox active Al(II) species [Al(_Me_DAB^Dipp^)]_2_ and [Al(BIAN^Dipp^)]_2_ (_Me_DAB^Dipp^=[C(Me)NDipp]_2_; BIAN^Dipp^=1,2‐(Dipp‐imino)_2_‐acenaphthene) with trimethylsilyldiazomethane and diphenyldiazomethane, respectively, afforded bimetallic complexes in which the terminal nitrogen of the diazoalkane has inserted into the Al−Al bond to afford a Al_2_N_2_ core (**IV**, Figure 1c).^[14]^ Crystallographic analysis of bond lengths confirmed N−N single bonds and C=N double bonds within the [R_2_C=N‐N]^2−^ groups, confirming reduction of the diazoalkane and formation of a *μ*‐imide ligand. More recently, Stephan and co‐workers demonstrated the reductive coupling of two equivalents of Ph_2_CN_2_ using Jones’ Mg(I) reagent, [Mg(BDI^Mes^)]_2_ (**V**, Figure 1d. BDI^Mes^=[HC{C(Me)NMes}_2_]^−^).^[15]^ The resulting [R_2_CNNNNCR_2_]^2−^ ligands bridge two Mg centres, with delocalisation focussed in the terminal NNCR_2_ units. Thermal decomposition of these compounds proceeded with loss of N_2_ to afford the corresponding *μ*‐ketimide compounds containing a central Mg_2_N_2_ core (**VI**).

To complement our previous studies between organic azides and low‐valent (indyl/aluminyl)[^11a^, ^16^] or bimetallic (In−Zn)^[17]^ systems, we herein report the reductive coupling of fluN_2_ by the potassium aluminyl K[Al(NON)], a thermally induced extrusion of N_2_ to afford a terminal ketimide ligand, and the reactivity of this complex with a carbodiimide. These results are supported by DFT calculations examining the bonding within the new complexes, and the mechanism of the reaction of the ketimide complex with carbodiimide.

## Results and Discussion

### Reductive coupling of 9‐diazo‐9H‐fluorene (fluN_2_) by the potassium aluminyl K[Al(NON)]

**Scheme 1:**
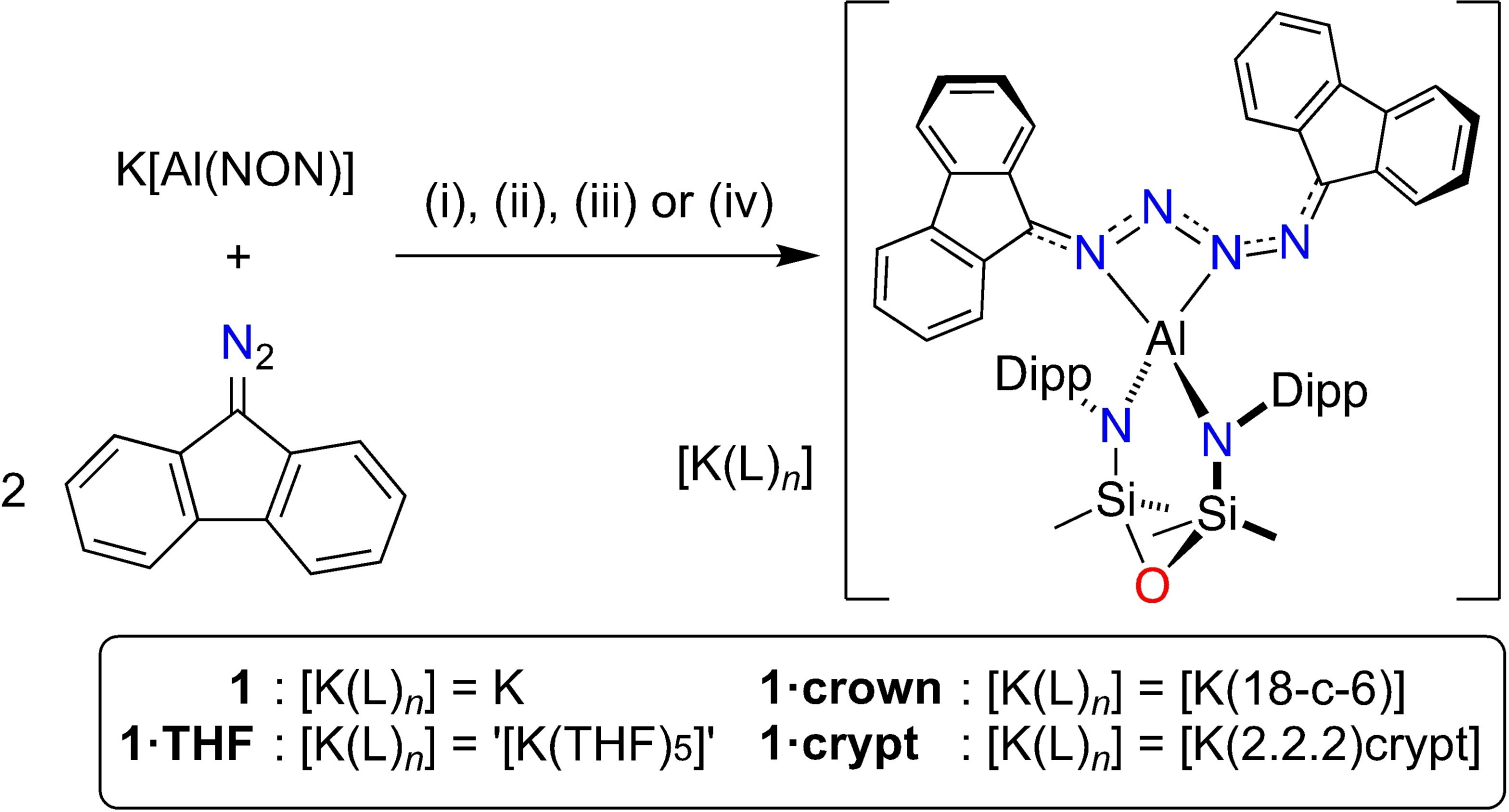
Synthesis of **1**. (i) Toluene (**1**); (ii) Toluene/THF (**1⋅THF**); (iii) 18‐crown‐6, hexane/THF (**1⋅crown**); (iv) (2.2.2)cryptand, hexane/THF (**1⋅crypt**).

The addition of 1 equivalent of 9‐diazo‐9*H*‐fluorene (fluN_2_) to a solution of K[Al(NON)] in toluene initially afforded an intense blue solution that turned dark green upon standing at room temperature. Filtration and slow evaporation of the solvent yielded dark green crystals **1**, shown by X‐ray diffraction to be K[Al(NON){(fluN_2_)_2_}] (Scheme 1). Attempted isolation of the blue intermediate was unsuccessful, and repeating the reaction with two equivalents of fluN_2_ gave a higher yield of **1**. We postulate that the blue intermediate is analogous to complex **I**, but that this initially formed diazo‐aluminium ring reacts rapidly with a second equivalent of fluN_2_ to afford **1**. Compound **1** could also be isolated as the THF solvate [K(THF)_5_][Al(NON){(fluN_2_)_2_}] (**1⋅THF**) when crystallized from a toluene/THF mixture (~10 : 1) at −30 °C. Furthermore, to investigate the role that cation:anion interactions have in the structure and stability of **1**, we isolated the [Al(NON){(fluN_2_)_2_}]^−^ anion as the [K(18‐c‐6)]^+^ (18‐c‐6=18‐crown‐6) and [K([2.2.2]crypt)]^+^ ([2.2.2]crypt=[2.2.2]cryptand species, **1⋅crown** and **1⋅crypt**, respectively.

The ^1^H NMR spectrum of **1** shows broad peaks between *δ*_H_ 9.01‐6.40 corresponding to overlapping signals for the [(fluN_2_)_2_]^2−^ ligand and Dipp substituents. In contrast, the ^1^H NMR spectra of **1⋅THF** and **1⋅crown** show sharp resonances for the flu‐ and Dipp‐substituents, with multiple overlapping signals in the aromatic region (**1⋅THF** ×10 signals; **1⋅crown** ×15 signals). These data are consistent with a high degree of asymmetry in the [Al(NON){(fluN_2_)_2_}]^−^ anion.

**Figure 2:**
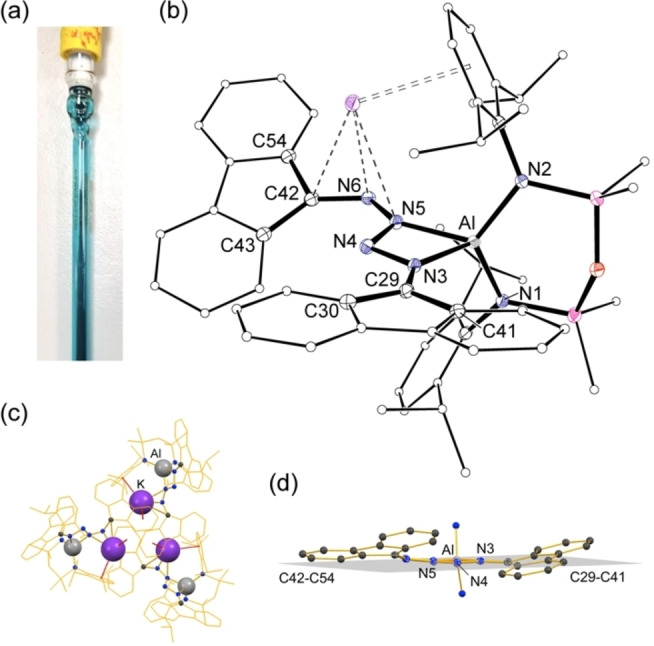
(a) Photograph of an NMR solution of **1⋅THF** in C_6_D_6_. (b) Displacement ellipsoid plots of **1** (30 % ellipsoids; H‐atoms omitted; C‐atoms except at key positions represented as spheres). (c) View along the *b*‐axis of **1**. (d) View in the AlN_3_‐plane of the anion from the crystal structure of **1⋅crypt**.

The intense colour of the compounds in solution (Figure 2a) prompted analysis by UV/vis spectroscopy. Solutions of **1** and **1⋅THF** showed strong absorptions at λ_max_ 657 nm (**1**, ϵ=16138 L mol^−1^ cm^−1^; **1⋅THF**, ϵ=22321 L mol^−1^ cm^−1^), with a second strong absorption band at 381 nm (ϵ=14108 L mol^−1^ cm^−1^) noted for **1⋅THF** (Figures S2, S6, S11 in Supporting Information). In contrast, the major absorption observed in a solution of **1⋅crown** was slightly red‐shifted, with strong bands at 688 nm (ϵ=26642 L mol^−1^ cm^−1^) and 667 nm (ϵ=25913 L mol^−1^ cm^−1^). These data are similar to those obtained for a bimetallic iron product that was generated from the reductive coupling of diazoesters, which form a “burgundy” solution in hexane (λ_max_ 516 nm, ϵ range: 16403 L mol^−1^ cm^−1^ to 22727 L mol^−1^ cm^−1^).^[18]^ The observations suggest that the origin of the intense colour derives from transitions within the [Al(NON){(fluN_2_)_2_}]^−^ anions of **1**, **1⋅THF** and **1⋅crown**, and is largely invariant of the solvation of the cation and any anion⋅⋅⋅cation interactions.

The crystal structure of **1** confirms formation of K[Al(NON)(κ‐*N* ^1^,*N* ^3^‐{(fluN_2_)_2_})], in which reductive coupling of two equivalents of fluN_2_ has occurred (Figure 2b). The potassium atom has short contacts with a ‘CN_2_’ portion of the newly formed 1,4‐di(9*H*‐fluoren‐9‐ylidene)tetraazadiide ligand, with π‐arene interactions to a Dipp substituent. Additional interactions with a fluorene ring of an adjacent molecule generates a 3‐fold helical chain aligned parallel to the *b*‐axis (Figure 2c). The THF‐solvated form **1⋅THF** crystallizes as the unusual potassium‐potassiate [K(THF)_6_][{Al(NON)({(fluN_2_)_2_})}_2_{*μ*‐K(THF)_4_}]. The anion consists of two Al‐centred anions that are bridged by a [K(THF)_4_] cation via C⋅⋅⋅K contacts with a flu‐group (Figure S8). A separated [K(THF)_6_]^+^ cation is present to balance the charge. Although X‐ray quality crystals were not obtained for **1⋅crown**, the [2.2.2]cryptand derivative **1⋅crypt** afforded the expected separated ion pair, [K([2.2.2]crypt)][Al(NON){(fluN_2_)_2_}] (Figure S12).

**Figure 3:**
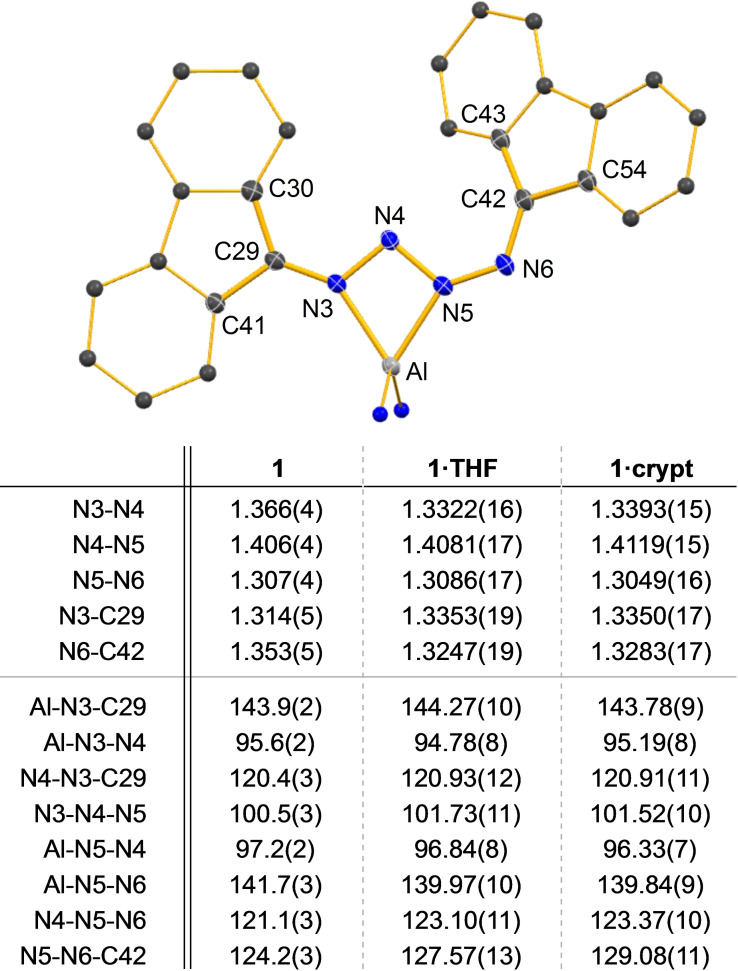
Summary of bond lengths (Å) and angles (°) for the [(fluN_2_)_2_]^2−^ ligand in **1**, **1⋅THF** and **1⋅crypt**.

**Scheme 2:**
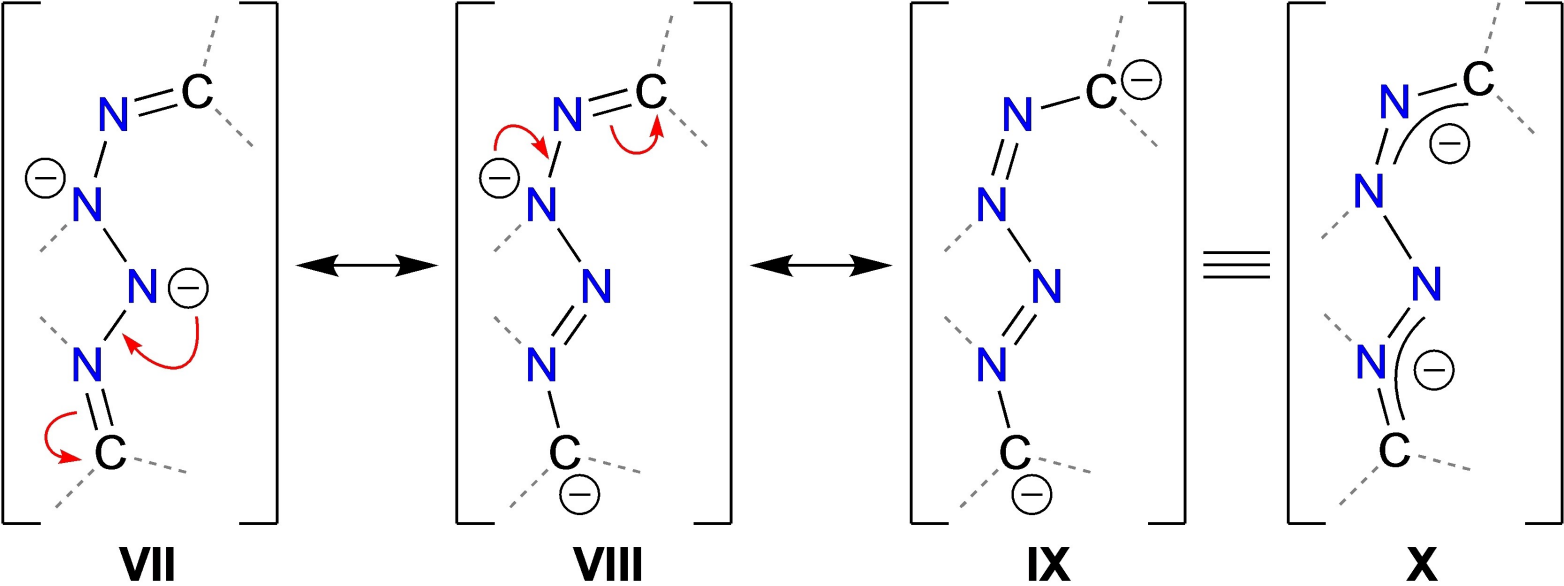
Resonance structures (**VII–IX**) for the [(fluN_2_)_2_]^2−^ dianion with the delocalized hybrid **X** (flu‐groups not shown).

In all cases, the [(fluN_2_)_2_]^2−^ ligand adopts a κ‐*N* ^1^,*N* ^3^‐coordination mode to generate a four‐membered AlN_3_‐ metallacycle with an acute (~67°) bite angle (Table 1). The N−N bond lengths within the N_4_‐component of the ring (range: 1.3049(16) Å to 1.4119(15) Å) fall within the range expected for N−N single (~1.45 Å) and N=N double (~1.24 Å) bonds (Figure 3).^[19]^ In addition, the C−N bonds to the flu‐groups (range: 1.314(5) Å to 1.353(5) Å) are within the limits expected for N−C single (~1.47 Å) and N=C double (~1.28 Å) bonds.^[19]^ These differ significantly from the crystallographically determined values for the parent fluN_2_ molecule (range N−N: 1.124(4) Å to 1.134(3) Å; range C−N: 1.317(3) Å to 1.326(4) Å),^[20]^ suggesting delocalisation within the bonding of the [(fluN_2_)_2_]^2−^ ligand. This is also inferred from the orientation of the flu‐groups relative to the AlN_3_ plane (Figure 2d, Table 1), where in the absence of any potential distortion due to π‐arene interactions with the potassium cation in **1⋅crypt**, the interplanar angles θ1 (C29‐C41 flu‐group) and θ2 (C42‐C54 flu‐group) are 15.6° and 23.4°, respectively. The resonance structures for the [(R_2_CN_2_)_2_]^2−^ group are consistent with these observations (Scheme 2), in which the delocalisation is concentrated in the terminal C−N and N−N bonds, with a longer N4‐N5 bond.

**Table 1:** Selected bond lengths (Å) and angles (°) for **1**, **1⋅THF** and **1⋅crypt**.

	**1**	**1⋅THF**	**1⋅crypt**
Al−N1	1.820(3)	1.8210(12)	1.8311(11)
Al−N2	1.825(3)	1.8274(12)	1.8277(11)
Al−N3	1.973(3)	1.9851(13)	1.9728(11)
Al−N5	1.909(3)	1.8820(12)	1.8924(11)
			
N1‐Al−N2	119.99(14)	114.92(6)	115.94(5)
N3‐Al−N5	66.57(13)	66.64(5)	66.89(5)
θ1^[a]^	8.3	10.9	15.6
θ2^[b]^	30.8	20.5	23.4

[a] Angle between planes defined by Al−N3‐N4‐N5 and C29‐C41 flu‐group. [b] Angle between planes defined by Al−N3‐N4‐N5 and C42‐C54 flu‐group.

We have used density functional theory (DFT) calculations to examine the bonding in the ‘Al{(fluN_2_)_2_}’ group (see Supporting Information for full details). The structure of **1** was optimized with (**1_DFT_**) and without (**1’_DFT_**) the potassium present to determine the influence of the cation on the bonding. The results indicated that although the energy of the system (calculated in THF, ΔG_THF_) increased upon loss of the potassium (ΔG_THF_ **1’_DFT_** =+19.6 kcal mol^−1^ relative to **1_DFT_**) the bond parameters are not significantly perturbed (Figure S25); the data for **1’_DFT_** is used in the following discussion.

In agreement with crystal structure data, the Wiberg bond indices (WBIs) confirm a lower bond order between the N4 and N5 atoms (WBI=1.12) when compared to the remaining N−N (WBI=1.34/1.36) and N‐C_flu_ (WBI=1.31/1.42) bonds. This can also be inferred from NBO data in which two bonding orbitals are identified between N3/N4 and N5/N6, but only a single bonding orbital was calculated between N4/N5 (Table S5). Delocalisation from the π‐system of the flu‐substituents is suggested by the large energies calculated for the donation from a lone‐pair of electrons on the carbon at the 9‐position of the flu‐substituent into an adjacent N−N anti‐bonding orbital (652 and 667 kcal mol^−1^ for C29 and C42, respectively).

**Figure 4:**
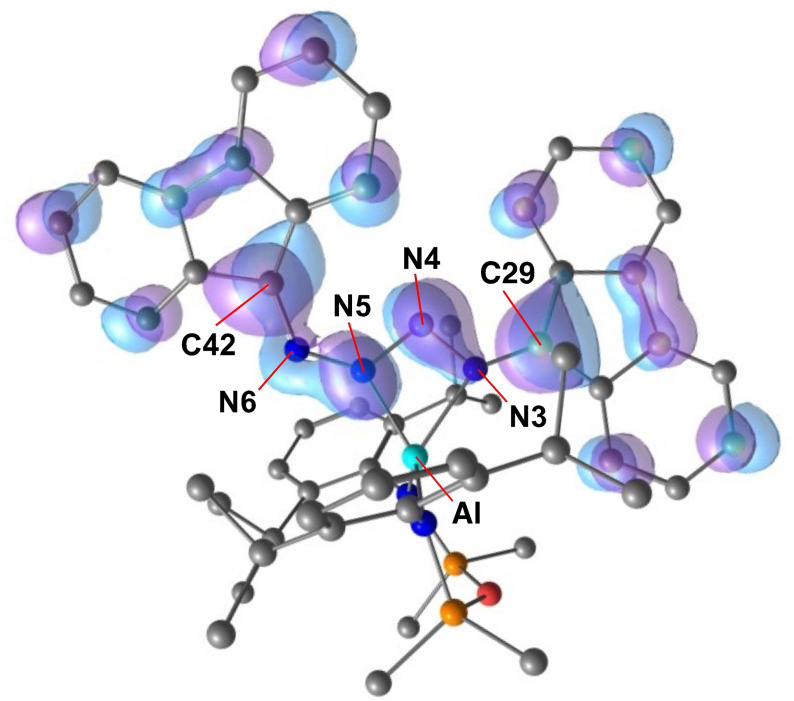
Highest occupied molecular orbital (HOMO) of **1’_DFT_** from NBO7 analysis (BP86/BS2//BP86/BS1).

The highest occupied molecular orbital (HOMO) is composed of π‐symmetry lobes that are distributed throughout the [(fluN_2_)_2_]^2−^ ligand (Figure 4). These show good overlap in the N3‐N4 bond, with an additional component of the orbital that is delocalized across the N5‐N6‐C42 portion. We note that the orbital is π‐anti‐bonding across the N4‐N5 bond, consistent with the proposed single bond.

### Thermal decomposition of K[Al(NON){(fluN_2_)_2_}]

**Scheme 3:**
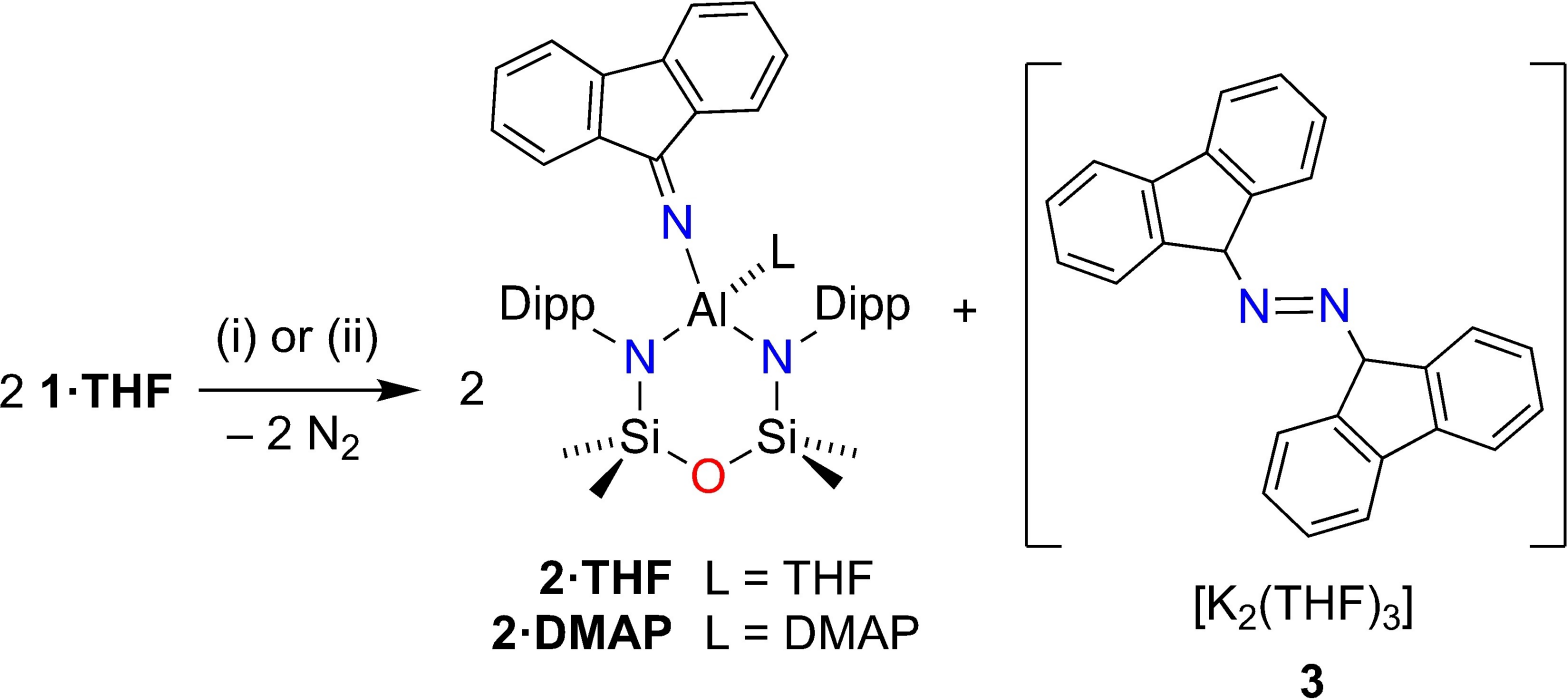
Synthesis of **2** and **3**. (i) 80 °C, Toluene/THF. (ii) 80 °C, DMAP, toluene/THF.

The clean elimination of N_2_ from mono‐ and reductively coupled diazoalkane products **I** and **V** under thermal conditions inspired us to examine the stability of the [Al(NON){(fluN_2_)_2_}]^−^ anion. Thus, a sample of **1⋅THF** was heated to 80 °C in C_6_D_6_ to generate a dark green solution over the course of 18 h (Scheme 3). Following work‐up, a clean sample of Al(NON)(N=flu)(THF) (**2⋅THF**) was isolated via fractional crystallization as intense blue crystals. Alternatively, the corresponding 4‐dimethylaminopyridine (DMAP) derivative (**2⋅DMAP**) was isolated by adding DMAP to an in situ formed solution of **2⋅THF**.

The ^1^H NMR spectrum of **2⋅THF** displays peaks for the NON‐ligand and flu‐groups, in addition to an equivalent of THF. At room temperature the signals are broad, with overlapping C*H*Me_2_ resonances (*δ*_H_ 4.06‐3.73) and singlets at *δ*_H_ 0.43 and 0.40 for the Si*Me_2_* groups. Notably, the low field resonances of the THF molecule appear as two peaks at *δ*_H_ 3.87 (2H) and 3.50 (2H) consistent with inequivalent OC*H_2_* environments. Upon heating to 70 °C, the *i*Pr methine and THF signals each coalesce to form a septet at *δ*_H_ 3.90 and broad singlet *δ*_H_ 3.69, respectively (Figure S15). These data indicate a crowded environment at the Al centre with restricted rotation of the THF and N*Dipp* groups at room temperature.

**Figure 5:**
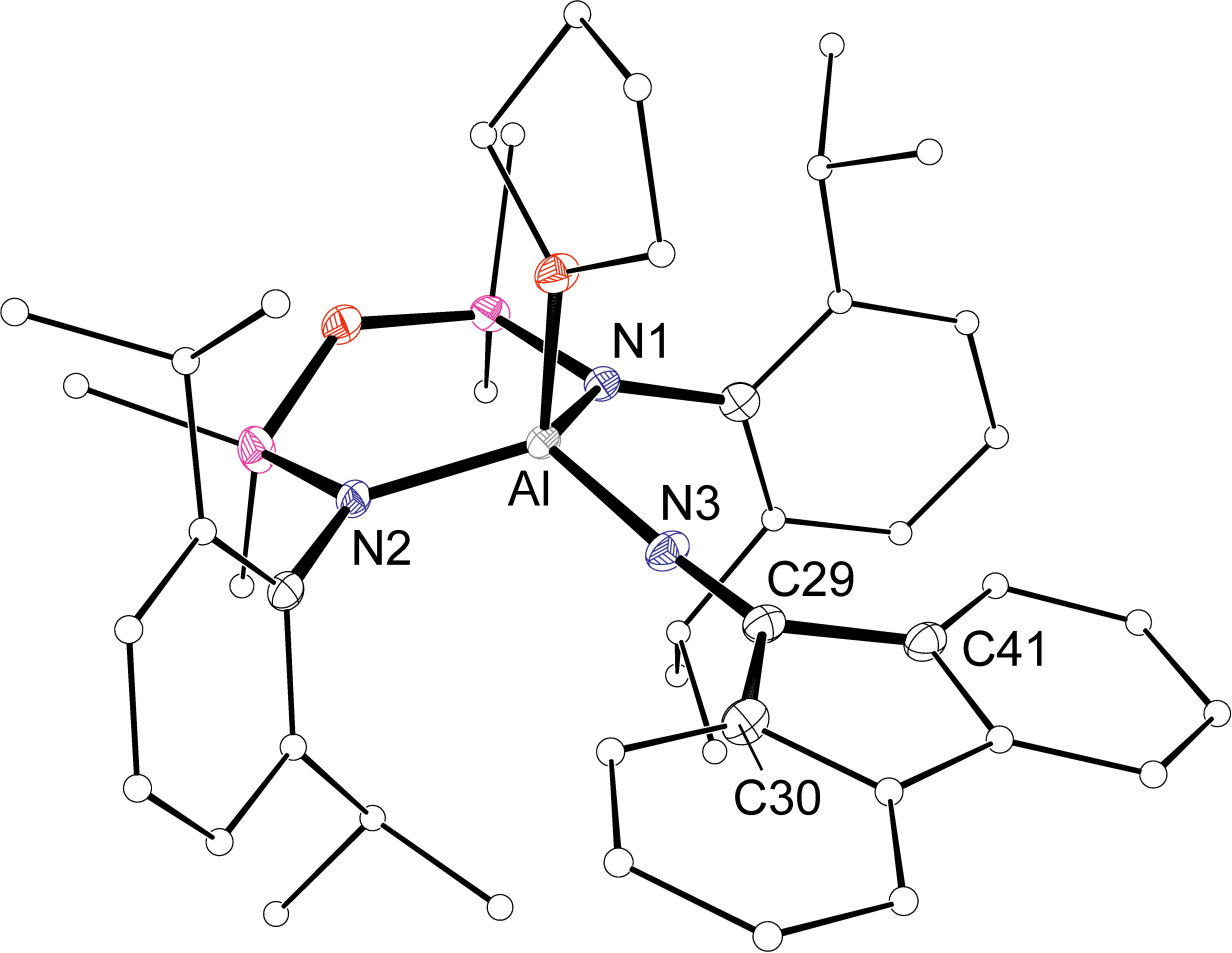
Displacement ellipsoid plots of **2⋅THF** (30 % ellipsoids; H‐atoms omitted; C‐atoms except at key positions represented as spheres).

The molecular structures of **2⋅THF** (Figure 5) and **2⋅DMAP** (Figure S20) were verified crystallographically. Depending on the conditions of crystallization, **2⋅THF** was isolated with either an included THF (**2⋅THF{THF}**, monoclinic, *P*2_1_) or included benzene and toluene (**2⋅THF{Ar}**, triclinic, P$\bar{1}$ ); bond data for both variants are included in Table 2 for comparison. In all cases the product exists as the neutral Al(NON)(N=flu)(L) complex (L = THF or DMAP).

**Table 2:** Selected bond lengths (Å) and angles (°) for **2⋅THF{THF}**, **2⋅THF{Ar}** and **2⋅DMAP**.

	**2⋅THF{THF}**	**2⋅THF{Ar}**	**2⋅DMAP**
Al−N1	1.863(4)	1.8486(15)	1.8609(12)
Al−N2	1.855(4)	1.8467(15)	1.8610(13)
Al−N3	1.774(4)	1.7779(16)	1.7850(13)
N3‐C29	1.255(5)	1.253(2)	1.256(2)
			
N1‐Al−N2	113.68(15)	115.66(7)	108.96(6)
N1‐Al−N3	116.4(2)	116.21(7)	118.82(6)
N2‐Al−N3	116.15(19)	115.14(7)	115.21(6)
Al−N3‐C29	176.3(4)	178.12(15)	170.88(12)
N3‐C29‐C30	127.5(4)	127.77(17)	127.99(15)
N3‐C29‐C41	128.5(4)	128.24(18)	128.15(14)
C30‐C29‐C41	104.1(4)	103.99(15)	103.81(12)

The four‐coordinate aluminium centre adopts a distorted tetrahedral geometry supported by an *N*’*N’*‐chelated NON‐ligand, a terminal [N=flu]^−^ ketimide group and a neutral L‐donor ligand. The Al−N3 bonds to the [N=flu]^−^ ligand are in the range 1.774(4) Å to 1.7850(13) Å, which are comparable with other terminal Al‐N=CR_2_ ligands in Li[Al(N=C*t*Bu_2_)_4_] (1.78(1) Å),^[21]^ [Al(N=CPh_2_)_2_(*μ*‐N=CPh_2_)]_2_ (range: 1.774(3) Å to 1.796(3) Å),^[22]^ Li[Al(bo2e‐C^Ar^N^Dipp^)(N=CAr_2_)_2_], (1.7942(16) Å),^[12]^ and Al(BDI)(N=CPh_2_)_2_ (1.775(4) Å and 1.784(5) Å).^[23]^ We note that these Al−N bond lengths in **2** are intermediate between the observed in the three‐ coordinate aluminium imide K[Al(NON)(NMes)] (1.7251(11) Å),^[11a]^ and the tetrahedral (amido)(hydrido)aluminate, [Al(NON)(H){N(H)Dipp}]^−^ (1.8579(13) Å).^[24]^ As expected, the N3−C29 distances (range: 1.253(2) Å to 1.256(2) Å) are consistent with N=C double bonds. However, we note that the Al−N3‐C29 angles in all structurally characterized variants of **2** (range: 170.88(12)° to 178.12(15)°; ave. 175.1°) are generally closer to linear than found in other examples (range: 148.1(2)° to 175.1(2)°; ave. 160.9°), a likely consequence of the size and rigidity of the flu substituent.

DFT calculations on transition metal ketimide complexes have previously concluded that the [N=CR_2_]^−^ ligand exhibits strong π‐donor and π‐acceptor properties involving the nitrogen lone‐pair and the orthogonal N=C anti‐bonding orbital, respectively.^[25]^ For comparison, DFT calculations were performed on Al(NON)(N=flu)(THF) (**2⋅THF_DFT_**) and the THF‐free unit, Al(NON)(N=flu) (**2_DFT_**). The inclusion of THF in the structure stabilized the Al(NON)(N=flu) group by 7.0 kcal mol^−1^, but otherwise did not significantly change the bond parameters; the data for **2_DFT_** is included in the following discussion.

**Figure 6:**
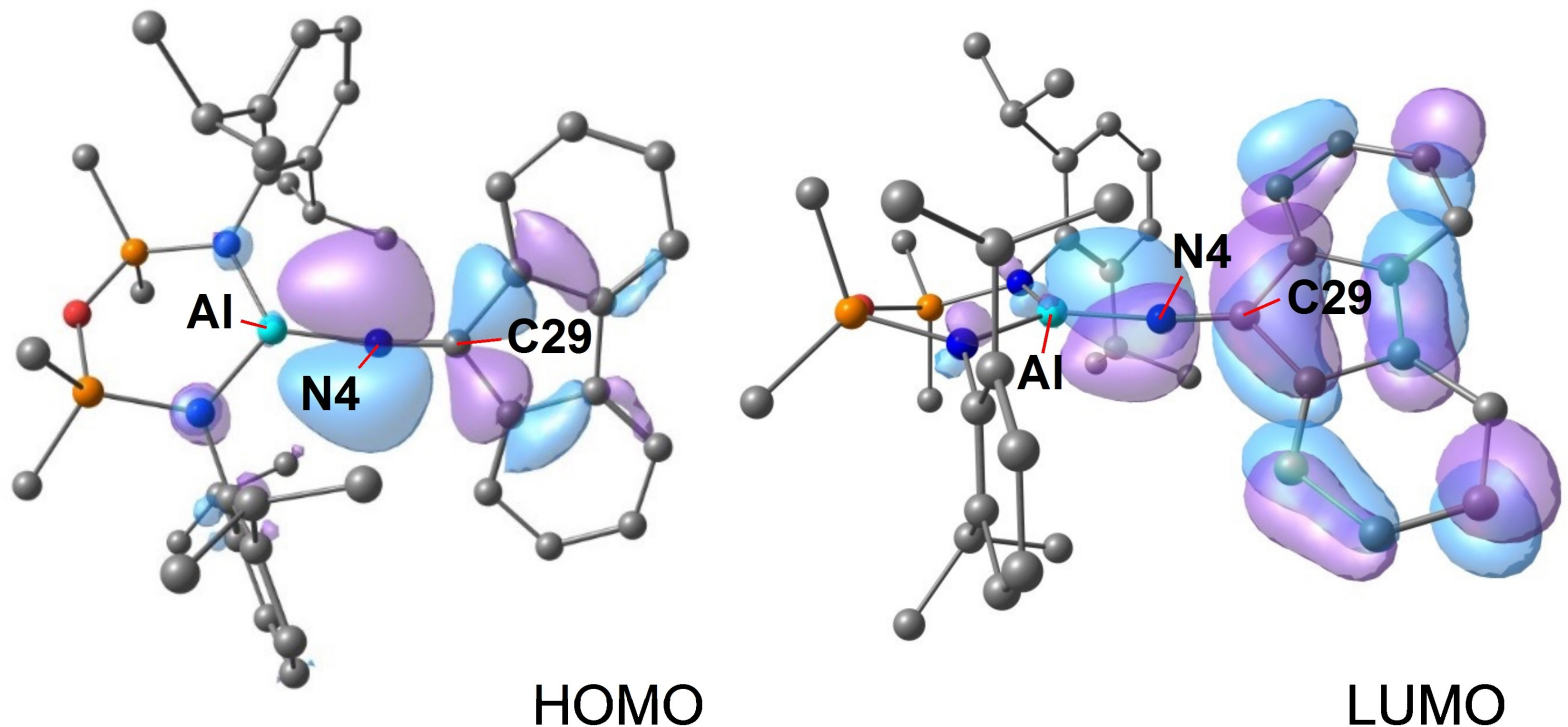
Highest occupied molecular orbital (HOMO) and lowest unoccupied molecular orbital (LUMO) of **2_DFT_** from NBO7 analysis (BP86/BS2//BP86/BS1).

The WBIs for the ‘Al(N=flu)’ group confirm a high bond order between the nitrogen and the carbon of the flu‐substituent (WBI=1.79) and a relatively weak Al−N bond (WBI=0.42) (Figure S26). The HOMO is largely composed of an anti‐bonding π* interaction between the nitrogen and carbon of the ketimide group, with lobes that extend across the Al−N bond (Figure 6). The LUMO has similar π* anti‐bonding character that is orthogonal to the HOMO. This orientation allows a better overlap with the π‐system of the flu‐ligand. NBO analysis confirms two bonding interactions between the nitrogen and carbon of the N=flu ligand, with no NBOs detected between the Al and N4. There are however some donor‐acceptor interactions between the lone‐pair of the nitrogen and an unoccupied orbital on aluminium, with energies of 51.8 and 42.4 kcal mol^−1^.

**Figure 7:**
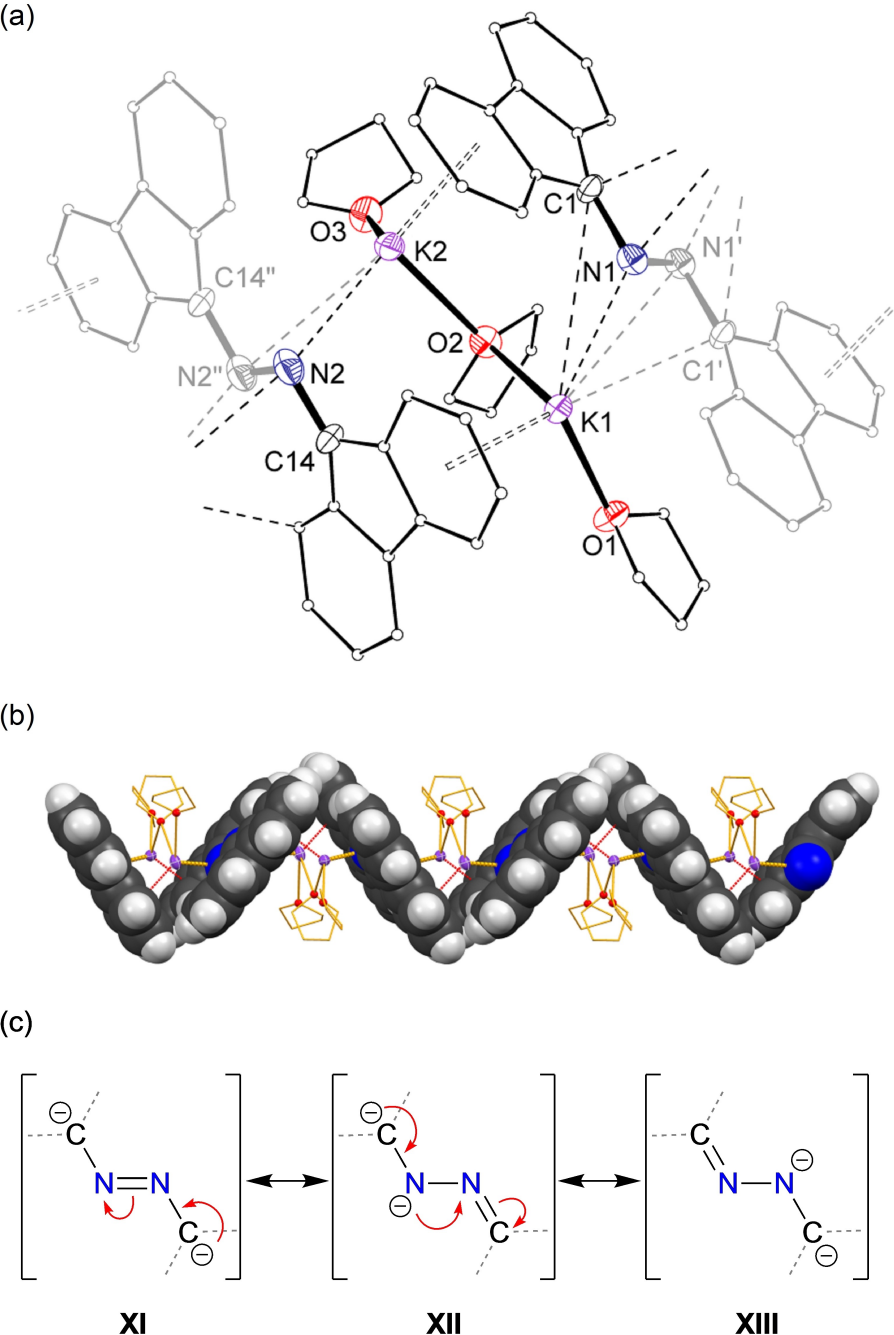
(a) Displacement ellipsoid plots of **3** (20 % ellipsoids; H‐atoms and disordered components omitted; C‐atoms except at key positions represented as spheres; symmetry generated atoms greyscale. ‘=‐*x*, 1‐*y*, ‐*z*; “=1‐*x*, 1‐*y*, ‐*z*). (b) View perpendicular to chain showing zig‐zag arrangement of planar [NFlu]_2_^2−^ units bridged by [K_2_(THF)_3_]^2+^ dications. Selected bond lengths (Å): N1‐N1’ 1.227(17), N2‐N2’ 1.240(9), N1‐C1 1.532(13), N2‐C14 1.456(7). (c) Resonance structures (**XI–XIII**) for the [(fluN)_2_]^2−^ dianion (flu‐groups not shown).

The formation of neutral products **2** is unusual in aluminyl chemistry, and the fate of the potassium was initially unknown. However, we were able to separate a small number of dark blue crystals **3** during the isolation of **2⋅DMAP** that offer insight into the fate of the potassium and allow a balanced equation to be proposed (Scheme 3). Unfortunately, the low yield and high sensitivity prevented the isolation of pure samples of **3** for spectroscopic analysis. However, the X‐ray diffraction confirmed the composition of **3** to be the 1,2‐di(9*H*‐fluoren‐9‐yl)diazene dianion, that was isolated as the THF solvated form, [K_2_(THF)_3_][(fluN)_2_] (Figure 7a).

The asymmetric unit consists of two ‘fluN’ half‐molecules that are each located across an inversion centre that generates the corresponding symmetry related components. The resulting [(fluN)_2_]^2−^ units are approximately planar and are present in a polymeric zig‐zag arrangement with an internal angle of ~70° between adjacent planes. The cationic [K_2_(THF)_3_]^2+^ group, in which each potassium is bonded to a terminal THF with a bridging THF between the cations, is located between adjacent anions and is supported by K⋅⋅⋅N and K⋅⋅⋅π(arene) interactions (Figure 7b).

Although considerable disorder is noted in the structure, the N−N distances of 1.227(17) Å and 1.240(9) Å indicate N=N double bonds (C_Ar_‐N=N‐C_Ar_=1.255 Å^[19]^) and are consistent with a major contribution from resonance structure **XI** (Figure 7c). Additional support is presented by the N‐C_flu_ bond lengths of 1.532(13) Å and 1.456(7) Å, which are closer to single bond distances. These parameters differ significantly from those of the neutral azine flu=N−N=flu (N−N 1.361(6) Å/1.384(2) Å; C=N 1.335(5) Å/1.299(2) Å),[^15^, ^26^] indicating the dianion is best described as an (*E*)‐9,9’‐(diazene‐1,2‐diyl)bis(9*H*‐fluoren‐9‐ide) group, with the negative charge likely delocalized within the flu‐substituent.

Formation of 2,3‐diaza‐1,3‐butadienes (azines, R_2_C=N‐N=CR_2_) from diazomethane complexes is a known organic transformation,^[27]^ and research into such compounds are of continued interest due to their interesting chemical, biological and materials properties.^[28]^ Furthermore, [(fluN)_2_]^2−^ has been generated electrochemically, but is readily protonated by solvent to afford [flu=NN(H)flu]^−^ and flu(H)N(H)N=flu.^[29]^ To the best of our knowledge **3** represents the first time that a doubly reduced azine has been isolated and structurally characterized.

### Reaction of Al(NON)(N=flu)(THF) with iPrN=C=NiPr

Ketimides [N=CR_2_]^−^ are generally considered as unreactive ligands and examples of their involvement in reactions are scarce. As such they have been employed to support a number of reactive metal centres,^[30]^ and are often encountered as spectator ligands in catalytic reactions.^[31]^ A notable exception demonstrating reactivity of a thorium‐ketimide bond was reported in 2014 by Liddle and co‐workers.^[32]^ In this instance, insertion of the carbonyl bond of *tert*‐butylisocyanate or 9‐anthracene carboxaldehyde into the Th‐N_*ketimide*_ was observed, affording the ureate and alkoxide complexes, respectively. To probe the reactivity of the ketimide bond of **2**, we performed the reaction of an in situ generated solution with di*iso*propylcarbodiimide, *i*PrN=C=N*i*Pr.

**Scheme 4:**
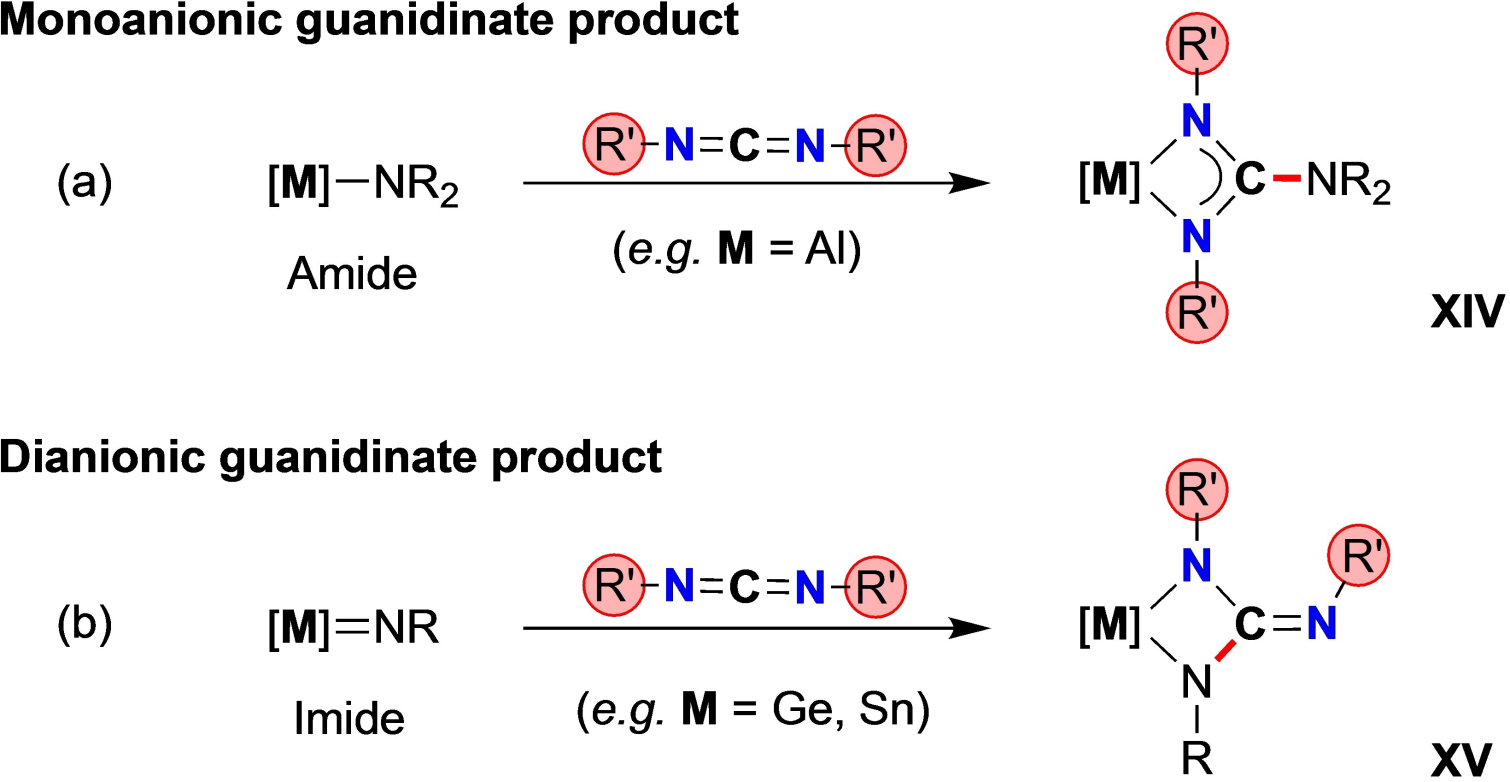
General pathways observed for the reaction of M‐NR_2_ amide (insertion) and M=NR imide (cycloaddition) bonds with carbodiimides.

Based on the contrasting reactivity of metal‐amide and ‐imide bonds, two available reaction pathways have been identified. The insertion of a carbodiimide R'N=C=NR’ into an Al‐NR_2_ (amido) bond is a known route to monoanionic guanidinate complexes Al({NR’}_2_C‐NR_2_)X_2_ (**XIV**, Scheme 4a).^[33]^ The mechanism has been studied computationally and shown to proceed in three steps: (i) coordination of a carbodiimide *N*‐atom to the Al centre; (ii) migration of the terminal amido group to the *sp*‐hybridized *C*‐atom; (iii) coordination of the remaining carbodiimide nitrogen to the metal.^[34]^ In contrast, metal imides typically react with carbodiimides via a cycloaddition reaction with the terminal M=NR bond to form a dianionic guanidinate (**XV**, Scheme 4b). Although isolable aluminium imides represent a relatively new area of chemistry and their reaction with carbodiimides has not been reported, reactions with related unsaturated substrates (e. g. CO_2_, RCNO) have been shown to proceed along this cycloaddition pathway.[^11a^, ^11c^] Correspondingly, the reaction of germanimine and stannaimine complexes (containing the Ge=NR and Sn=NR groups, respectively) with carbodiimides affords dianionic guanidinate products.^[35]^ The distribution of substituents in the resulting MN_2_C‐metallacycle of the products may give an indication of the dominant mechanism. Thus, amides react to afford the product in which the carbodiimide N*R’* substituents are in a 1,3‐pattern (**XIV**), whereas the expected product from cycloaddition places them in adjacent 1,2‐positions (**XV**). We note that the formation of dianionic guanidinates **XV** in the reaction between **2** and a carbodiimide would necessitate additional reactivity to balance electronic charge at the metal.

**Scheme 5:**
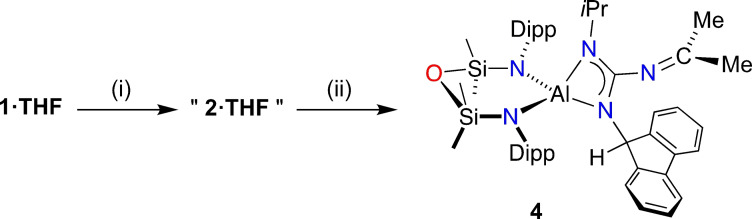
Synthesis of **4**. (i) 80 °C, C_6_D_6_, 18 h; (ii) *i*PrN=C=N*i*Pr, 15 min.

An in situ generated solution of **2⋅THF** (obtained by heating a solution of **1⋅THF** to 80 °C in benzene for 18 h) was reacted with di*iso*propylcarbodiimide to afford dark green crystals **4** (Scheme 5). The ^13^C{^1^H} NMR spectrum of **4** showed a low field resonance at *δ*_C_ 170.3, which is in the region expected for aluminium guanidinate complexes **XIV** or **XV** (*δ*_C_ range: ~162–173),[^33^, ^36^] and is consistent with reaction at the ketimide ligand. The ^1^H NMR spectrum showed the characteristic pattern for a chelating NON‐ligand at an aluminium with local *C*_1_ symmetry (i. e. tetrahedral Al(NON)(X)(Y), where X ≠ Y), inconsistent with the formation of a symmetrical guanidinate, **XIV**. Furthermore, a singlet resonance at *δ*_H_ 0.59 (6H) with no corresponding septet that has a corresponding low field peak in the ^13^C{^1^H} NMR spectrum at *δ*_C_ 169.8 indicated conversion of an *i*Pr substituent to an *N*‐(propan‐2‐ylidene) group. Additional resonances at *δ*_H_ 5.07 (1H) and *δ*_C_ 59.5 (*C*H) suggest the H‐atom is located on the flu‐ substituent.

**Figure 8:**
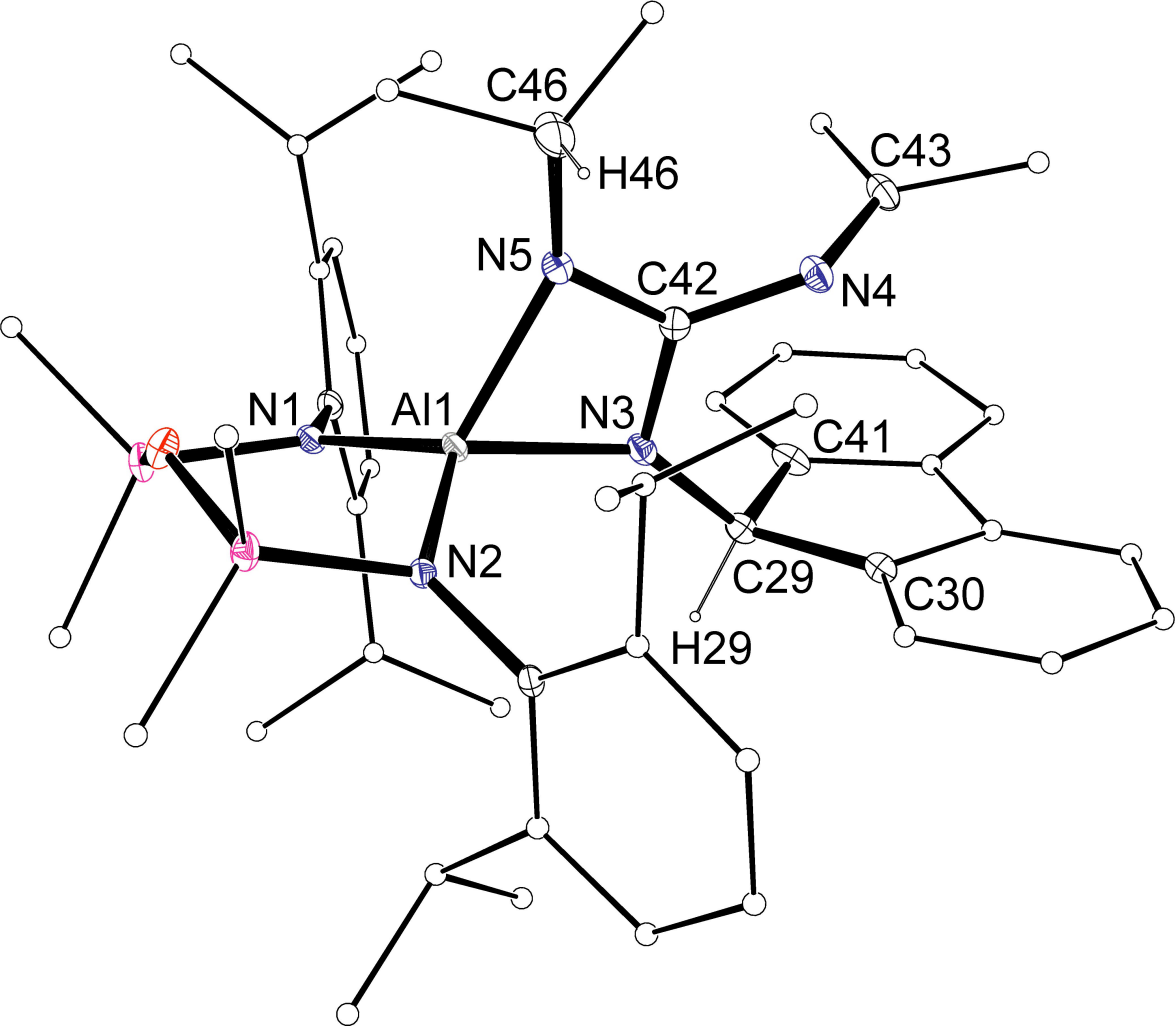
Displacement ellipsoid plot of one of the independent molecules of **4** (30 % ellipsoids; H‐atoms except NC*H*flu omitted; C‐atoms except at key positions represented as spheres). Selected bond lengths (Å) and angles (°) {corresponding value from molecule 2}: Al1‐N1 1.842(4) {1.863(1)}, Al1‐N2 1.867(4) {1.841(4)}, Al1‐N3 1.908(4) {1.901(4)}, Al1‐N5 1.948(4) {1.942(4)}, C42‐N3 1.352(6) {1.354(6)}, C42‐N5 1.334(6) {1.333(6)}, C42‐N4 1.389(6) {1.379(6)}, N4‐C43 1.271(6) {1.253(6)}. N3‐Al−N5 69.83(17) {70.06(16)}, N3‐C42‐N4 122.6(4) {122.5(4)}, N3‐C42‐N5 110.5(4) {110.3(4)}, N4‐C42‐N5 126.0(4) {126.0(4)}, C42‐N4‐C43 129.1(5) {131.4(5)}.

An X‐ray diffraction study of **4** confirmed formation of the κ*N* ^1,2^‐{2‐(9*H*‐fluoren‐9‐yl)‐1‐(isopropyl)‐3‐(propan‐2‐ylidene)guanidinate} ligand at aluminium (Figure 8). The crystal structure contains two essentially identical molecules in the asymmetric unit, each composed of a distorted tetrahedral aluminium centre defined by a NON‐ligand and a chelating, non‐symmetric guanidinate anion. The bond parameters clearly indicate the presence of N‐*i*Pr, N‐(fluH) and C−N=CMe_2_ substituents confirming hydrogen transfer and consistent with NMR data. For example, the N−C bond lengths within the exocyclic propan‐2‐ylidene unit (1.271(6) Å and 1.253(6) Å) specify double bonds, and the sum of bond angles at C43/C91 (360.0°) and for the non‐hydrogen atoms at C29/C77 (334.2° and 334.6°) indicate *sp* ^2^ and *sp* ^3^ hybridization, respectively.

The guanidinates form planar AlN_2_C metallacycles with acute bite angles 69.83(17)° and 70.06(16)° that are typical of such species.[^33^, ^36^] The C−N bond distances in the guanidinate ligand span the range 1.333(6) Å to 1.389(6) Å. These data are consistent with delocalisation throughout the CN_3_ core, with Δ_CN_ values of 0.018 Å and 0.020 Å and Δ’_CN_ of 0.046 Å and 0.035 Å suggesting concentration of π‐electron density within the diazaallyl unit.^[37]^ These data are consistent with other guanidinate anions containing exocyclic ‐N=CR_2_ substituents.^[38]^

**Figure 9:**
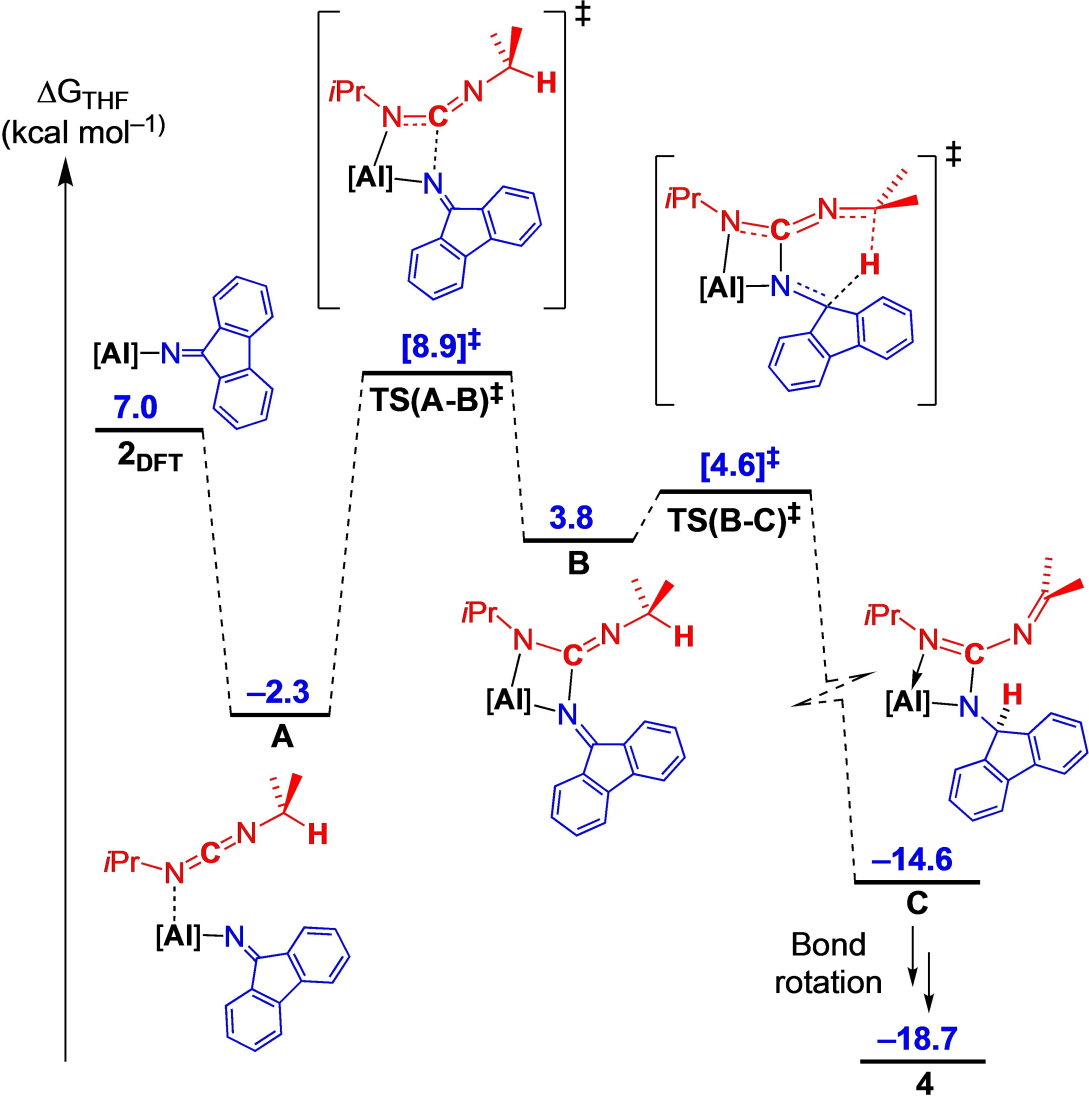
Computed free energy profile (BP86‐D3BJ(PCM=THF)/BS2//BP86/BS1 in kcal mol^−1^) for addition of *i*PrN=C=N*i*Pr to Al(NON)(N=flu) (**2_DFT_**) leading to formation of **4_DFT_**. Note: formal charges omitted for clarity.

The mechanism for the conversion of **2_DFT_** to the guanidinate product **4_DFT_** has been investigated using DFT (Figure 9). The initial approach of the carbodiimide stabilizes the three‐coordinate complex **2_DFT_** by 9.3 kcal mol^−1^ in intermediate **A**, with an Al⋅⋅⋅N_carbodiimide_ distance of 2.13 Å. This distance is reduced to 1.97 Å in the first transition‐state **TS(A‐B)^≠^**, which is rate limiting with a barrier of 11.2 kcal mol^−1^ and involves formation of a C−N bond between the ketimide ligand and the *sp*‐carbon atom of the carbodiimide. These data differ from the previous analysis involving carbodiimide insertion into amide and alkyl bonds, in which a metastable intermediate involving a monodentate κ*N*‐[N(R)C(X)=NR]^−^ (R=carbodiimide substituent; X=amide or alkyl group transferred from Al to *sp*‐carbon atom of the carbodiimide) was identified prior to bond rotation and coordination of the second nitrogen.^[34]^ The resulting structure **B** undergoes a facile hydrogen transfer from an *i*Pr group to the flu‐substituent, with a minimal energy barrier of 0.8 kcal mol^−1^ to afford **C**, at −14.6 kcal mol^−1^, before bond rotations to give **4** at −18.7 kcal mol^−1^.

## Conclusions

The potassium aluminyl K[Al(NON)] reductively couples the diazoalkane derivative 9‐diazo‐9*H*‐fluorene (fluN_2_) to form the intensely coloured compound K[Al(NON){(fluN_2_)_2_}] (**1**) containing a contiguous chain of four nitrogen atoms. This mode of reactivity with R_2_CN_2_ derivatives differs from that of the previously studied aluminyl system, in which a diazo‐aluminium ring was isolated and shown to be a precursor to Al=CR_2_ species.^[12]^ The current reaction most closely resembles that of a Mg(I) dimer with the diazomethane derivatives Ph_2_CN_2_ and fluN_2_, in which each magnesium undergoes a single electron oxidation to form the analogous [R_2_CNNNNCR_2_]^2−^ ligands that bridge between the two metals. X‐ray crystallography showed that in our system the [(fluN_2_)_2_]^2−^ ligand chelates to aluminium through the N‐atoms in the 1‐ and 3‐positions of the chain, to generate a four‐membered AlN_3_ metallacycle. Bond length analysis and density functional theory calculations were consistent with delocalization across the terminal ‘fluNN’ groups, with a single N−N bond linking each of these units.

Various strategies to attenuate the electrostatic interactions between the potassium cation and the Al(NON){(fluN_2_)_2_}]^−^ anion were introduced, including the use of coordinating solvents (THF), 18‐crown‐6 and [2.2.2]cryptand. The resulting modifications had little effect on the solid‐state bonding parameters within the [(fluN_2_)_2_]^2−^ ligands. Furthermore, only minor changes were noted in the UV/vis spectra of each complex in solution, suggesting that the origin of the intense colours for these species originate in the delocalised component of the anion.

Under thermal conditions, loss of N_2_ was observed with the more readily identified product from this reaction being the neutral aluminium ketimide, Al(NON)(N=flu)(L) (**3**), which was isolated as the THF and DMAP derivatives. The fate of the potassium in this reaction was inferred from crystallographic analysis of a sample of highly sensitive crystals that were identified as the doubly reduced 1,2‐di(9*H*‐fluoren‐9‐yl)diazene dianion, which was isolated as the potassium salt, [K_2_(THF)_3_][fluN=Nflu] (**2**).

In contrast to most metal ketimide ligands that are inert towards onward reactivity and are frequently used as spectator ligand in coordination chemistry, the Al−N=flu group in **3** can be reactive. This was demonstrated by adding di*iso*propylcarbodiimide (*i*PrN=C=N*i*Pr) to **3**, which afforded a bidentate guanidinate ligand with ‐*i*Pr and ‐(fluH) substituents at the N‐positions, and a −N=CMe_2_ bonded to the central C‐atom. The mechanism leading to this compound was proposed to involve the initial cycloaddition of a C=N bond of the carbodiimide to the Al‐N_*ketimide*_ bond, followed by hydrogen transfer from an *i*Pr group to the carbon in the 9‐position of the flu‐substituent. This was supported by DFT calculations that identified the cycloaddition as the rate determining step, with a low energy barrier for the hydrogen transfer.

## Experimental Section

Full experimental details and characterisation data of the compounds are provided in the Supporting Information.

### Synthesis of K[Al(NON){(fluN_2_)_2_}] (1)

9‐diazofluorene (76 mg, 0.40 mmol) was suspended in toluene (~5 mL) and added dropwise to a bright yellow solution of K[Al(NON)] (109 mg, 0.20 mmol) in toluene (~5 mL) to give an intense dark blue solution. The solution slowly turns dark green upon storage at room temperature (in the absence of THF). Single crystals were obtained via. slow evaporation of the toluene solution at room temperature. Yield 113 mg, 60 %. Accurate elemental analysis data could not be obtained. ^1^H NMR (500 MHz, C_6_D_6_): *δ* 9.01 (br d, 1H, C_13_ *H_8_*), 8.00–7.51 (m, 7H, C_13_ *H_8_*), 7.42–7.17 (m, 8H, C_13_ *H_8_*), 6.79 (br d, 2H, C_6_ *H_3_*), 6.68–6.40 (m, 4H, C_6_ *H_3_*), 4.26 (br sept, 2H, C*H*Me_2_), 3.80 (br sept, 2H, C*H*Me_2_), 1.68 (br d, 6H, CH*Me_2_*), 1.39 (br d, *J*=6.2, 6H, CH*Me_2_*), 0.87 (br d, 1H, CH*Me_2_*), 0.47 (br d, 6H, CH*Me_2_*), 0.43 (s, 12H, SiMe_2_).

### Synthesis of [K(THF)_5_][Al(NON){(fluN_2_)_2_}] (1⋅THF)

9‐diazofluorene (377 mg, 1.96 mmol) was suspended in toluene (~2 mL) and added dropwise to a bright yellow solution of K[Al(NON)] (539 mg, 0.98 mmol) in toluene (~1 mL). Upon addition, the solution immediately changed to an intense dark blue solution. THF was added dropwise to the reaction mixture to ensure the reaction product was completely dissolved. Single crystals were obtained from the storage of a toluene/THF (~10 : 1, *ca*. 2 mL) solution at −30 °C. Yield 530 mg, 42 %. Anal. Calcd. for C_54_H_62_AlKN_6_OSi_2_ ⋅ (C_4_H_8_O)_5_ (*1293.89*)*: C, 68.69; H, 7.94; N, 6.50 %. Anal. Calcd. for C_54_H_62_AlKN_6_OSi_2_ ⋅ (C_4_H_8_O)_3_ (*1149.68*)^≠^: C, 68.95; H, 7.54; N, 7.31 %. Found: C, 70.12; H, 7.38; N, 6.43 %. (*=loss of 2 x THF solvate; ≠=loss of additional 2 x THF). ^1^H NMR (500 MHz, C_6_D_6_): *δ* 9.03 (d, *J*=7.8, 1H, C_13_ *H_8_*), 7.96 (d, *J*=8.1, 1H, C_13_ *H_8_*), 7.91 (dd, *J*=13.8, 7.7, 2H, C_13_ *H_8_*), 7.80 (dd, *J*=7.5, 4.1, 2H, C_13_ *H_8_*), 7.76 (d, *J*=7.5, 1H, C_13_ *H_8_*), 7.67–7.61 (m, 2H, C_6_ *H_5_*), 7.36–7.25 (m, 6H, C_13_ *H_8_*), 7.19 (td, *J*=7.4, 3.0, 2H, C_13_ *H_8_*), 6.82 (d, *J*=7.50, 2H, C_6_ *H_3_*), 6.69–6.43 (m, 4H, C_6_ *H_3_*), 4.29 (sept, *J*=6.4, 2H, C*H*Me_2_), 3.82 (sept, *J*=6.4, 2H, C*H*Me_2_), 3.48 (s, 12H, THF), 1.71 (br d, 6H, CH*Me_2_*), 1.41 (d, *J*=6.8, 6H, CH*Me_2_*), 1.38 (s, 12H, THF) 0.89 (br d, 6H, CH*Me_2_*), 0.52–0.46 (m, 12H, CH*Me_2_*, SiMe_2_)*, 0.44 (s, 6H, SiMe_2_). ^13^C{^1^H} NMR (126 MHz, C_6_D_6_): *δ* 140.5, 137.7, 136.1, 136.0, 135.4, 134.7, 133.0, 130.9, 129.8, 127.0, 126.8, 126.4, 126.0, 125.3, 125.2, 124.9, 124.1, 124.0, 123.9, 123.7, 123.2, 120.6, 120.4, 119.9, 119.8, 118.9, 117.0 (*C_13_* H_8_, *C_6_* H_3_), 67.8 (THF) 28.3 (*C*HMe_2_), 27.9 (CH*Me_2_*), 27.5 (*C*HMe_2_), 27.0 (CH*Me_2_*), 25.8 (THF), 25.5, 24.9, 23.1 (CH*Me_2_*), 3.7, 3.0 (Si*Me_2_*). * Overlapping signals.

### Synthesis of [K(18‐c‐6)][Al(NON){(fluN_2_)_2_}] (1⋅crown)

9‐diazofluorene (47 mg, 0.24 mmol) was suspended in hexane (~1 mL) and added dropwise to a bright yellow solution of K[Al(NON)] (67 mg, 0.12 mmol) in hexane (~1 mL) to give an intense dark blue suspension. The solvent was reduced *in vacuo* and the residue dissolved in THF (~1 mL). A solution of 18‐crown‐6 (32 mg, 0.12 mmol) in THF (~1 mL) was added to this solution. Crystals were obtained from a THF solution (~2 mL) stored at −30 °C. Yield 60 mg, 41 %. Anal. Calcd. for C_66_H_86_AlKN_6_O_7_Si_2_ ⋅ C_4_H_8_O (*1269.78*)*: C, 66.21; H, 7.46; N, 6.62 %. Found: C, 66.16; H, 7.29; N, 6.07 %. (*=calculated for 1 x THF solvate). ^1^H NMR (500 MHz, C_6_D_6_): *δ* 9.26 (d, *J*=7.8, 1H, C_13_ *H_8_*), 8.54 (d, *J*=8.1, 1H, C_13_ *H_8_*), 8.16 (d, *J*=7.8, 1H, C_13_ *H_8_*), 8.08–8.03 (m, 2H, C_13_ *H_8_*), 8.00 (d, *J*=7.6, 1H, C_13_ *H_8_*/C_6_ *H_3_*), 7.91 (d, *J*=7.6, 1H, C_13_ *H_8_*), 7.85 (d, *J*=7.5, 1H, C_13_ *H_8_*), 7.78–7.73 (m, 1H, C_13_ *H_8_*), 7.48–7.42 (m, 2H, C_13_ *H_8_*), 7.36 (dt, *J*=13.1, 7.4, 3H, C_13_ *H_8_*), 7.28–7.23 (m, 1H, C_13_ *H_8_*), 7.18 (d, *J*=7.0, 1H, C_13_ *H_8_*), 7.11 (d, *J*=7.6, 2H, C_6_ *H_3_*), 6.87 (d, *J*=7.6, 2H, C_6_ *H_3_*), 6.79 (t, *J*=7.6, 2H, C_6_ *H_3_*), 4.58 (sept, *J*=6.8, 2H, C*H*Me_2_), 4.05 (sept, *J*=6.8, 2H, C*H*Me_2_), 3.64–3.51 (m, 6H, THF), 2.56 (s, 24H, crown‐C*H_2_*), 1.98 (d, *J*=6.7, 6H, CH*Me_2_*), 1.66 (d, *J*=6.6, 6H, CH*Me_2_*), 1.52–1.35 (m, 6H, THF), 1.01 (d, *J*=6.6, 6H, CH*Me_2_*), 0.71 (d, *J*=6.4, 6H, CH*Me_2_*), 0.59 (s, 6H, Si*Me_2_*), 0.51 (s, 6H, Si*Me_2_*). ^13^C{^1^H} NMR (126 MHz, C_6_D_6_): *δ* 148.3, 147.7, 142.4, 141.0, 137.1, 135.1, 135.0, 134.6, 133.1, 132.6, 130.9, 130.8, 126.2, 125.9, 125.5, 124.9, 124.0, 123.9, 123.6, 123.4, 123.3, 121.2, 120.7, 120.5, 120.3, 120.2, 119.7, 118.5 (*C_13_* H_8_, *C_6_* H_3_), 69.6 (crown‐*C*H_2_), 67.8 (THF), 29.0 (CH*Me_2_*), 28.6 (*C*HMe_2_), 27.8 (CH*Me_2_*), 27.7 (*C*HMe_2_), 25.8 (THF), 25.2, 24.7 (CH*Me_2_*), 3.6, 2.9 (Si*Me_2_*).

### Synthesis of [K([2.2.2]crypt)]][Al(NON){(fluN_2_)_2_}] (1⋅crypt)

**Procedure A**: 9‐diazofluorene (42 mg, 0.22 mmol) was suspended in hexane (~1 mL) and added dropwise to a bright yellow solution of K[Al(NON)] (60 mg, 0.11 mmol) in hexane (~1 mL) to give an intense dark blue suspension. The solvent was removed *in vacuo* and the residue dissolved in THF (~1 mL). A solution of [2.2.2]cryptand (41 mg, 0.11 mmol) in THF (~1 mL) was added. Crystals were obtained from a THF solution (~2 mL) stored at −30 °C. Yield 110 mg, 76 %. Attempted analysis by NMR spectroscopy in C_6_D_6_ was hampered by low solubility and redissolving the crystalline product in THF‐D_8_ resulted in decomposition. A small number of crystals precipitated from the C_6_D_6_ NMR sample and were analysed by X‐ray diffraction, confirming formation of [K([2.2.2]crypt)]][Al(NON){(fluN_2_)_2_}].

**Procedure B**: A dark blue powder of **1⋅THF** (81 mg, 0.06 mmol) and 2.2.2‐cryptand (24 mg, 0.06 mmol) were combined in THF (~1 mL). Crystals were obtained from a THF solution (~2 mL) stored at −30 °C. Yield 52 mg, 66 %.

### Synthesis of Al(NON)(N=flu)(THF) (2⋅THF)

A solution of **1⋅THF** (103 mg, 0.08 mmol) in benzene‐d_6_ was prepared in a glovebox and transferred to a J. Youngs NMR tube and sealed. The tube was removed from the glovebox and placed in a steel heating block. The solution was heated overnight (*ca*. 18 h) at 80 °C to give a dark green solution. ^1^H NMR showed full consumption of the starting material. The NMR tube was taken into a glovebox and the tap removed slowly to relieve pressure (from nitrogen evolution). The contents of the NMR tube were transferred to a scintillation vial and the solvent removed *in vacuo*. The residue was dissolved in toluene (*ca*. 1 mL) to give a dark green solution and a few drops of THF were added. Crystals were obtained from a toluene solution of the reaction mixture stored at −30 °C. Yield 53 mg, 83 %. Accurate elemental analysis data could not be obtained. ^1^H NMR (500 MHz, C_6_D_6_): *δ* 7.30–7.18 (m, 8H, C_6_ *H_3_*, C_13_ *H_8_*), 7.09 (t, *J*=7.4, 2H, C_13_ *H_8_*)*, 7.05–7.00 (m, 2H, C_13_ *H_8_*)*, 6.27 (d, *J*=7.4, 2H, C_13_ *H_8_*), 3.99 (br sept, 2H, C*H*Me_2_), 3.87 (s, 2H, THF), 3.81 (br sept, *J*=7.2, 2H, C*H*Me_2_), 3.50 (s, 2H, THF), 1.42 (br d, 6H, CH*Me_2_*), 1.38–1.37 (m, 4H, THF), 1.33 (br d, 6H, CH*Me_2_*), 1.02 (br d, 6H, CH*Me_2_*), 0.99 (br d, 6H, CH*Me_2_*), 0.43 (s, 6H, Si*Me_2_*), 0.40 (s, 6H, Si*Me_2_*). * ~1 equivalent of toluene present in sample (indicated by peak at *δ* 2.11 integrating for 3H), which overlaps with aromatic peaks between *δ* 7.02–7.13. ^13^C{^1^H} NMR (126 MHz, C_6_D_6_): *δ* 166.5, 148.4, 146.3, 144.3, 143.5, 136.8, 130.9, 125.1, 123.8, 123.4, 122.8, 119.5 (*C_13_* H_8_/*C_6_* H_3_), 72.4, 67.7 (THF), 28.1, 27.7 (*C*HMe_2_), 27.4†, 25.4, 25.2 (CH*Me_2_*), 3.7 (Si*Me_2_*). † Two overlapping resonances appearing as one signal.

### Synthesis of Al(NON)(N=flu)(DMAP) (2⋅DMAP)

A solution of **1⋅THF** (56 mg, 0.04 mmol) in benzene‐d_6_ was prepared in a glovebox and transferred to a J. Youngs NMR tube and sealed. The tube was removed from the glovebox and placed in a steel heating block. The solution was heated overnight (*ca*. 18 h) at 80 °C to give a dark green solution. ^1^H NMR spectrum showed full consumption of the starting material. The NMR tube was taken into a glovebox and the tap removed slowly to relieve pressure (from nitrogen evolution). DMAP (5 mg, 0.04 mmol) was added to the reaction mixture and the NMR tube sealed and mixed. The contents of the NMR tube were then transferred to a scintillation vial and the solvent removed *in vacuo*. The residue was dissolved in hexane (*ca*. 1 mL) to give a dark green solution. Crystals were obtained from a hexane solution of the reaction mixture stored at room temperature. Yield 30 mg. Analysis by NMR showed formation of an inseparable mixture of unidentified products, from which single crystals of Al(NON)(N=flu)(DMAP) were separated and analysed by X‐ray diffraction.

### Synthesis of [K_2_(THF)_3_][(fluN)_2_] (3)

9‐diazofluorene (63 mg, 0.33 mmol) and K[Al(NON)] (91 mg, 0.17 mmol) were suspended in C_6_D_6_ (~0.6 mL) to give an intense dark blue solution. The solution was heated overnight (*ca*. 18 h) at 80 °C to give a dark green solution. The NMR tube was taken into a glovebox and the tap removed slowly to relieve pressure (from nitrogen evolution). DMAP (5 mg, 0.04 mmol) was added to the reaction mixture and the NMR tube sealed and mixed. The contents of the NMR tube were then transferred to a scintillation vial and the solvent removed *in vacuo*. The residue was washed with hexane (5 x 2 mL) and a toluene/THF mixture (1 mL, 1 : 1) was added. A small quantity of crystals of [K_2_(THF)_3_][(fluN)_2_] (**3**) that co‐crystallized with **2⋅THF** were separated and characterised by single‐crystal X‐ray diffraction. It was not possible to obtain any additional analysis on these crystals.

### Synthesis of Al(NON){iPrNC(N=CMe_2_)NC(H)flu)} (4)

A solution of **1⋅THF** (83 mg, 0.07 mmol) in benzene‐D_6_ was transferred to J Youngs NMR tube and heated to 80 °C for 18 h. The pressure of the NMR tube from liberation of nitrogen was relieved in a nitrogen‐filled glovebox, and a solution of *N,N’*‐di*iso*propylcarbodiimide (9 mg, 0.07 mmol) was added. Reaction progress was monitored by ^1^H NMR spectroscopy and observed to be complete within 15 min of addition. The solution was transferred to a scintillation vial and solvent reduced *in vacuo* to give dark green residue. The residue was washed with hexane (3 times, 5 mL) and then dried. Toluene (~2 mL) was then added to the residue and a few drops of THF to ensure complete dissolution. Storage of the toluene/THF mixture at −30 °C yielded dark blue crystals suitable for single‐crystal X‐ray diffraction experiments. Yield 17 mg, 31 %. Accurate elemental analysis data could not be obtained. ^1^H NMR (500 MHz, C_6_D_6_): *δ* 7.34 (dd, *J*=6.8, 3.0, 2H, C_6_ *H_3_*), 7.30–7.24 (m, 4H, C_6_ *H_3_*), 7.18 (d, *J*=7.8, 2H, C_13_ *H_8_*), 6.98 (t, *J*=7.4, 2H, C_13_ *H_8_*), 6.92 (t, *J*=7.4, 2H, C_13_ *H_8_*), 5.83 (d, *J*=7.4, 2H, C_13_ *H_8_*), 5.07 (s, 1H, Flu‐C*H*), 4.29 (sept, *J*=6.8, 2H, C*H*Me_2_), 4.11 (sept, *J*=6.8, 2H, C*H*Me_2_), 3.53 (s, 6H, THF), 3.37 (sept, *J*=6.6, 1H, C*H*Me_2_), 1.50 (d, *J*=6.8, 6H, CH*Me_2_*), 1.45 (d, *J*=6.8, 6H, CH*Me_2_*), 1.40 (s, 6H, THF), 1.29–1.23 (m, 18H, CH*Me_2_*), 0.59 (s, 6H, NC*Me_2_*), 0.54 (s, 6H, SiMe_2_), 0.38 (s, 6H, Si*Me_2_*). ^13^C{^1^H} NMR (126 MHz, C_6_D_6_): *δ* 170.3 (N*C*N), 169.8 (N*C*Me_2_), 147.5, 147.0, 146.3, 143.5, 139.7, 129.3, 128.6, 127.8*, 127.5, 126.7, 125.0, 124.7, 123.4, 119.0 (*C_6_* H_3_, *C_13_* H_8_), 67.8 (THF), 59.5 (Flu‐*C*H), 45.5 (*C*HMe_2_), 28.5 (*C*HMe_2_), 28.4 (CH*Me_2_*), 27.7 (*C*HMe_2_), 27.6 (CH*Me_2_*), 26.2, 26.1 (CH*Me_2_*), 25.8 (THF), 25.7 (*C*Me_2_), 24.8 (CH*Me_2_*), 4.7, 3.2 (Si*Me_2_*). * Quaternary ^13^C signal overlapping with residual solvent peak.

## Supporting Information

The authors have cited additional references within the Supporting Information.[^39^, ^40^, ^41^, ^42^, ^43^, ^44^, ^45^, ^46^, ^47^, ^48^, ^49^, ^50^, ^51^]

Deposition Number(s) 2292504–2292511 contain(s) the supplementary crystallographic data for this paper. These data are provided free of charge by the joint Cambridge Crystallographic Data Centre and Fachinformationszentrum Karlsruhe Access Structures service www.ccdc.cam.ac.uk/structures.

## Conflict of interest

The authors declare no conflict of interest.

1

## Supporting information

As a service to our authors and readers, this journal provides supporting information supplied by the authors. Such materials are peer reviewed and may be re‐organized for online delivery, but are not copy‐edited or typeset. Technical support issues arising from supporting information (other than missing files) should be addressed to the authors.

Supporting Information

## Data Availability

The data that support the findings of this study are available from the corresponding author upon reasonable request.
