# Supplementary material for: Reductive Coupling of a Diazoalkane Derivative Promoted by a Potassium Aluminyl and Elimination of Dinitrogen to Generate a Reactive Aluminium Ketimide
Source: Chemistry. 2023 Nov 7;29(71):e202302903. doi: 10.1002/chem.202302903 (PMC10946750; doi:10.1002/chem.202302903)
Supplement: Supplementary file 1 — Supporting Information [file CHEM-29-0-s001.pdf]

Chemistry–A European Journal

Supporting Information

Reductive Coupling of a Diazoalkane Derivative Promoted by a Potassium Aluminyl and Elimination of Dinitrogen to Generate a Reactive Aluminium Ketimide

Matthew J. Evans, Mathew D. Anker, Claire L. McMullin,* and Martyn P. Coles*

S2 General Experimental Procedures
S2 Synthesis of K[Al(NON){(fluN$_2$)$_2$}] (**1**)
S3 **Figure S1** ^{1}H NMR spectrum of **1**
S4 **Figure S2** UV/vis spectrum of **1**
S5 **Figure S3** Displacement ellipsoid plot of the asymmetric unit of **1**
S6 Synthesis of K[Al(NON){(fluN$_2$)$_2$}] (**1·THF**)
S7 **Figure S4** ^{1}H NMR spectrum of **1·THF**
S8 **Figure S5** ^{13}C{^{1}H} NMR spectrum of **1·THF**
S9 **Figure S6** UV/vis spectrum of **1·THF**
S10 **Figure S7** Displacement ellipsoid plot of the asymmetric unit of **1·THF**
S11 **Figure S8** Displacement ellipsoid plot of the grown molecule of **1·THF**
S12 Synthesis of [K(18-c-6)][Al(NON){(fluN$_2$)$_2$}] (**1·crown**)
S13 **Figure S9** ^{1}H NMR spectrum of **1·crown**
S14 **Figure S10** ^{13}C{^{1}H} NMR spectrum of **1·crown**
S15 **Figure S11** UV/vis spectrum of **1·crown**
S16 Synthesis of [K([2.2.2]crypt)]][Al(NON){(fluN$_2$)$_2$}] (**1·crypt**)
S17 **Figure S12** Displacement ellipsoid plot of the asymmetric unit of **1·crypt**
S18 Synthesis of Al(NON)(N=flu)(THF) (**2·THF**)
S19 **Figure S13** ^{1}H NMR spectrum of **2·THF**
S20 **Figure S14** ^{13}C{^{1}H} NMR spectrum of **2·THF**
S21 **Figure S15** Variable temperature ^{1}H NMR spectra of **2·THF**
S22 **Figure S16** UV/vis spectrum of **2·THF**
S23 **Figure S17** Displacement ellipsoid plot of the asymmetric unit of **2·THF{THF}**
S24 **Figure S18** Displacement ellipsoid plot of the asymmetric unit of **2·THF{Ar}**
S25 Synthesis of Al(NON)(N=flu)(DMAP) (**2·DMAP**)
S26 **Figure S19** ^{1}H NMR spectra plotting the formation of **2·DMAP**
S27 **Figure S20** Displacement ellipsoid plot of the asymmetric unit of **2·DMAP**
S28 Synthesis of [K$_2$(THF)$_3$][(fluN)$_2$] (**3**)
S29 **Figure S21** Displacement ellipsoid plot of the asymmetric unit of **3**
S30 Synthesis of Al(NON){*i*PrNC(N=CMe$_2$)NC(H)flu)} (**4**)
S31 **Figure S22** ^{1}H NMR spectrum of **4**
S32 **Figure S23** ^{13}C{^{1}H} NMR spectrum of **4**
S33 **Figure S24** Displacement ellipsoid plot of the asymmetric unit of **4**
S34 Crystallographic details
S35 **Table S1** Crystal structure and refinement data for **1**, **1·THF** and **1·crypt**
S36 **Table S2** Crystal structure and refinement data for **2·THF{THF}**, **2·THF{Ar}** and **2·DMAP**
S37 **Table S3** Crystal structure and refinement data for **3** and **4**
S38 Computational details
S39 **Table S4** Relative energies for computed structures
S40 **Figure S25** Natural charges and WBI data for **1$_{DFT}$** and **1'$_{DFT}$**
S40 **Table S5** Selected NBO data for **1'$_{DFT}$**
S41 **Figure S26** Natural charges and WBI data for **2·THF$_{DFT}$** and **2$_{DFT}$**
S42 **Table S6** Selected NBO data for **2$_{DFT}$**
S43 Cartesian Coordinates and Computed Energies for Calculated Structures

General Experimental Procedures

All manipulations were performed under dry nitrogen or argon using standard Schlenk-line techniques, or in a conventional nitrogen-filled glovebox. Hexane, toluene, diethyl ether (Et_2O), and tetrahydrofuran (THF) were obtained from a PureSolv MD 5 system and stored over activated 5Å molecular sieves for 24 hours prior to use. NMR spectra were recorded using a Jeol JNM-ECZ500S 500 MHz spectrometer equipped with a ROYAL digital auto tune probe S, operating at 500.1 (^{1}H), 125.8 (^{13}C) MHz. Spectra were recorded at 294 K (unless stated otherwise) and proton and carbon chemical shifts were referenced internally to residual solvent resonances. Coupling constants are quoted in Hz. UV-Vis spectra were recorded on a Cary50 spectrometer equipped with a Xenon lamp. Elemental analyses were carried out by the Elemental Analysis Services team at London Metropolitan University. K[Al(NON)][4a] and diazofluorene[39] were prepared according to the literature procedures. All other chemicals were purchased from Sigma-Aldrich and used without further purification.

Synthesis of K[Al(NON){(fluN$_2$)$_2$}] (1)

9-diazofluorene (76 mg, 0.40 mmol) was suspended in toluene (~5 mL) and added dropwise to a bright yellow solution of K[Al(NON)] (109 mg, 0.20 mmol) in toluene (~5 mL) to give an intense dark blue solution. The solution slowly turns dark green upon storage at room temperature (in the absence of THF). Single crystals were obtained *via.* slow evaporation of the toluene solution at room temperature. Yield 113 mg, 60 %.

^{1}H NMR (500 MHz, C_6D_6): δ 9.01 (br d, 1H, $C_{13}H_8$), 8.00 – 7.51 (m, 7H, $C_{13}H_8$), 7.42 – 7.17 (m, 8H, $C_{13}H_8$), 6.79 (br d, 2H, C_6H_3), 6.68 – 6.40 (m, 4H, C_6H_3), 4.26 (br sept, 2H, C*H*Me$_2$), 3.80 (br sept, 2H, C*H*Me$_2$), 1.68 (br d, 6H, CH*Me$_2$*), 1.39 (br d, J = 6.2, 6H, CH*Me$_2$*), 0.87 (br d, 1H, CH*Me$_2$*), 0.47 (br d, 6H, CH*Me$_2$*), 0.43 (s, 12H, SiMe$_2$).

No meaningful ^{13}C NMR data could be obtained due to broadness of signals.

We were unable to obtain accurate elemental analysis for this compound.

Figure S1 ^{1}H NMR Spectrum (500 MHz, C_6D_6) of K[Al(NON){(fluN$_2$)$_2$}] (**1**)

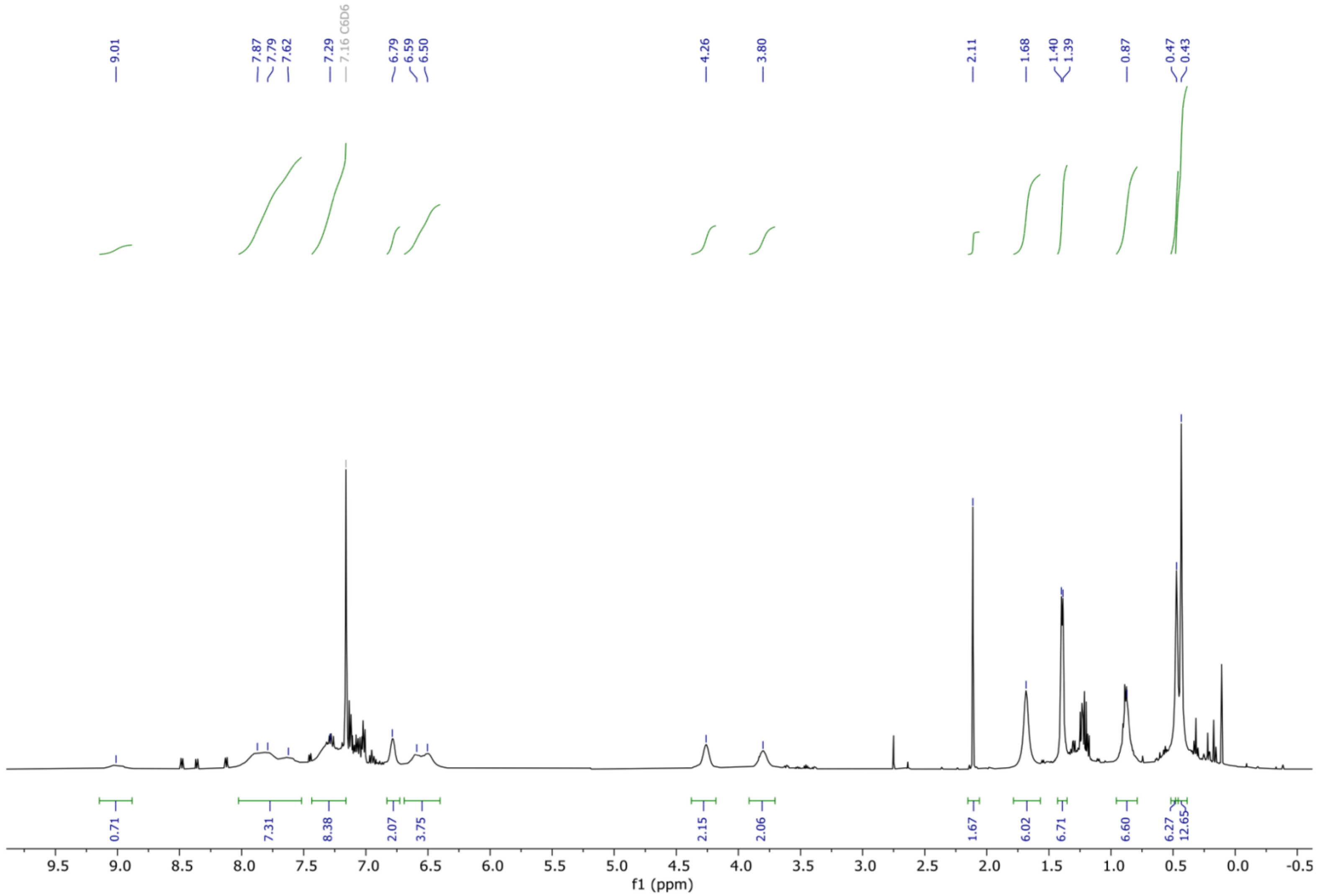

Figure S2 UV/vis Spectrum of K[Al(NON){(fluN$_2$)$_2$}] (**1**)

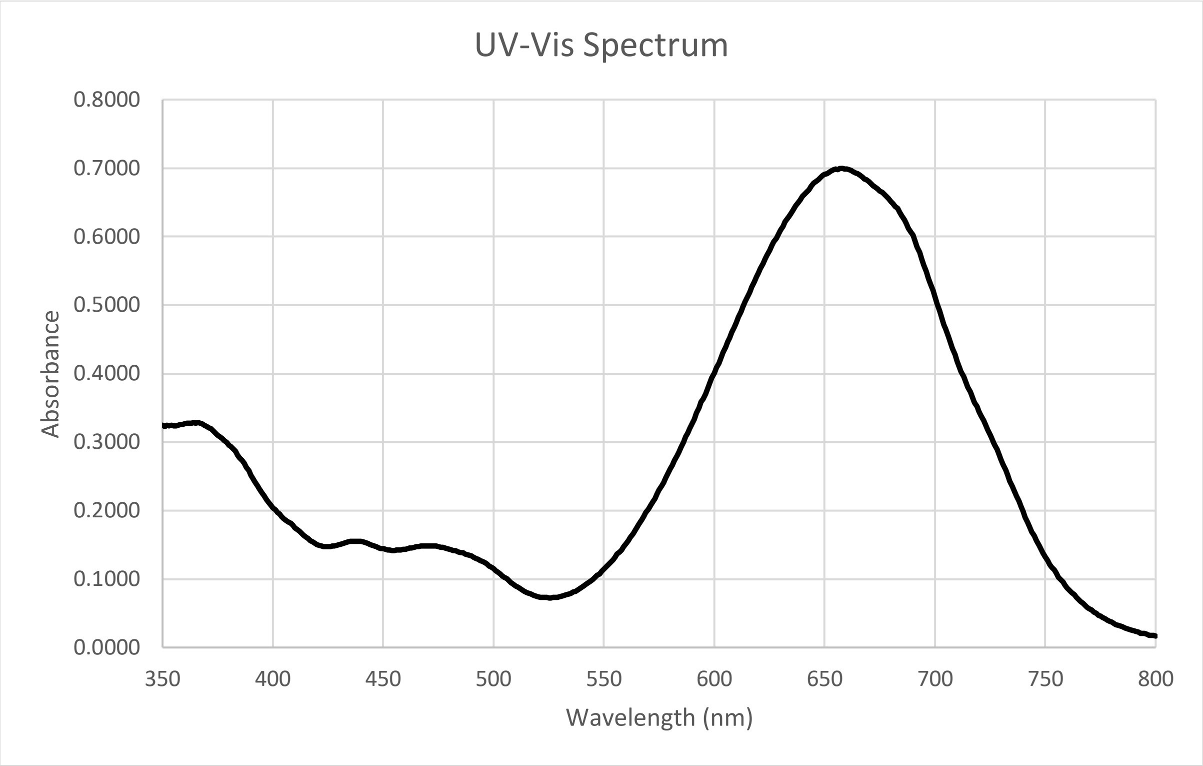

**UV-Vis Details**		
λ_{max}	Absorbance	ε (L mol^{-1} cm^{-1})
657 nm	0.6993	16138
470 nm	0.1486	3429
437 nm	0.1555	3589
Solvent: Toluene<br>Concentration of Sample: 4.33 x 10^{-5} mol L^{-1} (V = 3.56 mL)<br>Observed Colour: Blue-Green (Intense)		

Figure S3 Displacement ellipsoid plot (30 % probability, H-atoms omitted) of the asymmetric unit of K[Al(NON){(fluN$_2$)$_2$}] (**1**)

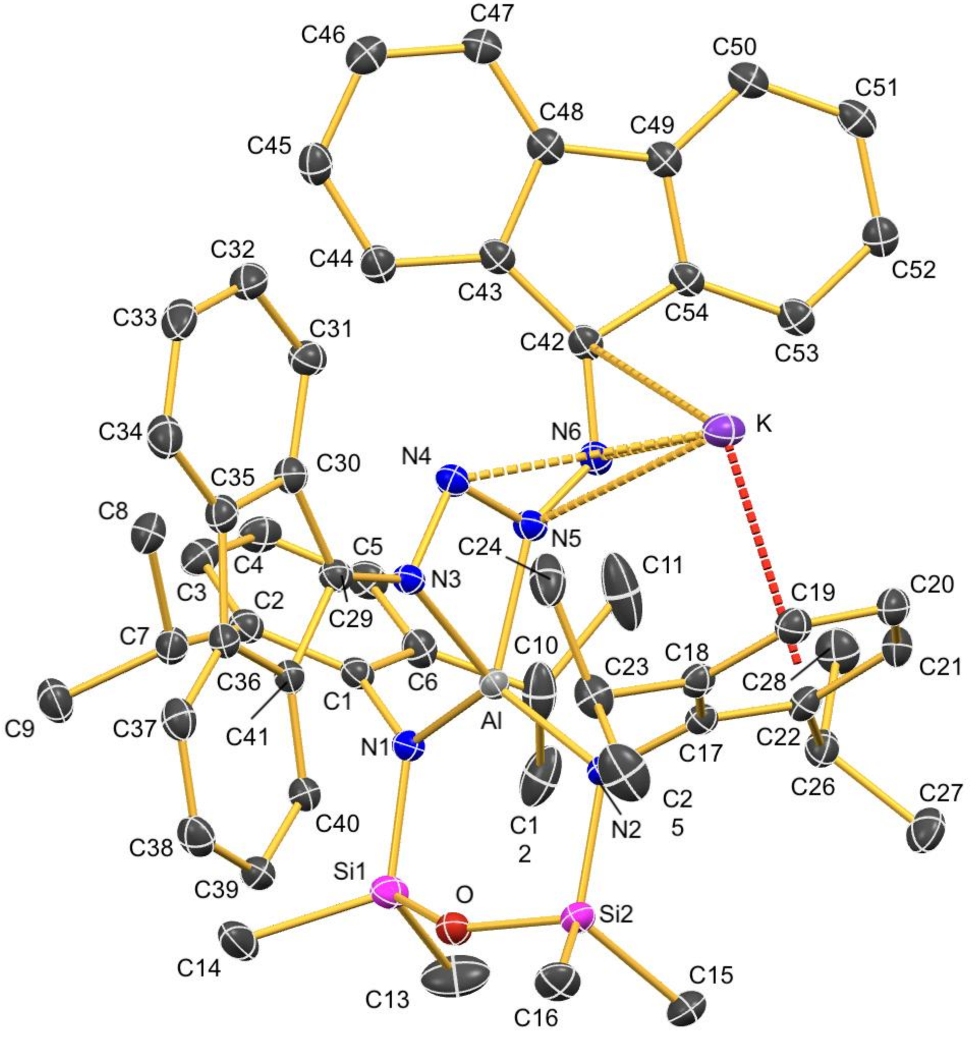

Synthesis of [K(THF)$_5$][Al(NON){(fluN$_2$)$_2$}] (1·THF)

9-diazofluorene (377 mg, 1.96 mmol) was suspended in toluene (~2 mL) and added dropwise to a bright yellow solution of K[Al(NON)] (539 mg, 0.98 mmol) in toluene (~1 mL). Upon addition, the solution immediately changed to an intense dark blue solution. THF was added dropwise to the reaction mixture to ensure the reaction product was completely dissolved. Single crystals were obtained from the storage of a toluene/THF (~10:1, *ca.* 2 mL) solution at –30 °C. Yield 530 mg, 42 %.

Anal. Calcd. for $C_{54}H_{62}AlKN_6OSi_2·(C_4H_8O)_5$ (*1293.89*)*: C, 68.69; H, 7.94; N, 6.50 %. Anal. Calcd. for $C_{54}H_{62}AlKN_6OSi_2·(C_4H_8O)_3$ (*1149.68*)‡: C, 68.95; H, 7.54; N, 7.31 %. Found: C, 70.12; H, 7.38; N, 6.43 %. (* = loss of 2 x THF solvate; ‡ = loss of additional 2 x THF).

^{1}H NMR (500 MHz, C_6D_6): δ 9.03 (d, J = 7.8, 1H, $C_{13}H_8$), 7.96 (d, J = 8.1, 1H, $C_{13}H_8$), 7.91 (dd, J = 13.8, 7.7, 2H, $C_{13}H_8$), 7.80 (dd, J = 7.5, 4.1, 2H, $C_{13}H_8$), 7.76 (d, J = 7.5, 1H, $C_{13}H_8$), 7.67 – 7.61 (m, 2H, C_6H_5), 7.36 – 7.25 (m, 6H, $C_{13}H_8$), 7.19 (td, J = 7.4, 3.0, 2H, $C_{13}H_8$), 6.82 (d, J = 7.50, 2H, C_6H_3), 6.69 – 6.43 (m, 4H, C_6H_3), 4.29 (sept, J = 6.4, 2H, C*H*Me$_2$), 3.82 (sept, J = 6.4, 2H, C*H*Me$_2$), 3.48 (s, 12H, THF), 1.71 (br d, 6H, CH*Me$_2$*), 1.41 (d, J = 6.8, 6H, CH*Me$_2$*), 1.38 (s, 12H, THF) 0.89 (br d, 6H, CH*Me$_2$*), 0.52 – 0.46 (m, 12H, CH*Me$_2$*, SiMe$_2$)*, 0.44 (s, 6H, SiMe$_2$).

^{13}C{^{1}H} NMR (126 MHz, C_6D_6): δ 140.5, 137.7, 136.1, 136.0, 135.4, 134.7, 133.0, 130.9, 129.8, 127.0, 126.8, 126.4, 126.0, 125.3, 125.2, 124.9, 124.1, 124.0, 123.9, 123.7, 123.2, 120.6, 120.4, 119.9, 119.8, 118.9, 117.0 (*C$_{13}$*H$_8$, *C$_6$*H$_3$), 67.8 (THF) 28.3 (*C*HMe$_2$), 27.9 (CH*Me$_2$*), 27.5 (*C*HMe$_2$), 27.0 (CH*Me$_2$*), 25.8 (THF), 25.5, 24.9, 23.1 (CH*Me$_2$*), 3.7, 3.0 (Si*Me$_2$*).

*Overlapping signals.

Figure S4 ^{1}H NMR Spectrum (500 MHz, C_6D_6) of $[K(THF)_5][Al(NON)\{(fluN_2)_2\}]$ (**1·THF**)

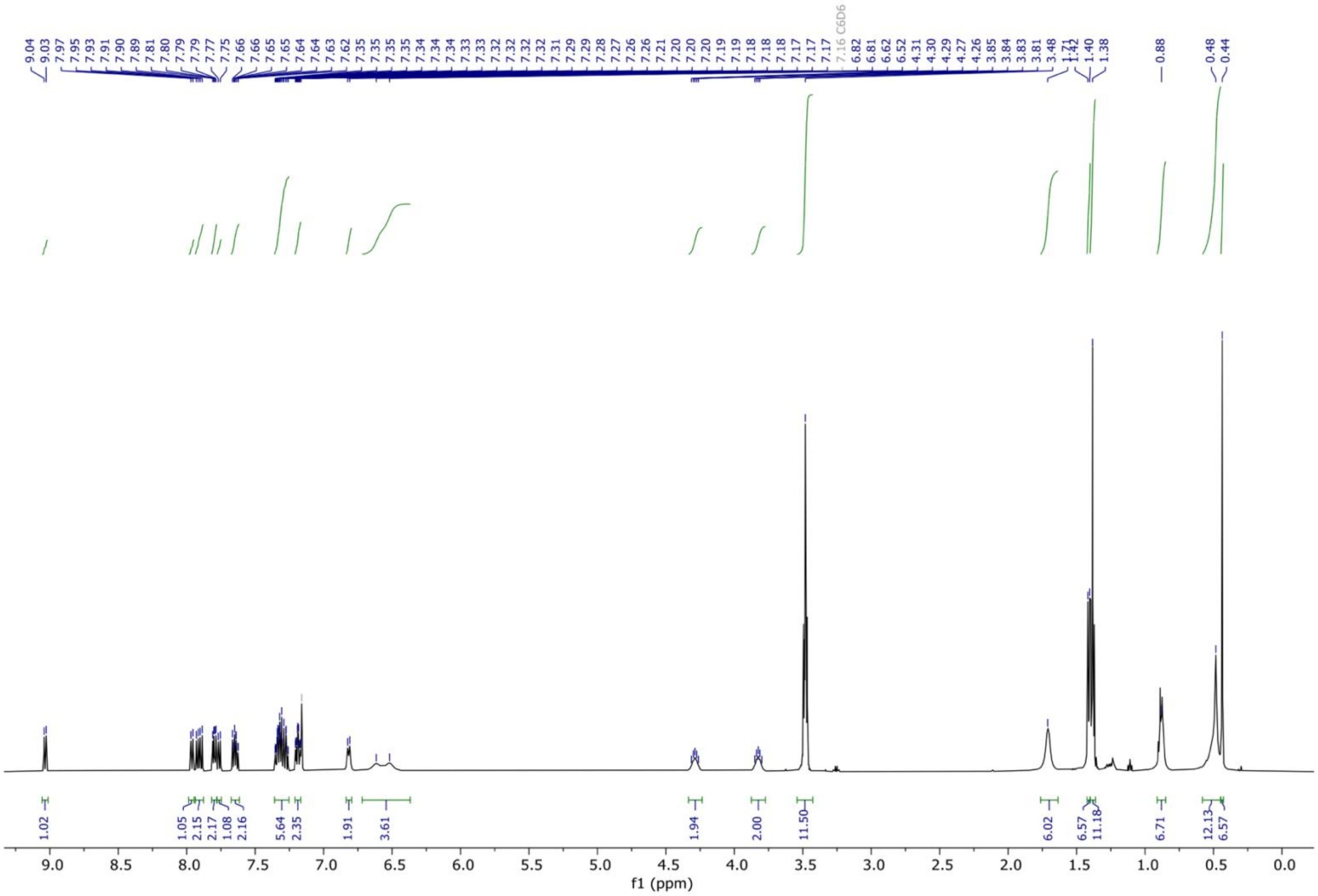

Figure S5 $^{13}C\{^{1}H\}$ NMR Spectrum (126 MHz, C_6D_6) of $[K(THF)_5][Al(NON)\{(fluN_2)_2\}]$ (**1·THF**)

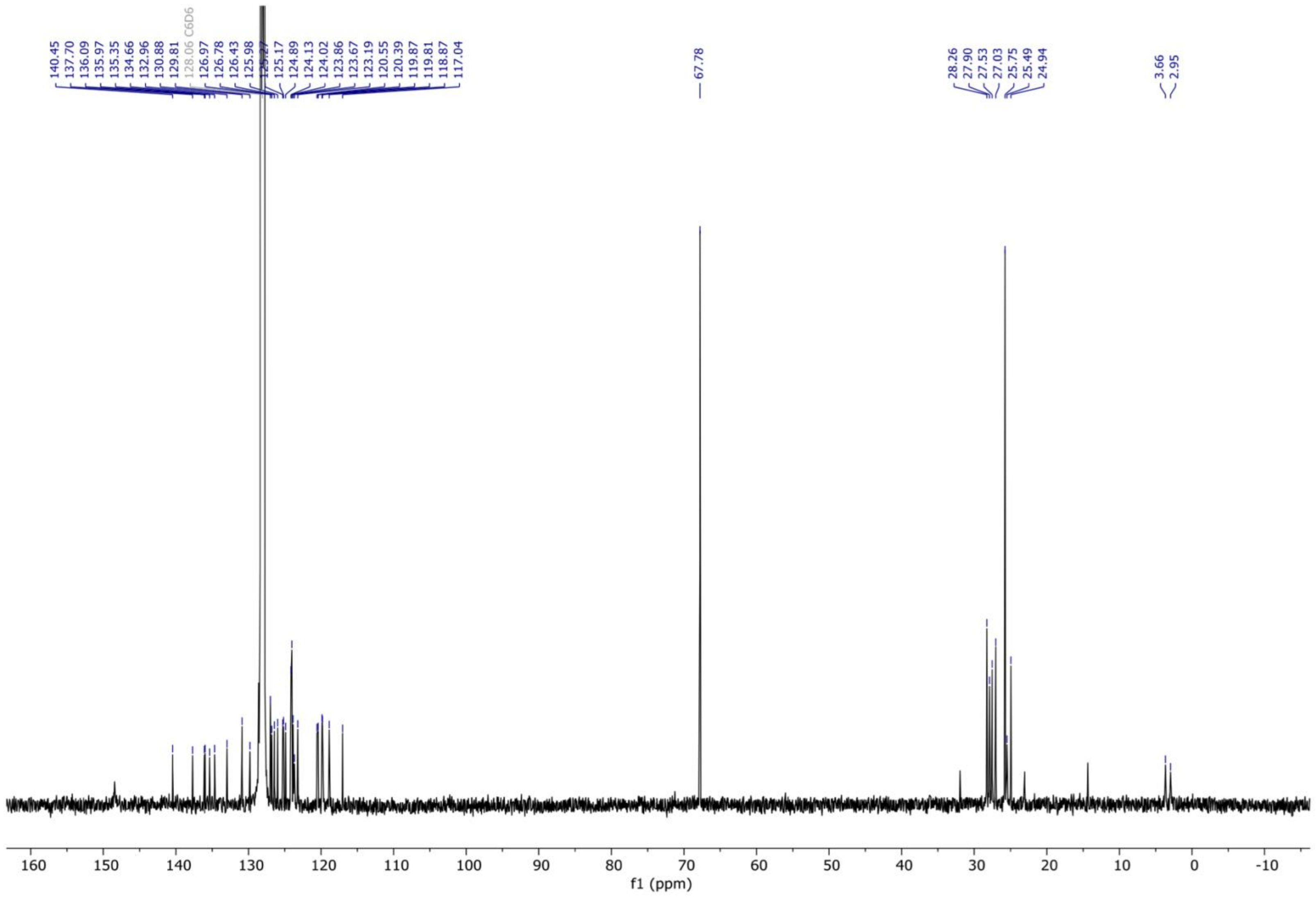

Figure S6 UV/vis Spectrum of [K(THF)$_5$][Al(NON){(fluN$_2$)$_2$}] (**1·THF**)

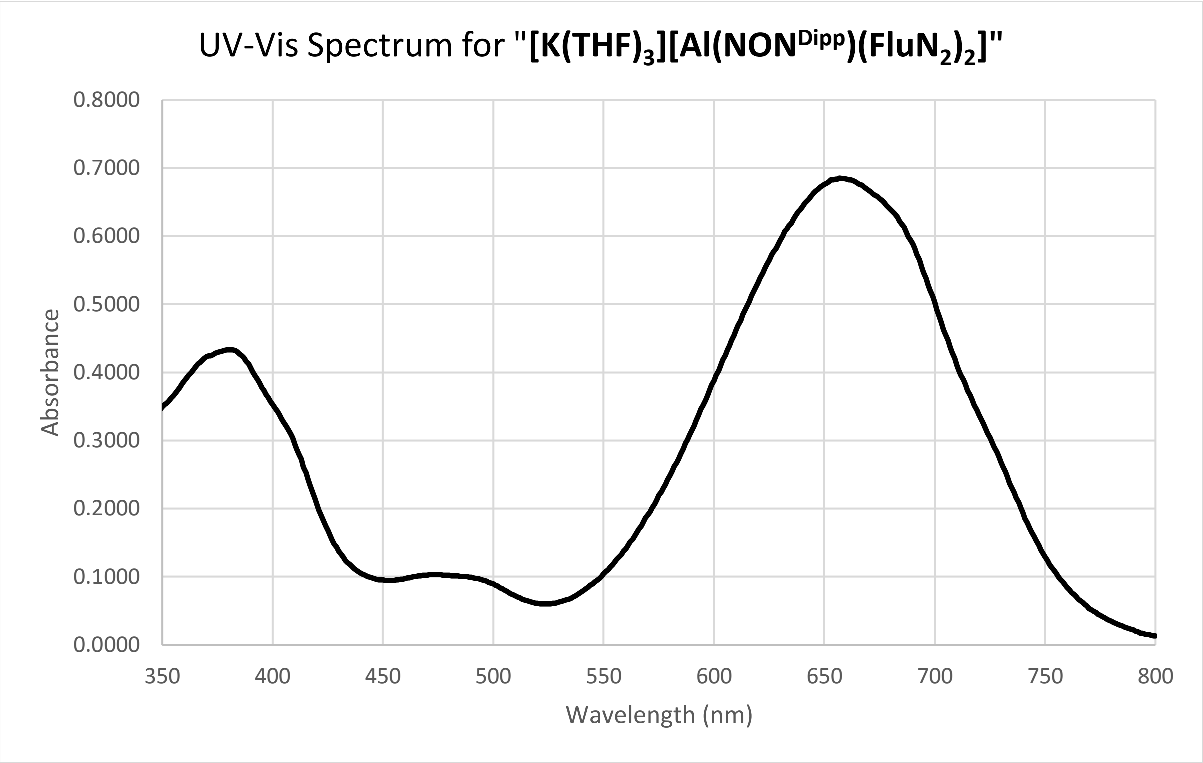

UV-Vis Details		
λ_{max}	Absorbance	ε (L mol^{-1} cm^{-1})
657 nm	0.6849	22321
381 nm	0.4329	14108
Solvent: Toluene<br>Concentration of Sample: 3.07 x 10^{-5} mol L^{-1} (V = 3.60 mL)<br>Observed Colour: Dark Blue (Intense)		

Figure S7 Displacement ellipsoid plot (30 % probability, disordered groups, THF solvate and H-atoms omitted) of the asymmetric unit of $[K(THF)_5][Al(NON)\{(fluN_2)_2\}]$ (**1·THF**)

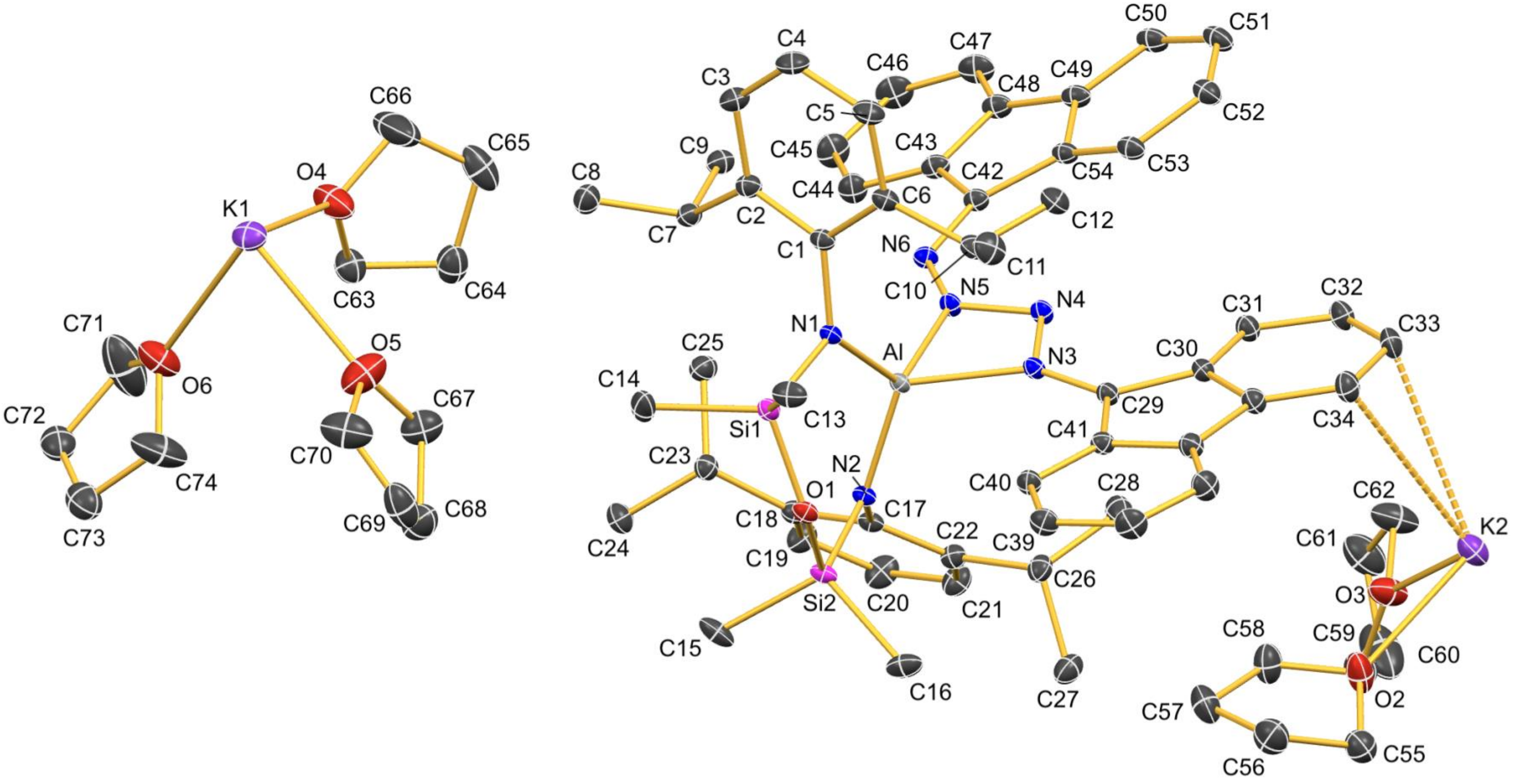

Figure S8 Displacement ellipsoid plot (30 % probability, disordered groups, THF solvate and H-atoms omitted) of the grown fragment of $[K(THF)_5][Al(NON)\{(fluN_2)_2\}]$ (**1·THF**). ' 2–*x*, 2–*y*, –*z*; '' –*x*, 1–*y*, 1–*z*.

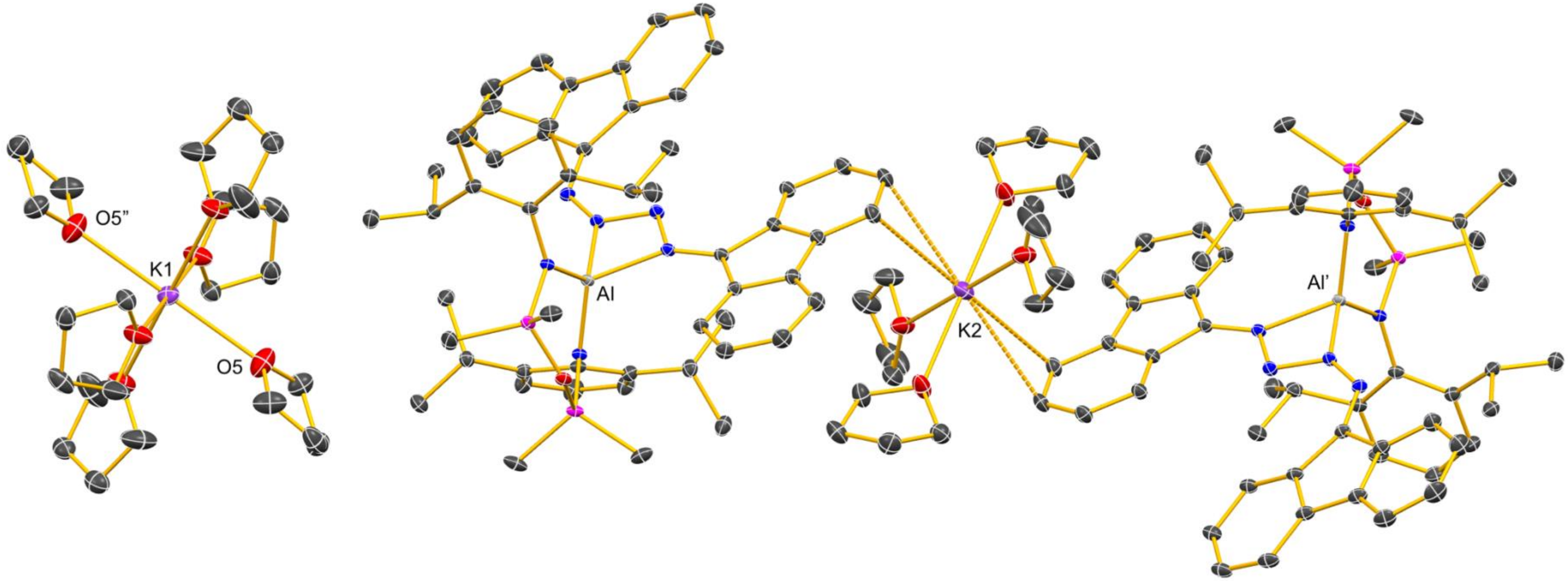

Synthesis of [K(18-c-6)][Al(NON){(fluN$_2$)$_2$}] (1·crown)

9-diazofluorene (47 mg, 0.24 mmol) was suspended in hexane (~1 mL) and added dropwise to a bright yellow solution of K[Al(NON)] (67 mg, 0.12 mmol) in hexane (~1 mL) to give an intense dark blue suspension. The solvent was reduced *in vacuo* and the residue dissolved in THF (~1 mL). A solution of 18-crown-6 (32 mg, 0.12 mmol) in THF (~1 mL) was added to this solution. Crystals were obtained from a THF solution (~2 mL) stored at –30 °C. Yield 60 mg, 41 %.

Anal. Calcd. for $C_{66}H_{86}AlKN_6O_7Si_2 \cdot C_4H_8O$ (*1269.78*)*: C, 66.21; H, 7.46; N, 6.62 %. Found: C, 66.16; H, 7.29; N, 6.07 %. (* = calculated for 1 x THF solvate).

^{1}H NMR (500 MHz, C_6D_6): δ 9.26 (d, J = 7.8, 1H, $C_{13}H_8$), 8.54 (d, J = 8.1, 1H, $C_{13}H_8$), 8.16 (d, J = 7.8, 1H, $C_{13}H_8$), 8.08 – 8.03 (m, 2H, $C_{13}H_8$), 8.00 (d, J = 7.6, 1H, $C_{13}H_8$/C_6H_3), 7.91 (d, J = 7.6, 1H, $C_{13}H_8$), 7.85 (d, J = 7.5, 1H, $C_{13}H_8$), 7.78 – 7.73 (m, 1H, $C_{13}H_8$), 7.48 – 7.42 (m, 2H, $C_{13}H_8$), 7.36 (dt, J = 13.1, 7.4, 3H, $C_{13}H_8$), 7.28 – 7.23 (m, 1H, $C_{13}H_8$), 7.18 (d, J = 7.0, 1H, $C_{13}H_8$), 7.11 (d, J = 7.6, 2H, C_6H_3), 6.87 (d, J = 7.6, 2H, C_6H_3), 6.79 (t, J = 7.6, 2H, C_6H_3), 4.58 (sept, J = 6.8, 2H, C*H*Me$_2$), 4.05 (sept, J = 6.8, 2H, C*H*Me$_2$), 3.64 – 3.51 (m, 6H, THF), 2.56 (s, 24H, crown-C*H*$_2$), 1.98 (d, J = 6.7, 6H, CH*Me*$_2$), 1.66 (d, J = 6.6, 6H, CH*Me*$_2$), 1.52 – 1.35 (m, 6H, THF), 1.01 (d, J = 6.6, 6H, CH*Me*$_2$), 0.71 (d, J = 6.4, 6H, CH*Me*$_2$), 0.59 (s, 6H, Si*Me*$_2$), 0.51 (s, 6H, Si*Me*$_2$).

^{13}C{^{1}H} NMR (126 MHz, C_6D_6): δ 148.3, 147.7, 142.4, 141.0, 137.1, 135.1, 135.0, 134.6, 133.1, 132.6, 130.9, 130.8, 126.2, 125.9, 125.5, 124.9, 124.0, 123.9, 123.6, 123.4, 123.3, 121.2, 120.7, 120.5, 120.3, 120.2, 119.7, 118.5 (*C*$_{13}$H$_8$, *C*$_6$H$_3$), 69.6 (crown-*C*H$_2$), 67.8 (THF), 29.0 (CH*Me*$_2$), 28.6 (*C*HMe$_2$), 27.8 (CH*Me*$_2$), 27.7 (*C*HMe$_2$), 25.8 (THF), 25.2, 24.7 (CH*Me*$_2$), 3.6, 2.9 (Si*Me*$_2$).

Figure S9 ^{1}H NMR Spectrum (500 MHz, C_6D_6) of [K(18-c-6)][Al(NON){(fluN$_2$)$_2$}] (**1·crown**)

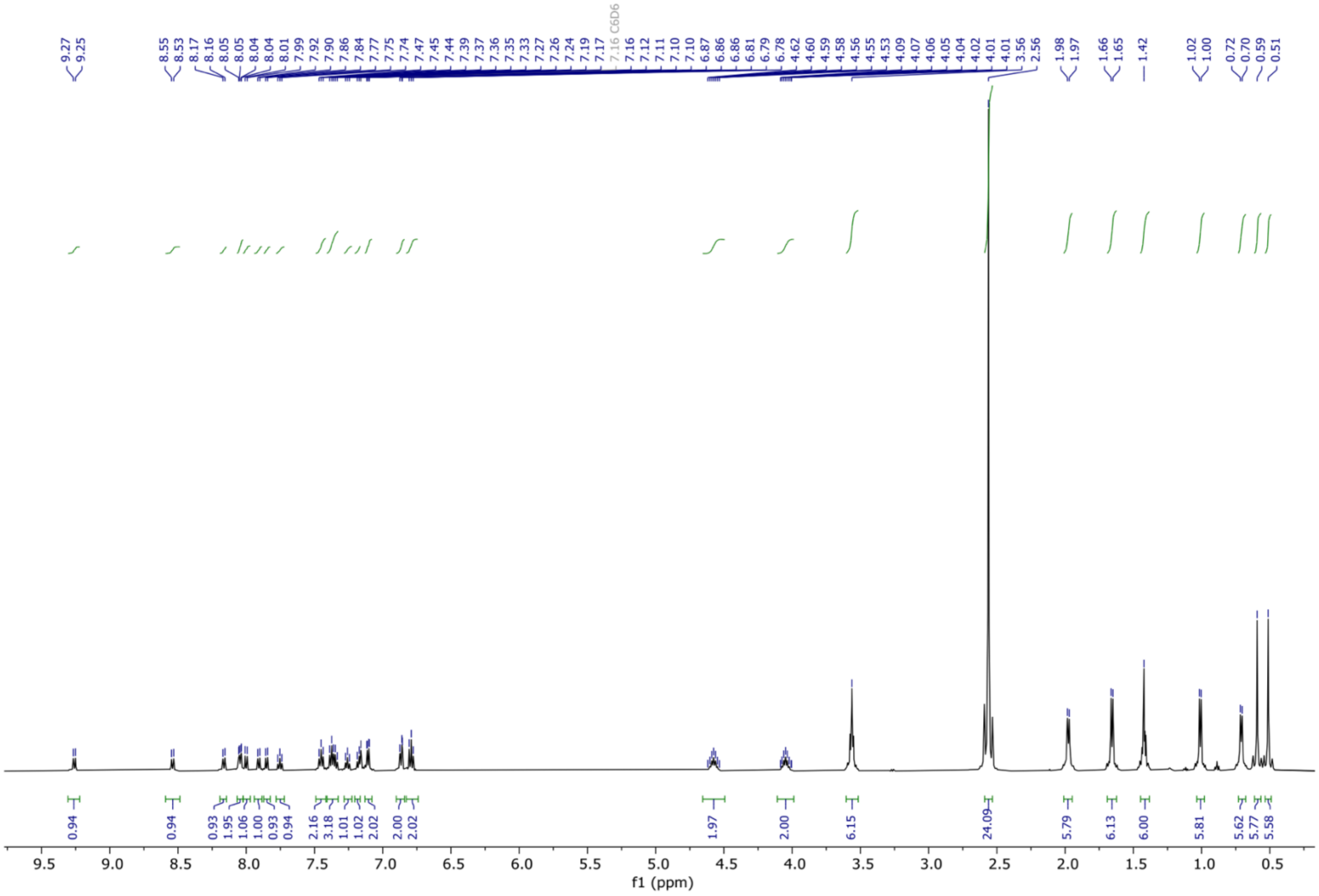

Figure S10 $^{13}C\{^{1}H\}$ NMR Spectrum (126 MHz, C_6D_6) of [K(18-c-6)][Al(NON){(fluN_2)$_2$}] (**1·crown**)

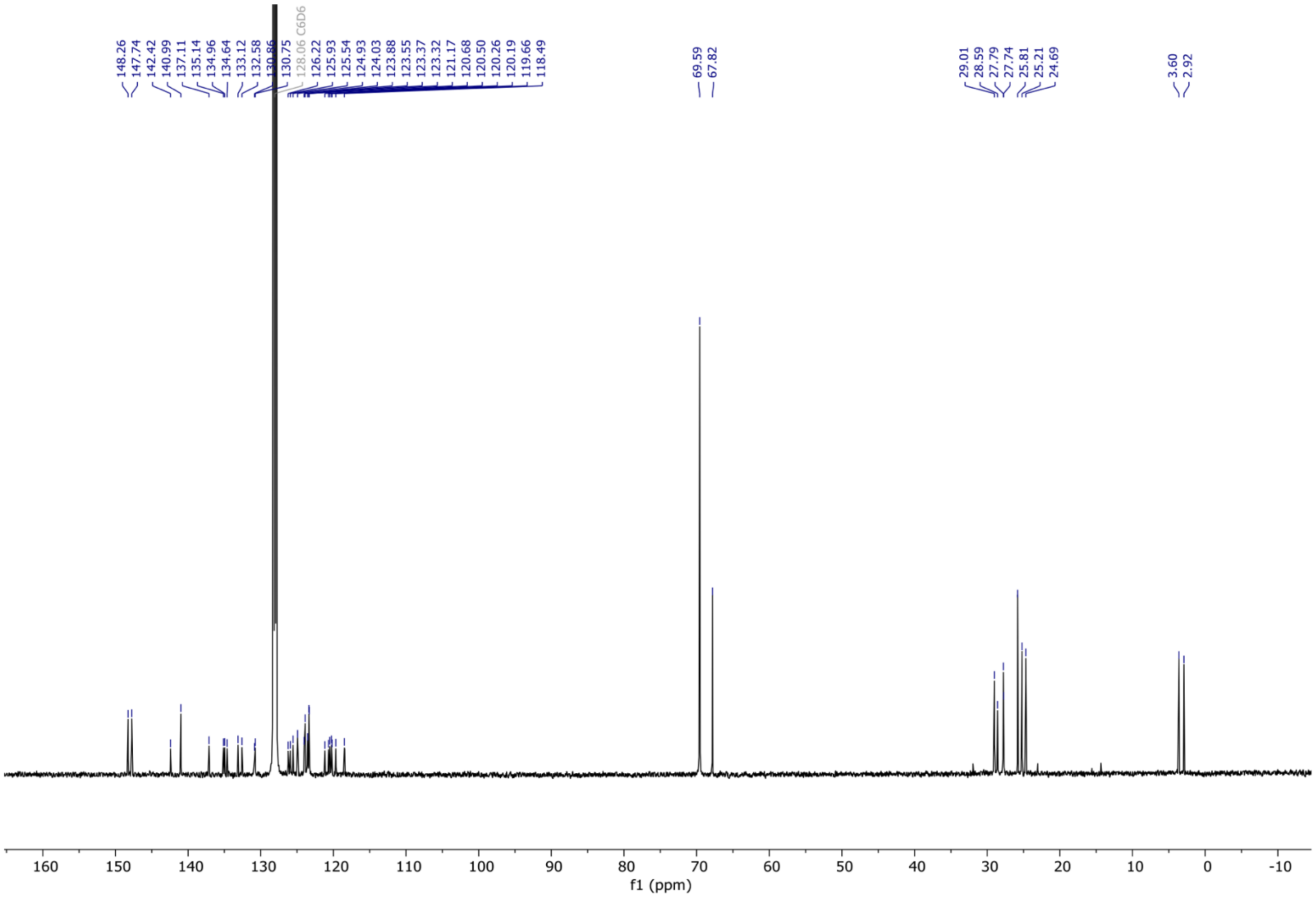

Figure S11 UV/vis Spectrum of [K(18-c-6)][Al(NON){(fluN$_2$)$_2$}] (**1·crown**)

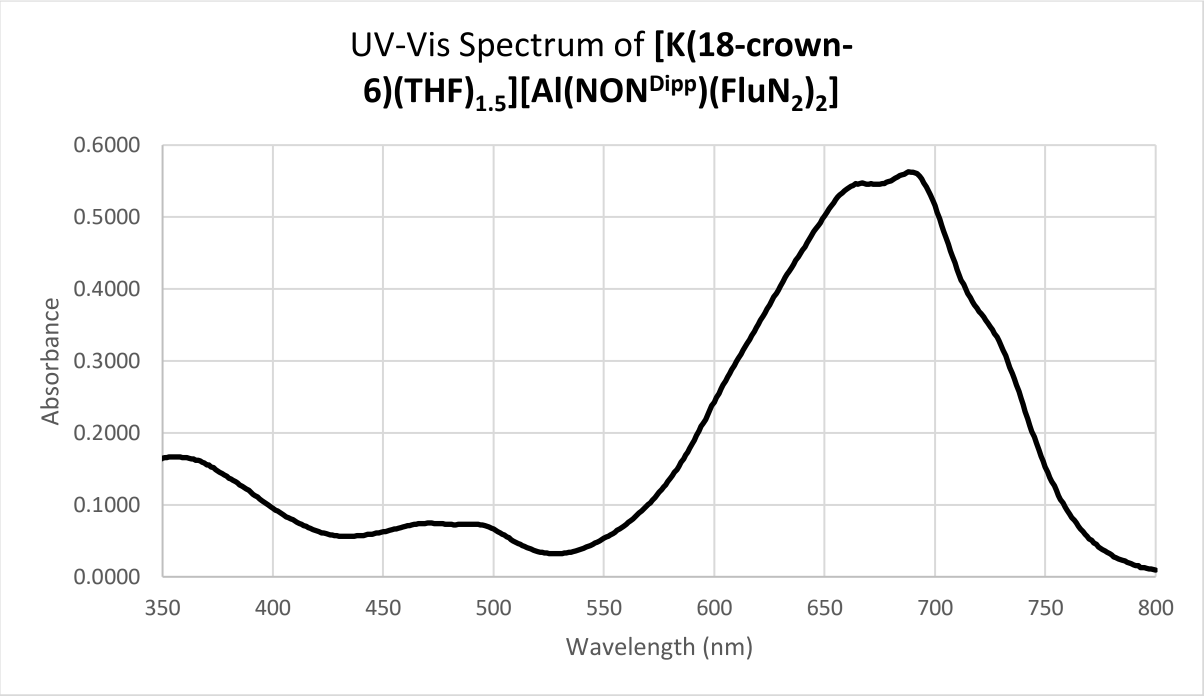

**UV-Vis Details**		
λ_{max}	Absorbance	ε (L mol^{-1} cm^{-1})
688 nm	0.5629	26642
667 nm	0.4329	25913
343 nm	0.1669	0.1669
Solvent: Toluene<br>Concentration of Sample: 2.11 x 10^{-5} mol L^{-1} (V = 3.58 mL)<br>Observed Colour: Dark Blue (Intense)		

Synthesis of [K([2.2.2]crypt)]][Al(NON){(fluN$_2$)$_2$}] (1·crypt)

Procedure A

9-diazofluorene (42 mg, 0.22 mmol) was suspended in hexane (~1 mL) and added dropwise to a bright yellow solution of K[Al(NON)] (60 mg, 0.11 mmol) in hexane (~1 mL) to give an intense dark blue suspension. The solvent was removed *in vacuo* and the residue dissolved in THF (~1 mL). A solution of [2.2.2]cryptand (41 mg, 0.11 mmol) in THF (~1 mL) was added. Crystals were obtained from a THF solution (~2 mL) stored at –30 °C. Yield 110 mg, 76 %.

Attempted analysis by NMR spectroscopy in C_6D_6 was hampered by low solubility, and redissolving the crystalline product in THF-D_8 resulted in decomposition. A small number of crystals precipitated from the C_6D_6 NMR sample and were analysed by X-ray diffraction, confirming formation of [K([2.2.2]crypt)]][Al(NON){(fluN$_2$)$_2$}].

Procedure B

A dark blue powder of **1·THF** (81 mg, 0.06 mmol) and 2.2.2-cryptand (24 mg, 0.06 mmol) were combined in THF (~1 mL). Crystals were obtained from a THF solution (~2 mL) stored at –30 °C. Yield 52 mg, 66 %.

Figure S12 Displacement ellipsoid plot (30 % probability, benzene solvate and H-atoms omitted) of the asymmetric unit of [K([2.2.2]crypt)][Al(NON){$(fluN_2)_2$}] (**1·crypt**).

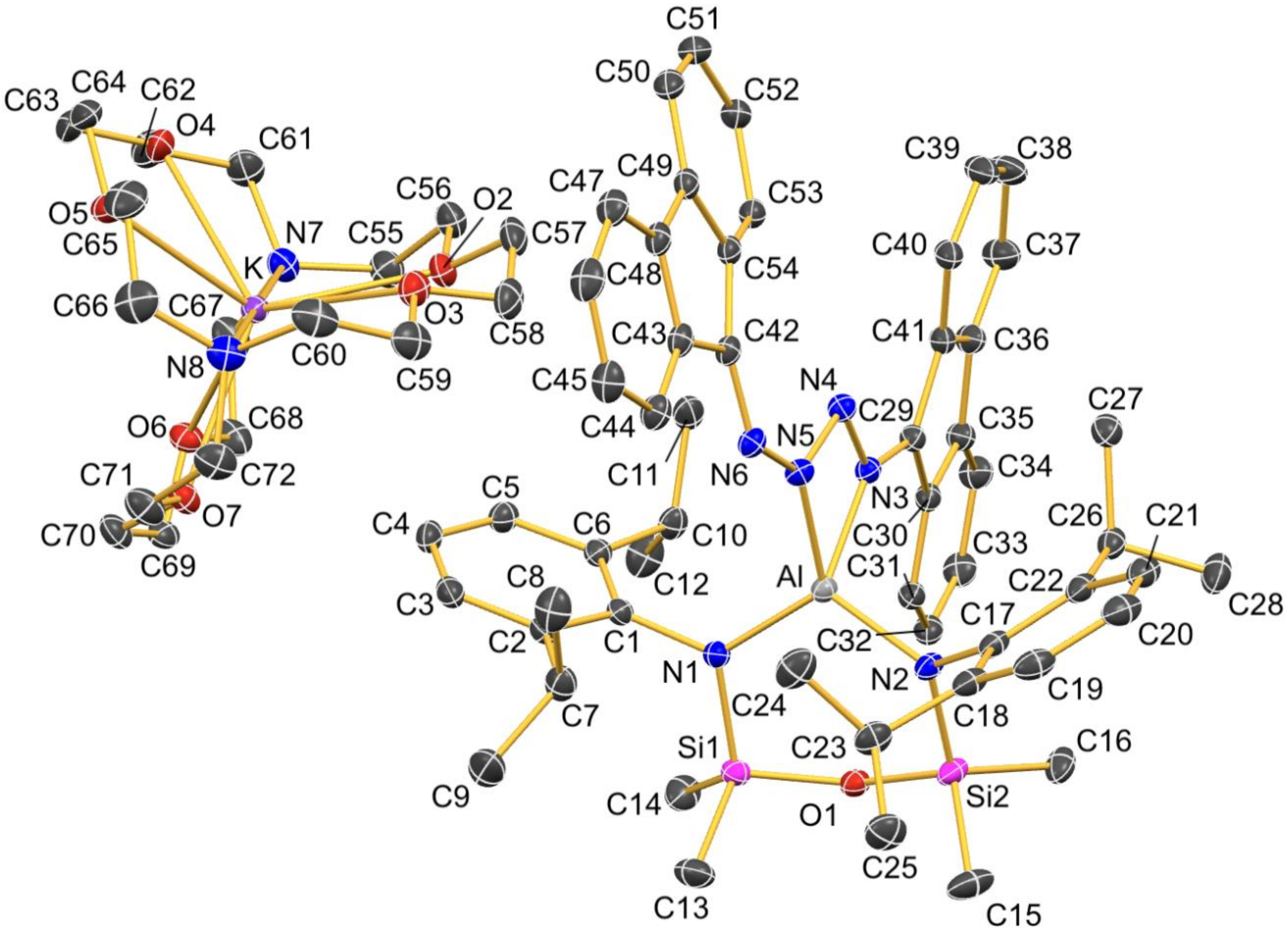

Synthesis of Al(NON)(N=flu)(THF) (2·THF)

A solution of **1·THF** (103 mg, 0.08 mmol) in benzene-d_6 was prepared in a glovebox and transferred to a J. Youngs NMR tube and sealed. The tube was removed from the glovebox and placed in a steel heating block. The solution was heated overnight (*ca.* 18 hours) at 80 °C to give a dark green solution. ^{1}H NMR showed full consumption of the starting material. The NMR tube was taken into a glovebox and the tap removed slowly to relieve pressure (from nitrogen evolution). The contents of the NMR tube were transferred to a scintillation vial and the solvent removed *in vacuo*. The residue was dissolved in toluene (*ca.* 1 mL) to give a dark green solution and a few drops of THF were added. Crystals were obtained from a toluene solution of the reaction mixture stored at -30 °C. Yield 53 mg, 83%.

^{1}H NMR (500 MHz, C_6D_6): δ 7.30 – 7.18 (m, 8H, C_6H_3, $C_{13}H_8$), 7.09 (t, J = 7.4, 2H, $C_{13}H_8$)*, 7.05 – 7.00 (m, 2H, $C_{13}H_8$)*, 6.27 (d, J = 7.4, 2H, $C_{13}H_8$), 3.99 (br sept, 2H, C*H*Me$_2$), 3.87 (s, 2H, THF), 3.81 (br sept, J = 7.2, 2H, C*H*Me$_2$), 3.50 (s, 2H, THF), 1.42 (br d, 6H, CH*Me$_2$*), 1.38 – 1.37 (m, 4H, THF), 1.33 (br d, 6H, CH*Me$_2$*), 1.02 (br d, 6H, CH*Me$_2$*), 0.99 (br d, 6H, CH*Me$_2$*), 0.43 (s, 6H, Si*Me$_2$*), 0.40 (s, 6H, Si*Me$_2$*).

^{13}C{^{1}H} NMR (126 MHz, C_6D_6): δ 166.5, 148.4, 146.3, 144.3, 143.5, 136.8, 130.9, 125.1, 123.8, 123.4, 122.8, 119.5 (C_{13}H$_8$/C_6H$_3$), 72.4, 67.7 (THF), 28.1, 27.7 (*C*HMe$_2$), 27.4†, 25.4, 25.2 (CH*Me$_2$*), 3.7 (Si*Me$_2$*).

* ~1 equivalent of toluene present in sample (indicated by peak at δ 2.11 integrating for 3H), which overlaps with aromatic peaks between δ 7.02 – 7.13.

† Two overlapping resonances appearing as one signal.

We were unable to obtain accurate elemental analysis for this compound.

Figure S13 ^{1}H NMR Spectrum (500 MHz, C_6D_6) of Al(NON)(N=flu)(THF) (**2·THF**)

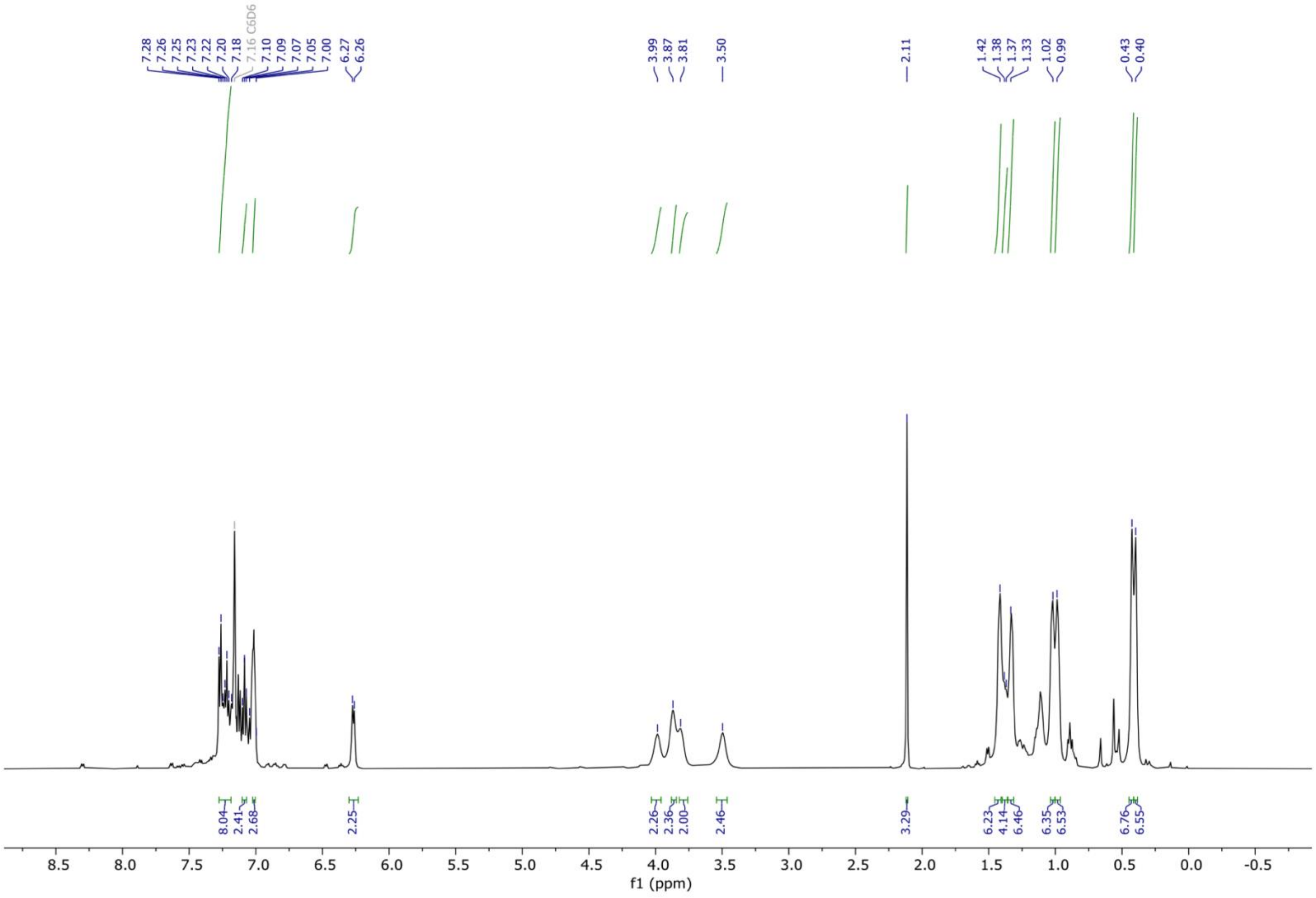

Figure S14 $^{13}C\{^{1}H\}$ NMR Spectrum (500 MHz, C_6D_6) of Al(NON)(N=flu)(THF) (**2·THF**)

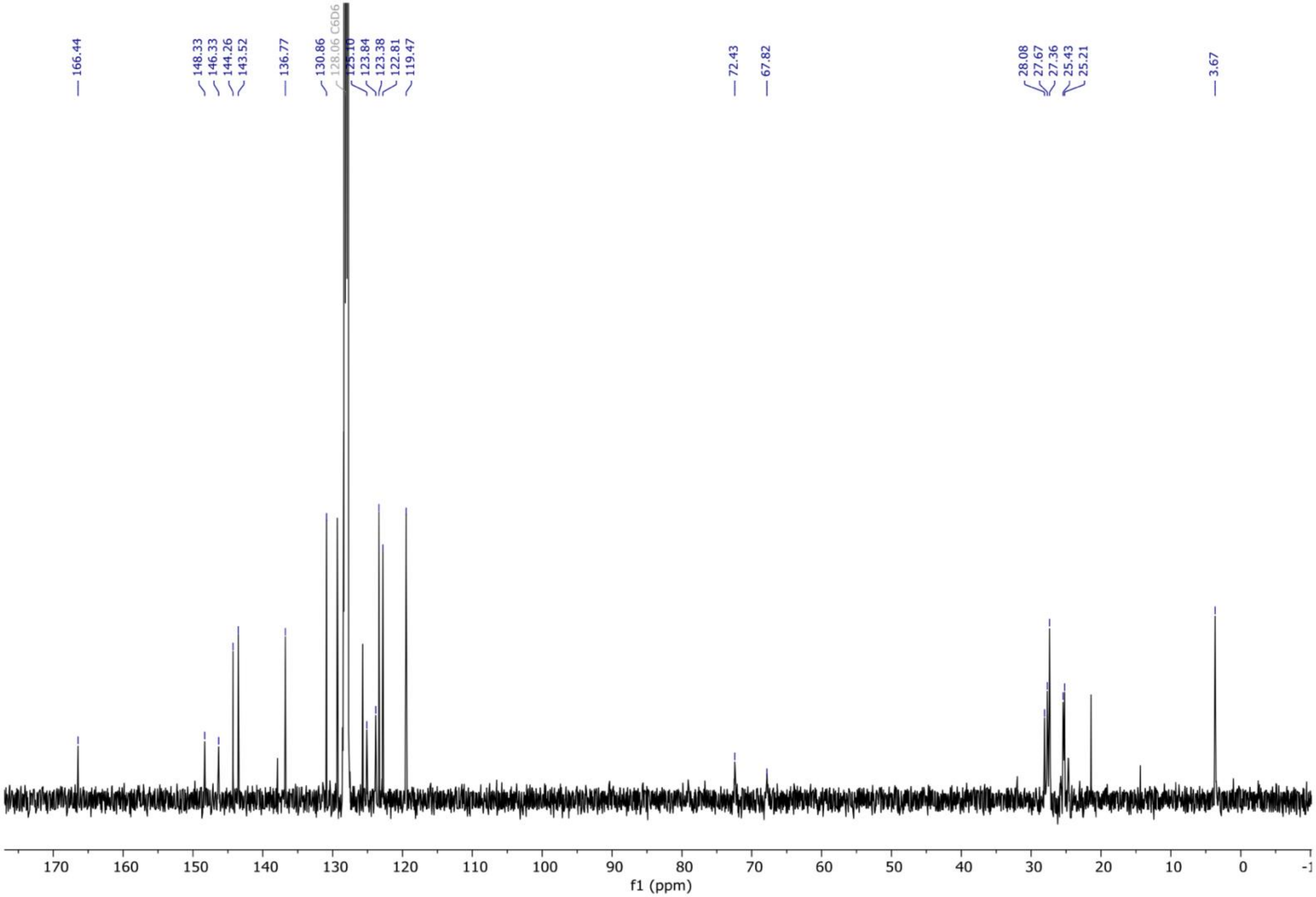

Figure S15 Variable Temperature ^{1}H NMR Spectrum (500 MHz, C_6D_6) of Al(NON)(N=flu)(THF) (**2·THF**)

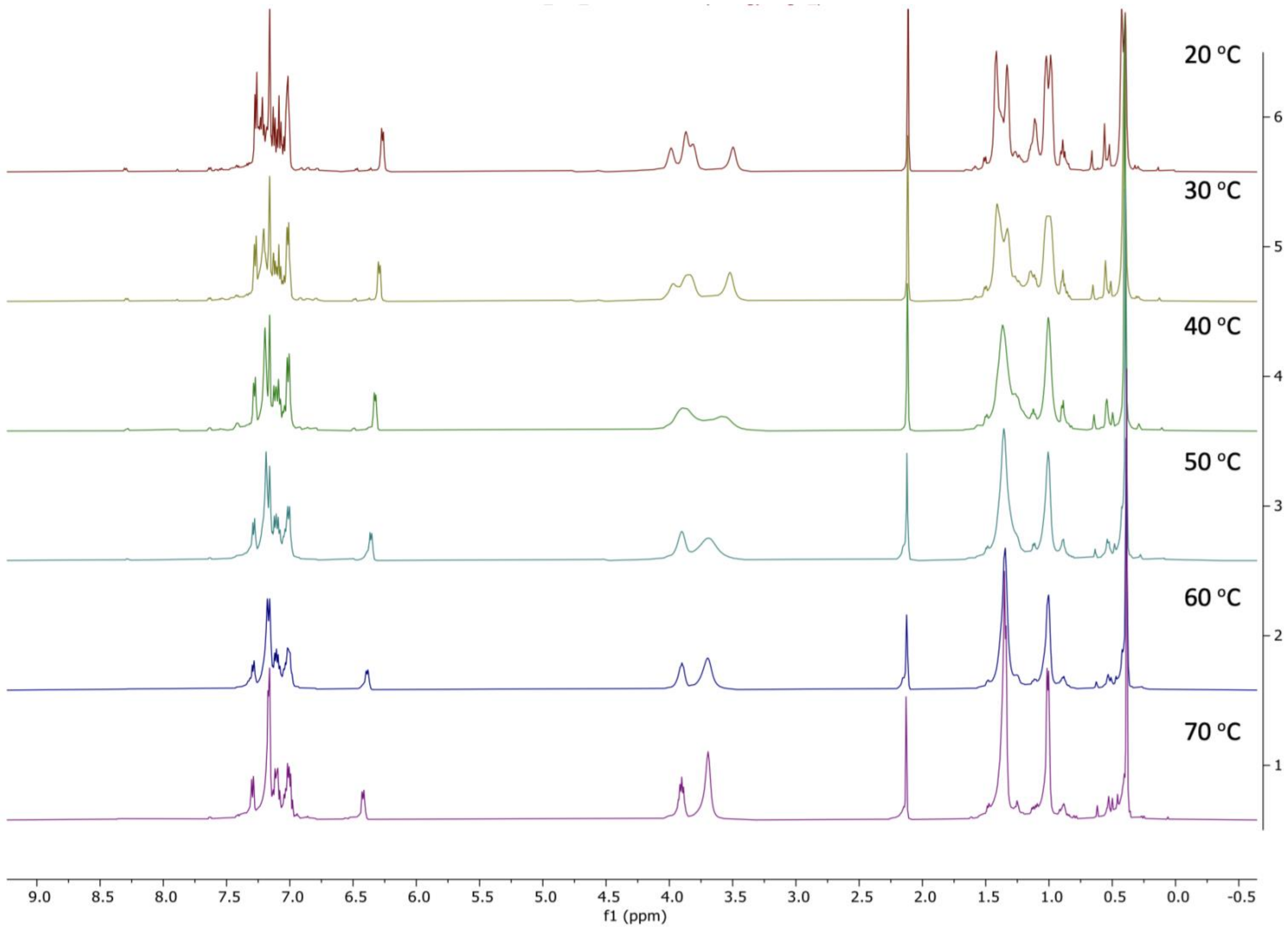

Figure S16 UV/vis Spectrum of Al(NON)(N=flu)(THF) (**2·THF**)

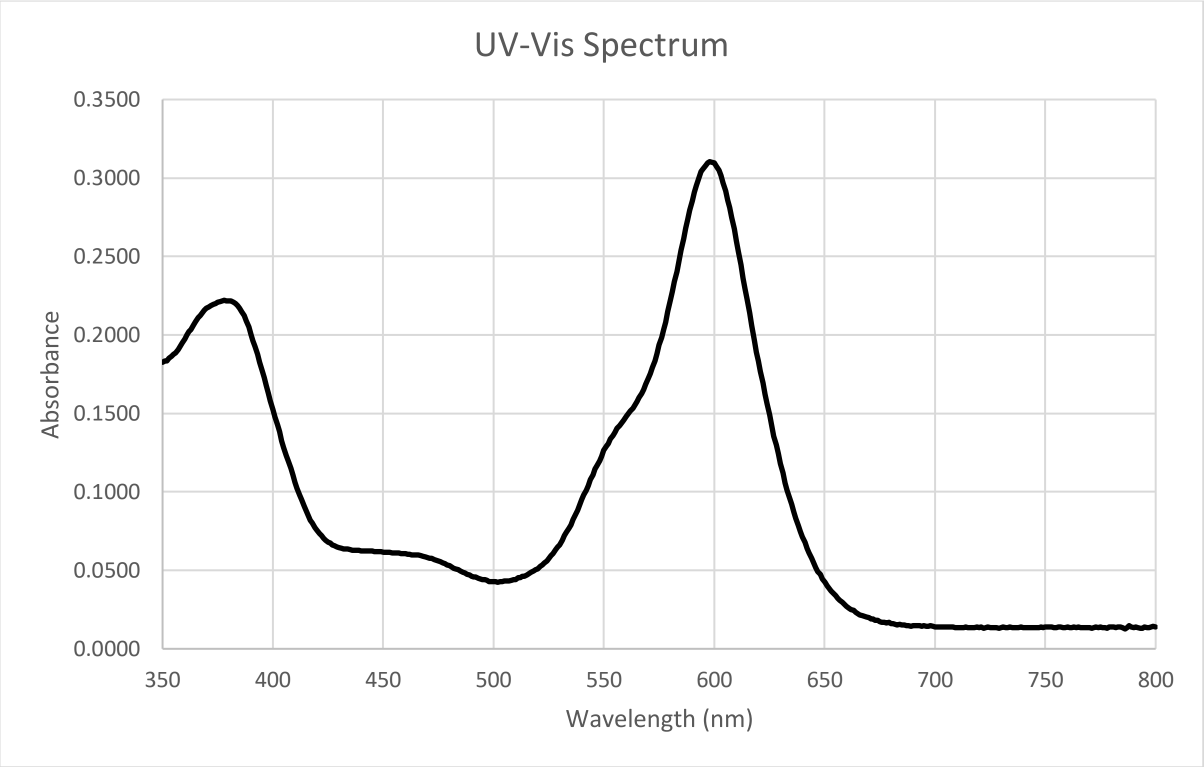

**UV-Vis Details**		
λ_{max}	Absorbance	ε (L mol^{-1} cm^{-1})
598 nm	0.3104	7270
380 nm	0.2219	5196
Solvent: Toluene<br>Concentration of Sample: 3.78 x 10^{-3} mol L^{-1} (V = 3.65 mL)<br>Observed Colour: Dark Blue		

Figure S17 Displacement ellipsoid plot (30 % probability, THF solvate and H-atoms omitted) of the asymmetric unit of Al(NON)(N=flu)(THF) (**2·THF{THF}**).

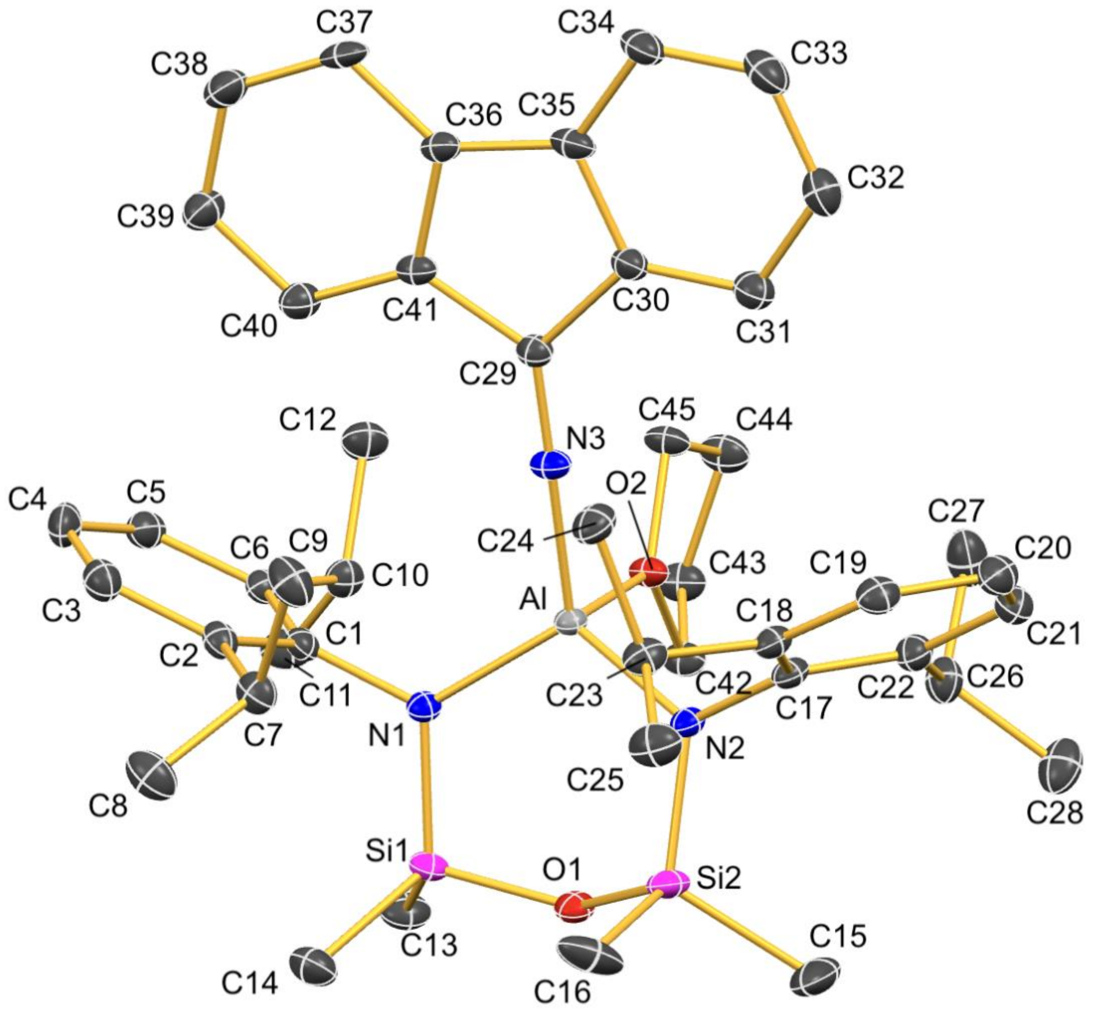

Figure S18 Displacement ellipsoid plot (30 % probability, benzene, disordered toluene solvate and H-atoms omitted) of the asymmetric unit of Al(NON)(N=flu)(THF) (**2·THF{Ar}**).

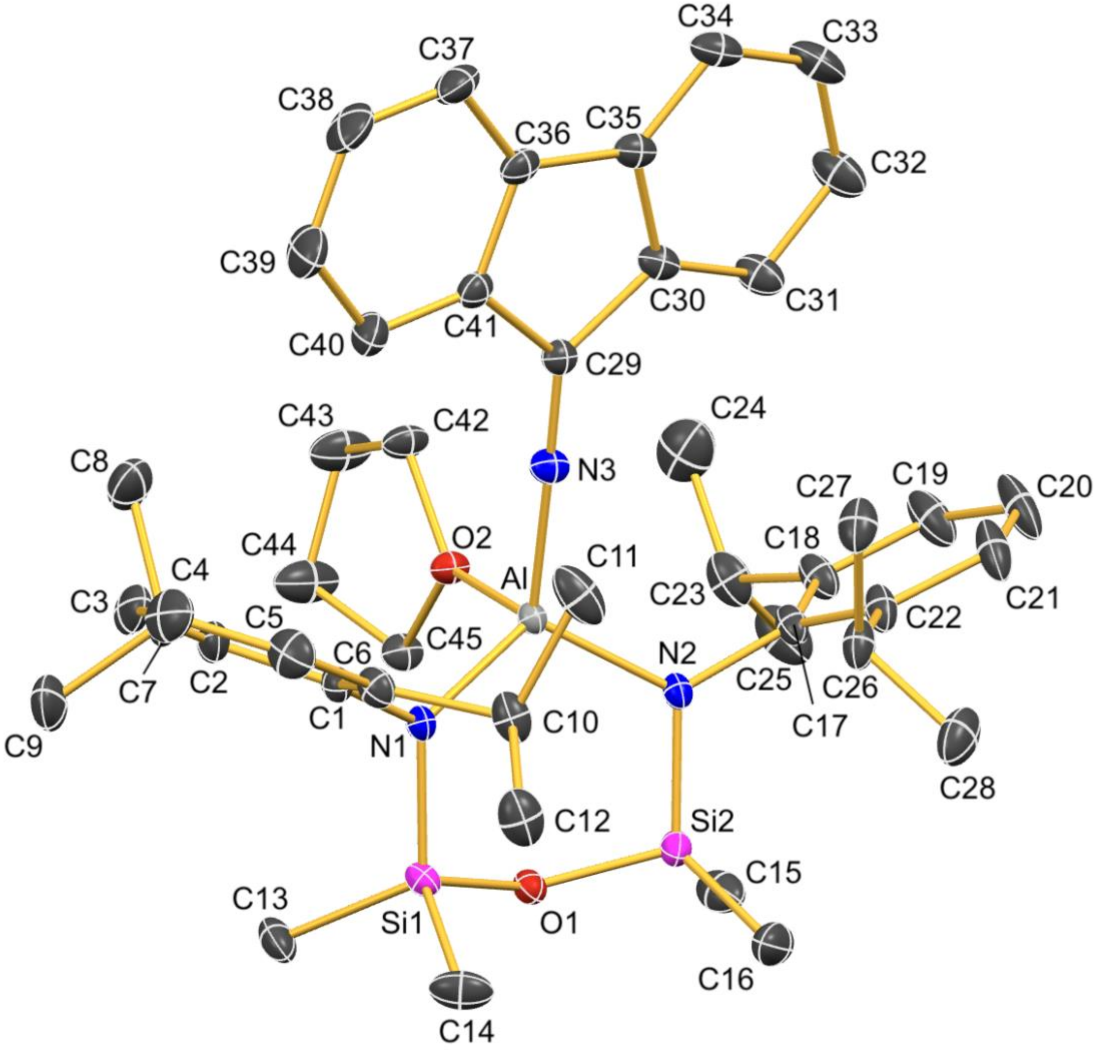

Synthesis of Al(NON)(N=flu)(DMAP) (2·DMAP)

A solution of **1·THF** (56 mg, 0.04 mmol) in benzene-d_6 was prepared in a glovebox and transferred to a J. Youngs NMR tube and sealed. The tube was removed from the glovebox and placed in a steel heating block. The solution was heated overnight (*ca.* 18 hours) at 80 °C to give a dark green solution. ^{1}H NMR spectrum showed full consumption of the starting material. The NMR tube was taken into a glovebox and the tap removed slowly to relieve pressure (from nitrogen evolution). DMAP (5 mg, 0.04 mmol) was added to the reaction mixture and the NMR tube sealed and mixed. The contents of the NMR tube were then transferred to a scintillation vial and the solvent removed *in vacuo*. The residue was dissolved in hexane (*ca.* 1 mL) to give a dark green solution. Crystals were obtained from a hexane solution of the reaction mixture stored at room temperature. Yield 30 mg.

Analysis by NMR showed formation of an inseparable mixture of unidentified products, from which single crystals of Al(NON)(N=flu)(DMAP) were separated and analysed by X-ray diffraction.

Figure S19 Series of ^{1}H NMR Spectra (500 MHz, C_6D_6) charting the conversion of **1·THF** to **2·THF** to **2·DMAP**.

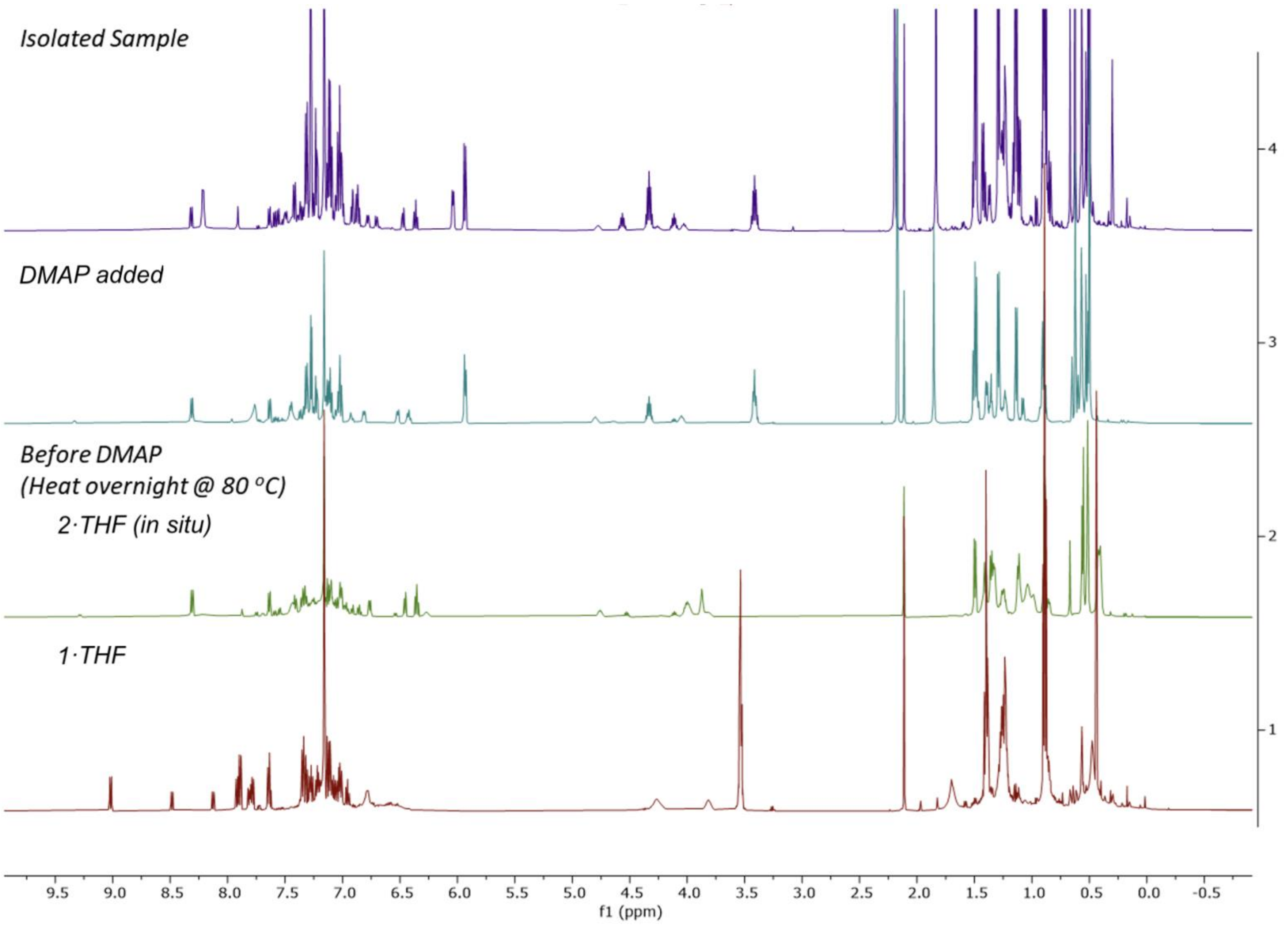

Figure S20 Displacement ellipsoid plot (30 % probability, H-atoms omitted) of the asymmetric unit of Al(NON)(N=flu)(DMAP) (**2·DMAP**).

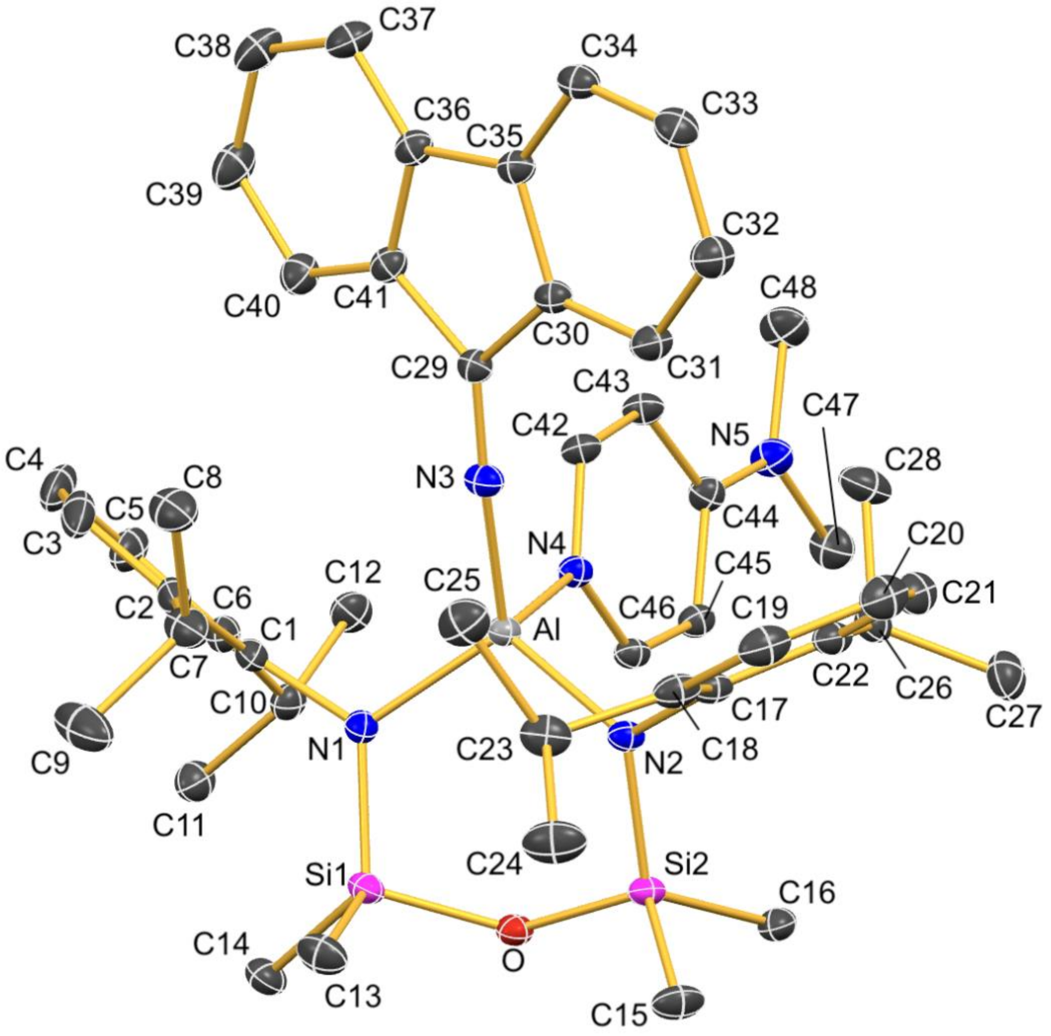

Synthesis of $[K_2(THF)_3][(fluN)_2]$ (3)

9-diazofluorene (63 mg, 0.33 mmol) and K[Al(NON)] (91 mg, 0.17 mmol) were suspended in C_6D_6 (~0.6 mL) to give an intense dark blue solution. The solution was heated overnight (*ca.* 18 hours) at 80 °C to give a dark green solution. The NMR tube was taken into a glovebox and the tap removed slowly to relieve pressure (from nitrogen evolution). DMAP (5 mg, 0.04 mmol) was added to the reaction mixture and the NMR tube sealed and mixed. The contents of the NMR tube were then transferred to a scintillation vial and the solvent removed *in vacuo*. The residue was washed with hexane (5 x 2 mL) and a toluene/THF mixture (1 mL, 1:1) was added. A small quantity of crystals of $[K_2(THF)_3][(fluN)_2]$ (**3**) that co-crystallized with **2·THF** were separated and characterised by single crystal X-ray diffraction. It was not possible to obtain any additional analysis on these crystals.

Figure S21 Displacement ellipsoid plot (20 % probability, disordered- and H-atoms omitted) of the asymmetric unit of $[K_2(THF)_3][(fluN)_2]$ (**3**).

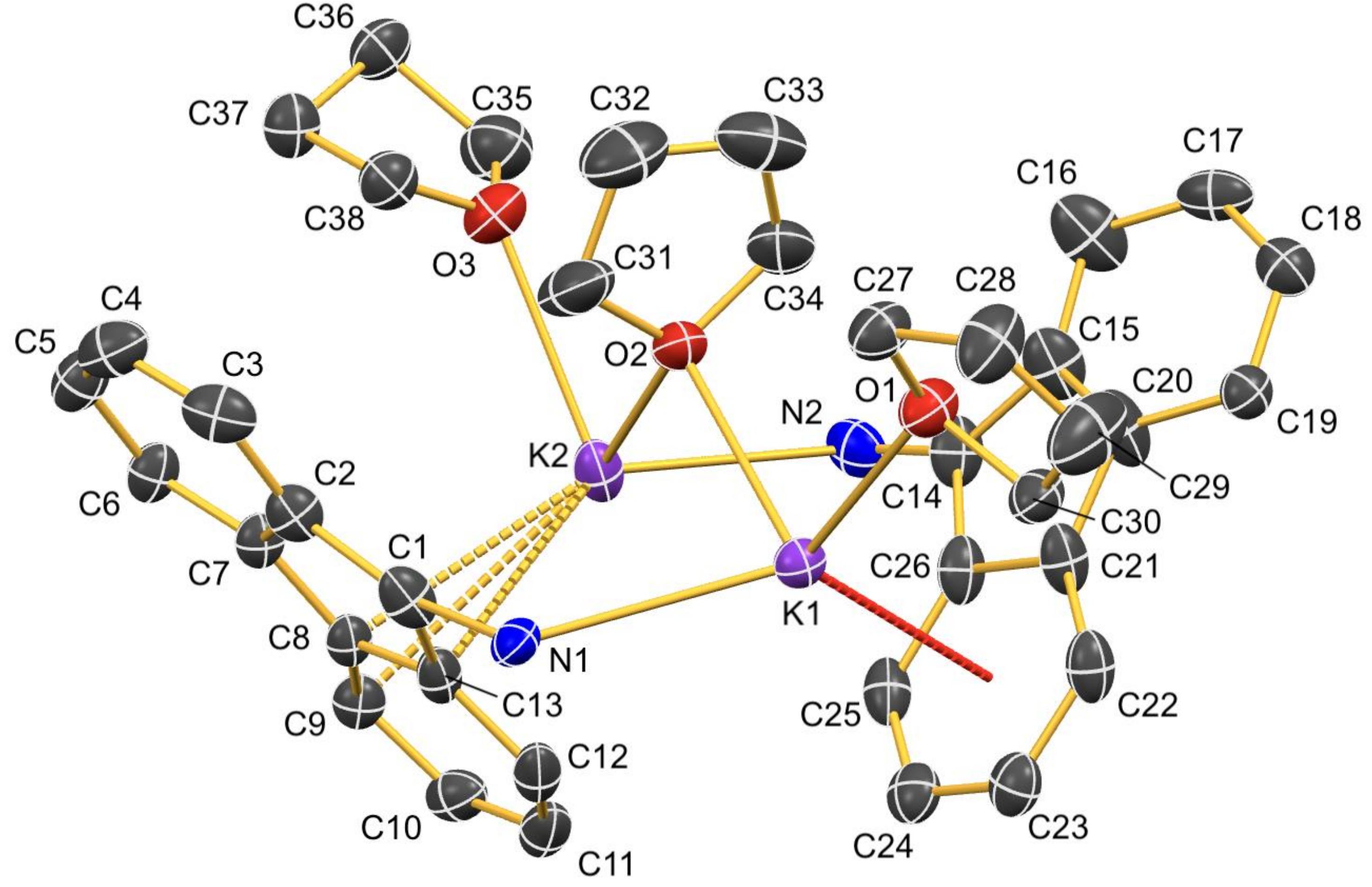

Synthesis of Al(NON){*i*PrNC(N=CMe$_2$)NC(H)flu)} (4)

A solution of **1·THF** (83 mg, 0.07 mmol) in benzene-D$_6$ was transferred to J Youngs NMR tube and heated to 80 ℃ for 18 hours. The pressure of the NMR tube from liberation of nitrogen was relieved in a nitrogen-filled glovebox, and a solution of *N,N'*-di*iso*propylcarbodiimide (9 mg, 0.07 mmol) was added. Reaction progress was monitored by ^{1}H NMR spectroscopy and observed to be complete within 15 minutes of addition. The solution was transferred to a scintillation vial and solvent reduced *in vacuo* to give dark green residue. The residue was washed with hexane (3 times, 5 mL) and then dried. Toluene (~2 mL) was then added to the residue and a few drops of THF to ensure complete dissolution. Storage of the toluene/THF mixture at –30 ℃ yielded dark blue crystals suitable for single crystal X-ray diffraction experiments. Yield 17 mg, 31 %.

^{1}H NMR (500 MHz, C$_6$D$_6$): δ 7.34 (dd, J = 6.8, 3.0, 2H, C$_6$$H_3$), 7.30 – 7.24 (m, 4H, C$_6$$H_3$), 7.18 (d, J = 7.8, 2H, C$_{13}$$H_8$), 6.98 (t, J = 7.4, 2H, C$_{13}$$H_8$), 6.92 (t, J = 7.4, 2H, C$_{13}$$H_8$), 5.83 (d, J = 7.4, 2H, C$_{13}$$H_8$), 5.07 (s, 1H, Flu-C*H*), 4.29 (sept, J = 6.8, 2H, C*H*Me$_2$), 4.11 (sept, J = 6.8, 2H, C*H*Me$_2$), 3.53 (s, 6H, THF), 3.37 (sept, J = 6.6, 1H, C*H*Me$_2$), 1.50 (d, J = 6.8, 6H, CHMe_2), 1.45 (d, J = 6.8, 6H, CHMe_2), 1.40 (s, 6H, THF), 1.29 – 1.23 (m, 18H, CHMe_2), 0.59 (s, 6H, NCMe_2), 0.54 (s, 6H, SiMe$_2$), 0.38 (s, 6H, SiMe_2).

^{13}C{^{1}H} NMR (126 MHz, C$_6$D$_6$): δ 170.3 (N*C*N), 169.8 (N*C*Me$_2$), 147.5, 147.0, 146.3, 143.5, 139.7, 129.3, 128.6, 127.8*, 127.5, 126.7, 125.0, 124.7, 123.4, 119.0 (C_6H$_3$, C_{13}H$_8$), 67.8 (THF), 59.5 (Flu-*C*H), 45.5 (*C*HMe$_2$), 28.5 (*C*HMe$_2$), 28.4 (CHMe_2), 27.7 (*C*HMe$_2$), 27.6 (CHMe_2), 26.2, 26.1 (CHMe_2), 25.8 (THF), 25.7 (*C*Me$_2$), 24.8 (CHMe_2), 4.7, 3.2 (SiMe_2).

* Quaternary ^{13}C signal overlapping with residual solvent peak.

We were unable to obtain accurate elemental analysis for this compound.

Figure S22 ^{1}H NMR Spectrum (500 MHz, C_6D_6) of Al(NON){*i*PrNC(N=CMe_2)NC(H)flu)} (**4**)

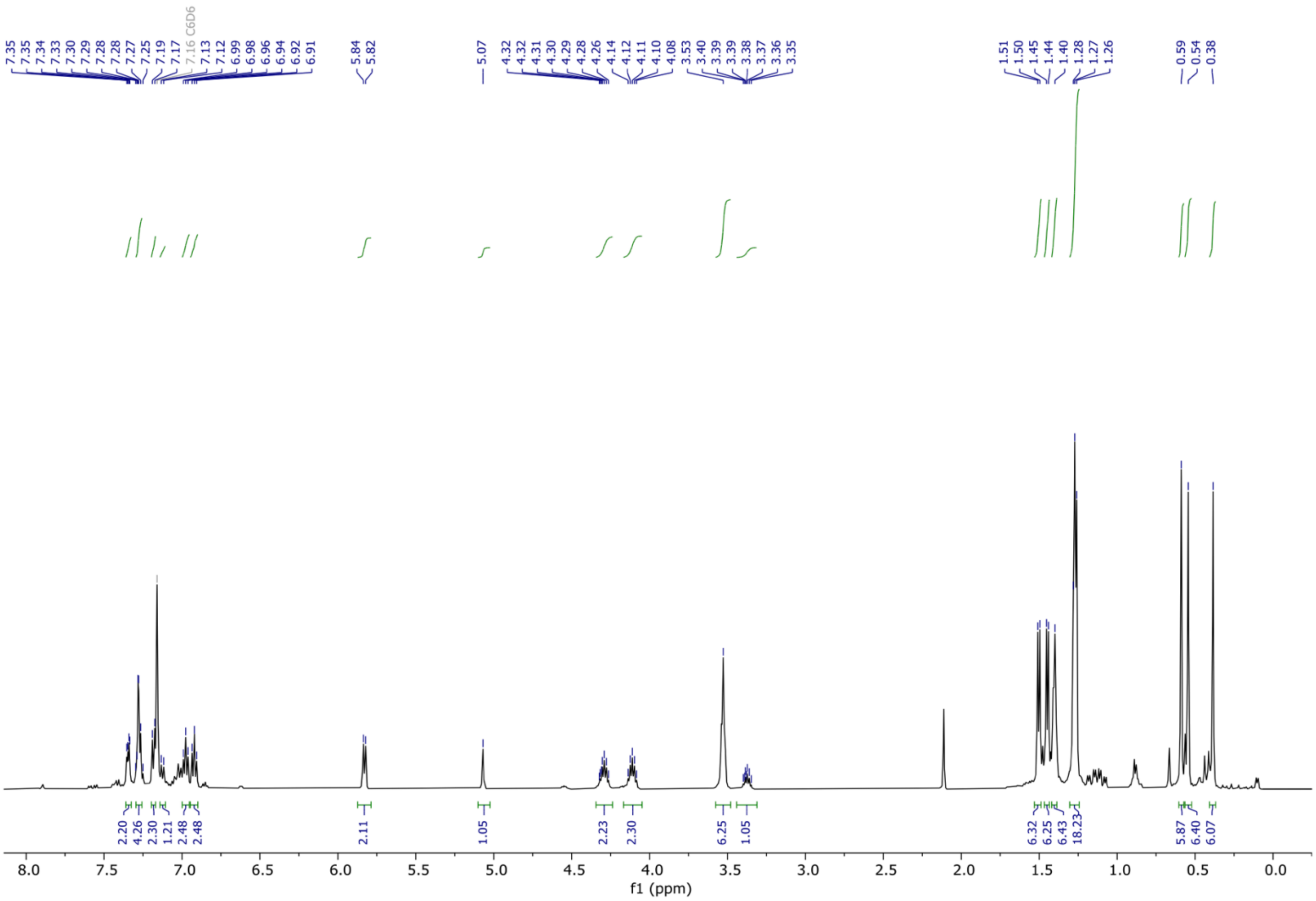

Figure S23 ^{1}H NMR Spectrum (126 MHz, C_6D_6) of Al(NON){*i*PrNC(N=CMe$_2$)NC(H)flu)} (**4**)

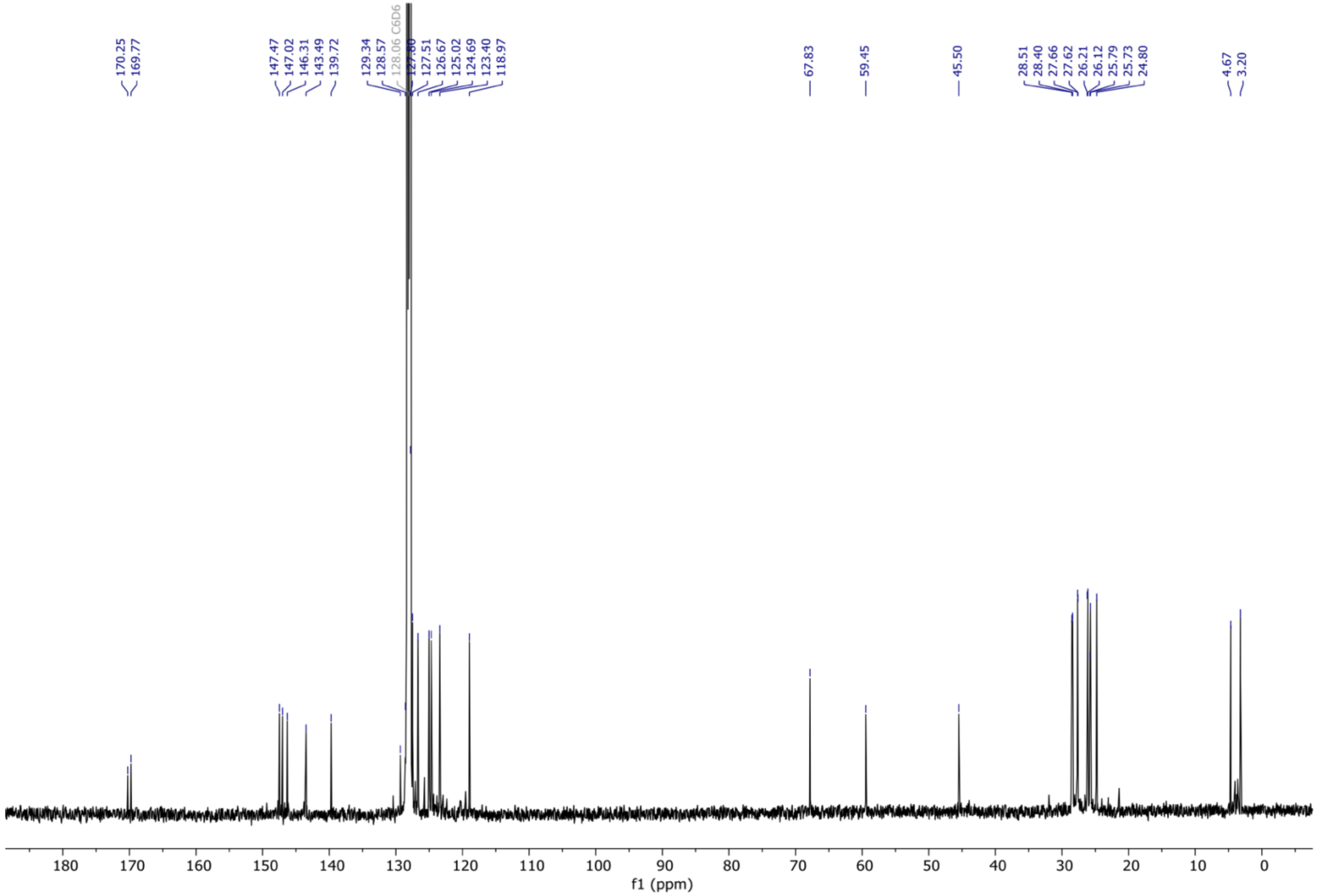

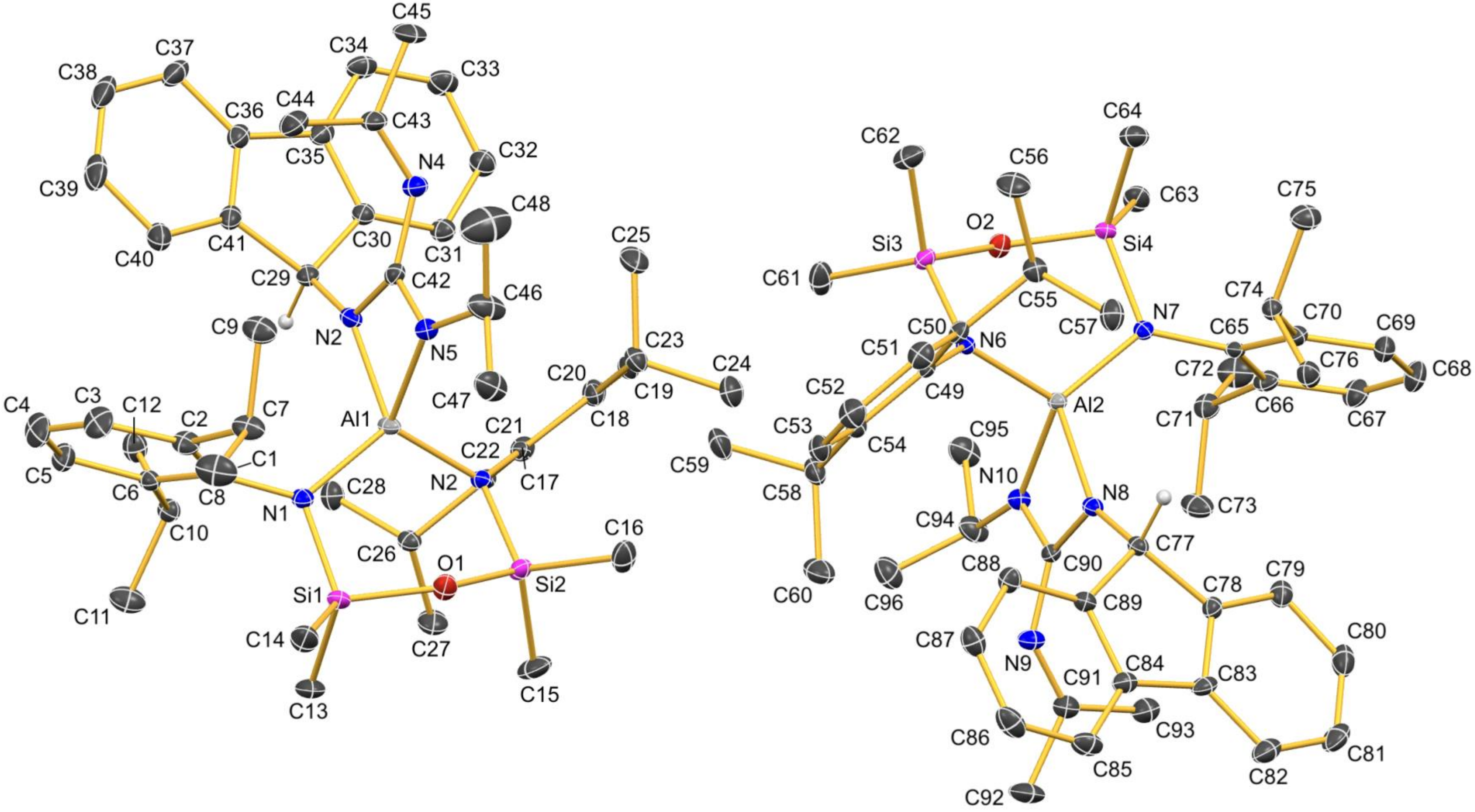

Figure S24 Displacement ellipsoid plot (30 % probability, H-atoms omitted) of the asymmetric unit of Al(NON){*i*PrNC(N=CMe_2)NC(H)flu)} (**4**).

Crystallographic Details

Crystals were covered in inert oil and suitable single crystals were selected under a microscope and mounted on an Agilent SuperNova diffractometer fitted with an EOS S2 detector. Data were collected at 120 K (unless indicated otherwise) using focused microsource Cu Kα radiation at 1.54184 Å. Intensities were corrected for Lorentz and polarisation effects and for absorption using multi-scan methods.[40] Space groups were determined from systematic absences and checked for higher symmetry. All structures were solved using direct methods with SHELXS,[41] refined on F^2 using all data by full matrix least-squares procedures with SHELXL-97,[42] within the WinGX[43] program. Non-hydrogen atoms were refined with anisotropic displacement parameters. Hydrogen atoms were placed in calculated positions or manually assigned from residual electron density where appropriate, unless otherwise stated. The functions minimized were Σ*w*(*F2*o-*F2c*), with *w* = [σ2(F2o) + *a*P2 + *bP*]-1, where *P* = [max(Fo)2 + 2F2c]/3. The isotropic displacement parameters are 1.2 or 1.5 times the isotropic equivalent of their carrier atoms.

Additional Details:

K[Al(NON){(fluN$_2$)$_2$}] (1): A poorly resolved toluene was present on a 3-fold rotation access. This was treated as a diffuse contribution to the overall scattering without specific atom positions by SQUEEZE/PLATON. Details provided in .cif. The data was also refined as a TWIN with BASF = 0.298.

[K(THF)$_5$][Al(NON){(fluN$_2$)$_2$}] (1·THF): Components of the K-bound THFs are disordered (1 x CH_2 in $[K(THF)_6]^+$ and OCH_2CH_2 in bridging $[K(THF)_4]^+$ unit) and were modelled over two positions. The unit cell contains one well defined and one poorly defined THF solvate molecule. The latter was treated as a diffuse contribution to the overall scattering without specific atom positions by SQUEEZE/PLATON. Details provided in .cif.

Al(NON)(N=flu)(THF)·THF (2·THF{THF}): The data was refined as a TWIN with BASF = 0.445.

Al(NON)(N=flu)(THF)·(benzene)(toluene) (2·THF{Ar}): The toluene and benzene solvates were each located on an inversion centre. The former was refined as a full molecule at 0.5 occupancy.

[K$_2$(THF)$_3$][(fluN)$_2$] (3): There is considerable disorder in this structure involving the coordinated THF molecules, which extends into the position carbon atoms of one of the fluorenyl groups. There is also a disorder of the nitrogen atoms in one of the $[fluN{=}N\text{-}flu]^{2-}$ dianions.

Table S1 Crystal structure and refinement data for K[Al(NON){(fluN$_2$)$_2$}] (**1**), [K(THF)$_5$][Al(NON){(fluN$_2$)$_2$}] (**1·THF**) and [K([2.2.2]crypt)][Al(NON){(fluN$_2$)$_2$}] (**1·crypt**).

	**1**	**1·THF**	**1·crypt**
**Empirical formula**	$C_{56.33}H_{64.67}AlKN_6OSi_2$	$C_{82}H_{118}AlKN_6O_8Si_2$	$C_{81}H_{107}AlKN_8O_7Si_2$
**CCDC Number**	2292504	2292505	2292506
M_r	964.06	1438.08	1427.01
T **[K]**	120.0(1)	120.0(1)	120.0(1)
**Crystal size [mm]**	0.28 × 0.03 × 0.03	0.30 × 0.13 × 0.09	0.25 × 0.09 × 0.06
**Crystal system**	Trigonal	Triclinic	Monoclinic
**Space group**	*R*3 (No. 146)	$P\overline{1}$ (No. 2)	*C*2/c (No. 15)
a **[Å]**	32.8583(5)	13.7759(3)	44.2766(6)
b **[Å]**	32.8583(5)	14.0518(3)	12.7613(2)
c **[Å]**	12.9126(2)	24.3974(5)	28.8937(5)
α **[°]**	90	102.6983(18)	90
β **[°]**	90	91.2559(16)	103.8795(15)
γ **[°]**	120	118.066(2)	90
V **[Å^3]**	12073.5(3)	4022.17(14)	15849.0(4)
Z	9	2	8
$D_{calc.}$ **[mg m^{-3}]**	1.193	1.187	1.196
**Absorption coefficient [mm^{-1}]**	1.79	1.42	1.43
θ **range for data collection [°]**	3.759 to 73.523	3.675 to 73.538	3.613 to 73.676
**Reflections collected**	41005	58892	54075
**Independent reflections**	9918 [R_{int} 0.068]	16049 [R_{int} 0.025]	15825 [R_{int} 0.023]
**Reflections with** $I > 2\sigma(I)$	8618	14067	14046
**Data/restraints/parameters**	9918 / 1 / 599	16049 / 0 / 909	15825 / 0 / 914
**Final** ***R*** **indices** [$I > 2\sigma(I)$]	R_1 = 0.043, wR_2 = 0.093	R_1 = 0.042, wR_2 = 0.108	R_1 = 0.037, wR_2 = 0.099
**Final** ***R*** **indices (all data)**	R_1 = 0.053, wR_2 = 0.098	R_1 = 0.048, wR_2 = 0.112	R_1 = 0.042, wR_2 = 0.104
**GOOF on** F^2	0.98	1.013	1.022
**Largest diff. peak/hole [e.Å^{-3}]**	0.19 and –0.24	0.84 and –0.49	0.26 and –0.26

Table S2 Crystal structure and refinement data for Al(NON)(N=flu)(THF)·THF (**2·THF{THF}**), Al(NON)(N=flu)(THF)·(benzene)(toluene) (**2·THF{Ar}**) and Al(NON)(N=flu)(DMAP) (**2·DMAP**).

	**2·THF{THF}**	**2·THF{Ar}**	**2·DMAP**
**Empirical formula**	$C_{49}H_{70}AlN_3O_3Si_2$	$C_{103}H_{138}Al_2N_6O_4Si_4$	$C_{48}H_{64}AlN_5OSi_2$
**CCDC Number**	2292507	2292508	2292509
M_r	832.24	1690.51	810.2
***T* [K]**	120.0(1)	120.0(1)	120.0(1)
**Crystal size [mm]**	0.25 × 0.04 × 0.03	0.30 × 0.13 × 0.09	0.16 × 0.12 × 0.05
**Crystal system**	Monoclinic	Triclinic	Triclinic
**Space group**	$P2_1$ (No. 4)	$P\bar{1}$ (No. 2)	$P\bar{1}$ (No. 2)
***a* [Å]**	9.8045(4)	9.8089(2)	10.9867(5)
***b* [Å]**	18.8907(8)	12.0987(3)	11.9720(4)
***c* [Å]**	13.4286(5)	20.7636(5)	19.1155(5)
α **[°]**	90	83.7617(19)	73.886(3)
β **[°]**	109.535(5)	86.3852(19)	80.840(3)
γ **[°]**	90	85.1419(19)	71.036(4)
V **[Å^3]**	2343.99(17)	2437.14(10)	2277.82(15)
Z	2	1	2
$D_{calc.}$ **[mg m^{-3}]**	1.179	1.152	1.181
**Absorption coefficient [mm^{-1}]**	1.20	1.15	1.20
θ **range for data collection [°]**	4.204 to 73.570	3.687 to 73.506	4.024 to 73.517
**Reflections collected**	14919	30261	27455
**Independent reflections**	7520 [R_{int} 0.059]	9748 [R_{int} 0.025]	9098 [R_{int} 0.036]
**Reflections with $I > 2\sigma(I)$**	6230	8675	7721
**Data/restraints/parameters**	7520 / 1 / 536	9748 / 0 / 568	9098 / 0 / 528
**Final *R* indices [$I > 2\sigma(I)$]**	R_1 = 0.051, wR_2 = 0.115	R_1 = 0.048, wR_2 = 0.128	R_1 = 0.040, wR_2 = 0.099
**Final *R* indices (all data)**	R_1 = 0.066, wR_2 = 0.124	R_1 = 0.055, wR_2 = 0.133	R_1 = 0.049, wR_2 = 0.105
**GOOF on F^2**	0.98	1.054	1.032
**Largest diff. peak/hole [e.Å^{-3}]**	0.46 and –0.23	0.90 and –0.39	0.39 and –0.37

Table S3 Crystal structure and refinement data for $[K_2(THF)_3][(fluN)_2]$ (**3**) and Al(NON){*i*PrNC(N=CMe$_2$)NC(H)flu)} (**4**).

	**3**	**4**
**Empirical formula**	$C_{38}H_{40}K_2N_2O_3$	$C_{48}H_{68}AlN_5OSi_2$
**CCDC Number**	2292510	2292511
$\boldsymbol{M_r}$	650.92	814.23
***T* [K]**	150.0(1)	120.0(1)
**Crystal size [mm]**	0.39 × 0.07 × 0.04	0.21 × 0.15 × 0.05
**Crystal system**	Triclinic	Orthorhombic
**Space group**	$P\overline{1}$ (No. 2)	$Pca2_1$ (No. 29)
***a* [Å]**	9.8256(9)	21.3210(3)
***b* [Å]**	13.2974(12)	12.0868(2)
***c* [Å]**	13.7313(11)	36.4298(7)
**α [°]**	84.960(7)	90
**β [°]**	73.086(7)	90
**γ [°]**	76.592(8)	90
**V [Å^3]**	1669.3(2)	9388.0(3)
Z	2	8
**$D_{calc.}$ [mg m^{-3}]**	1.295	1.152
**Absorption coefficient [mm^{-1}]**	2.82	1.17
**θ range for data collection [°]**	4.746 to 73.709	3.640 to 73.503
**Reflections collected**	18813	40276
**Independent reflections**	6657 [R_{int} 0.034]	17197 [R_{int} 0.049]
**Reflections with $I > 2\sigma(I)$**	4554	15466
**Data/restraints/parameters**	6657 / 49 / 488	17197 / 1 / 1059
**Final *R* indices [$I > 2\sigma(I)$]**	R_1 = 0.078, wR_2 = 0.211	R_1 = 0.052, wR_2 = 0.116
**Final *R* indices (all data)**	R_1 = 0.108, wR_2 = 0.238	R_1 = 0.060, wR_2 = 0.120
**GOOF on F^2**	0.997	1.079
**Largest diff. peak/hole [e.Å^{-3}]**	0.76 and –0.49	1.22 and –0.54

Computational Details

DFT calculations were run with Gaussian 16 (C.01).[44] The Al, Si and K centres were described with the Stuttgart RECPs and associated basis sets,[45] and the 6-31G** basis set was used for all other atoms (BS1).[46] A polarization function was also added to Al (ζ_d = 0.190), Si (ζ_d = 0.284) and K (ζ_d = 1.000).[47] Initial BP86 optimizations were performed using the 'grid = ultrafine' option,[48] with all stationary points being fully characterized via analytical frequency calculations as minima (all positive eigenvalues) or transition states (one imaginary eigenvalue). All energies were recomputed with a larger basis set featuring 6-311++G** basis sets on all atoms (BS2). Corrections for the effect of THF (ε = 7.4257) solvent were run using the polarizable continuum model and BS1[49] using the keyword "scrf=TetraHydroFuran" within Gaussian. Single-point dispersion corrections to the BP86 results employed Grimme's D3 parameter set with Becke-Johnson damping as implemented in Gaussian.[50] Natural Bonding Orbital (NBO7)[51] analyses were performed on the BP86-optmised geometries at the BP86/6-311++G** level (*i.e.* BS2), within Gaussian 16 (C.01), this approach is referred to as BP86/BS2//BP86/BS1.

Breakdown of Energy Contributions

The following tables detail the evolution of the relative energies as the successive corrections to the initial SCF energy are included. Terms used are:

ΔE_{BSI}	SCF energy computed with the BP86 functional with BS1
ΔH_{BSI}	Enthalpy at 0 K with BS1
ΔG_{BSI}	Free energy at 298.15 K and 1 atm with BS1
$\Delta G_{BSI/THF}$	Free energy corrected for THF solvent with BS1
$\Delta G_{BSI/THF+D3BJ}$	Free energy corrected for THF and dispersion effects with BS1
ΔE_{BS2}	SCF energy computed with the BP86 functional with BS2
ΔG_{THF}	Free energy corrected for basis set (BS2), dispersion effects and THF solvent

In each case the final data used in the main article are highlighted in bold. Free energies are quoted in kcal mol^{-1}, and include all three single point corrections (for BS2, solvation and dispersion).

Table S4 Relative energies for computed structures (in kcal mol^{-1}). Data in bold is at the same level as free energies used in the text.

	ΔE_{BSI}	ΔH_{BSI}	ΔG_{BSI}	$\Delta G_{BSI/THF}$	$\Delta G_{BSI/THF+D3BJ}$	ΔE_{BS2}	ΔG_{THF}
**1$_{DFT}$**	0.0	0.0	0.0	0.0	0.0	0.0	**0.0**
**1'$_{DFT}$**	96.9	95.7	87.6	12.6	21.2	95.3	**19.6**
**2·THF$_{DFT}$**	0.0	0.0	0.0	0.0	0.0	0.0	**0.0**
**2$_{DFT}$**	9.3	7.0	-10.5	-10.6	10.6	5.7	**7.0**
**A**	5.3	5.2	7.4	8.3	-2.4	5.4	**-2.3**
**TS(A-B)**	13.1	12.69	17.5	19.4	7.2	14.8	**8.9**
**B**	2.7	3.9	9.1	10.4	-0.6	7.1	**3.8**
**TS(B-C)**	9.0	7.2	12.6	14.5	1.6	12.0	**4.6**
**C**	-10.2	-9.3	-5.1	-4.3	-17.0	-7.8	**-14.6**
**4$_{DFT}$**	-15.9	-14.9	-10.7	-9.2	-20.8	-13.7	**-18.7**

Figure S25 Wiberg Bond Indices (WBIs, blue) and Natural Charges (red) from NBO7 calculation (BP86/BS2//BP86/BS1) for **1**$_{\mathbf{DFT}}$ and **1'**$_{\mathbf{DFT}}$

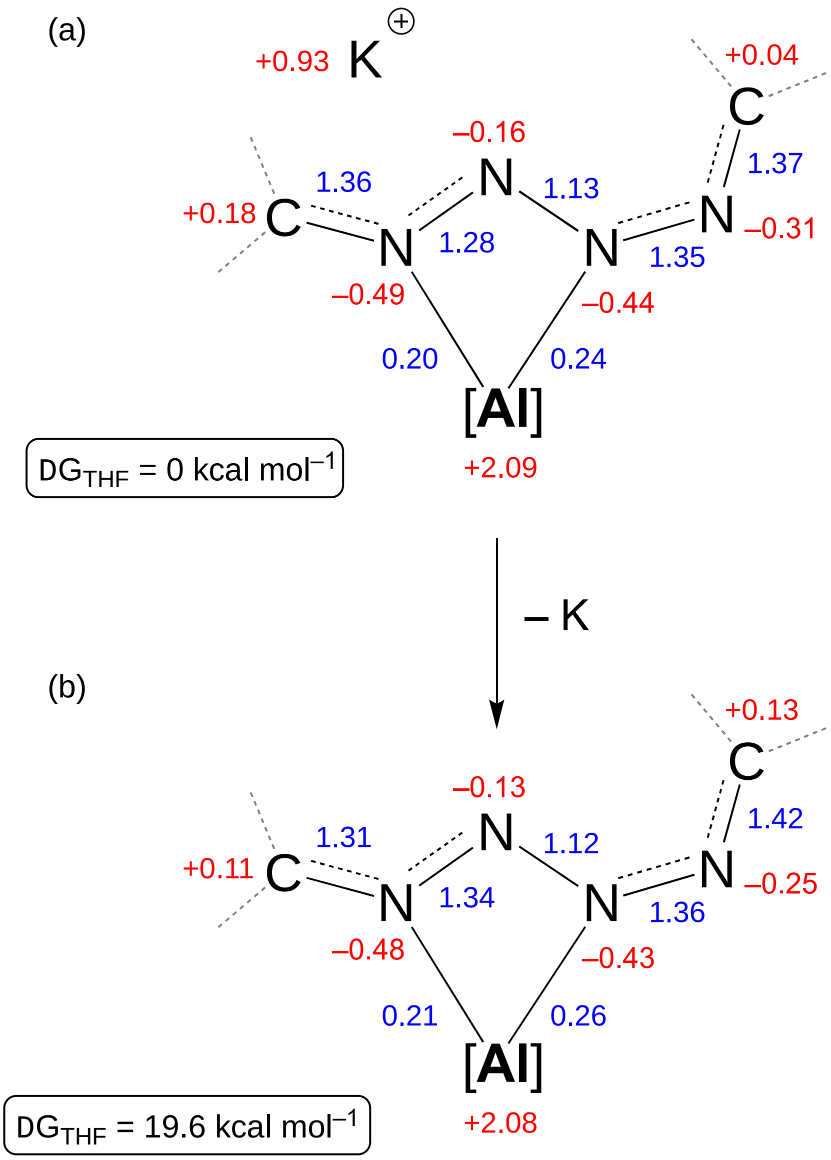

Table S5 Selected orbital contribution data for the [(fluN2)2] component from NBO7 calculation (BP86/BS2//BP86/BS1) for **1'DFT**

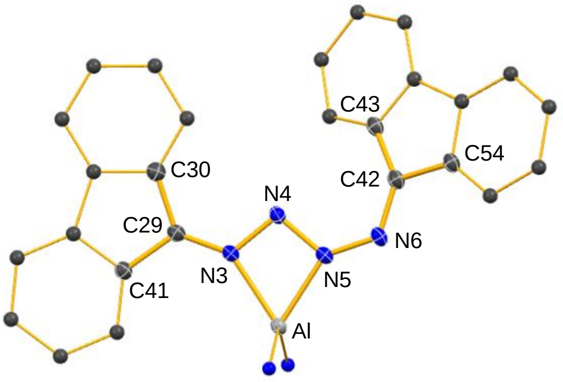

**Bond Atom1-Atom2**	**Bond Number**	**Atom1 AO Contributions**	**Atom2 AO Contributions**
N3-N4	BD(1)	52.7% **N3**<br>*26.26% s*<br>*73.63% p*	47.25% **N4**<br>*25.1% s*<br>*74.7% p*
	BD(2)	53.33% **N3**<br>*99.89% p*	46.67% **N4**<br>*99.78% p*
N3-C29	BD(1)	61.06% **N3**<br>*43.86% s*<br>*56.07% p*	38.94% **C29**<br>*31.34% s*<br>*68.58% p*
N4-N5	BD(1)	47.72% **N4**<br>*20.80% s*<br>*79.02% p*	52.28% **N5**<br>*23.86% s*<br>*76.05% p*
N5-N6	BD(1)	51.88% **N5**<br>*36.16% s*<br>*63.75% p*	48.12% **N6**<br>*28.94% s*<br>*70.91% p*
	BD(2)	62.15% **N5**<br>*99.90% p*	37.85% **N6**<br>*99.81% p*
N6-C42	BD(1)	59.70% **N6**<br>*40.74% s*<br>*67.12% p*	40.30% **C42**<br>*32.82% s*<br>*67.12% p*

Figure S26 Wiberg Bond Indices (WBIs, blue) and Natural Charges (red) from NBO7 calculation (BP86/BS2//BP86/BS1) for **2·THF$_{DFT}$** and **2$_{DFT}$**.

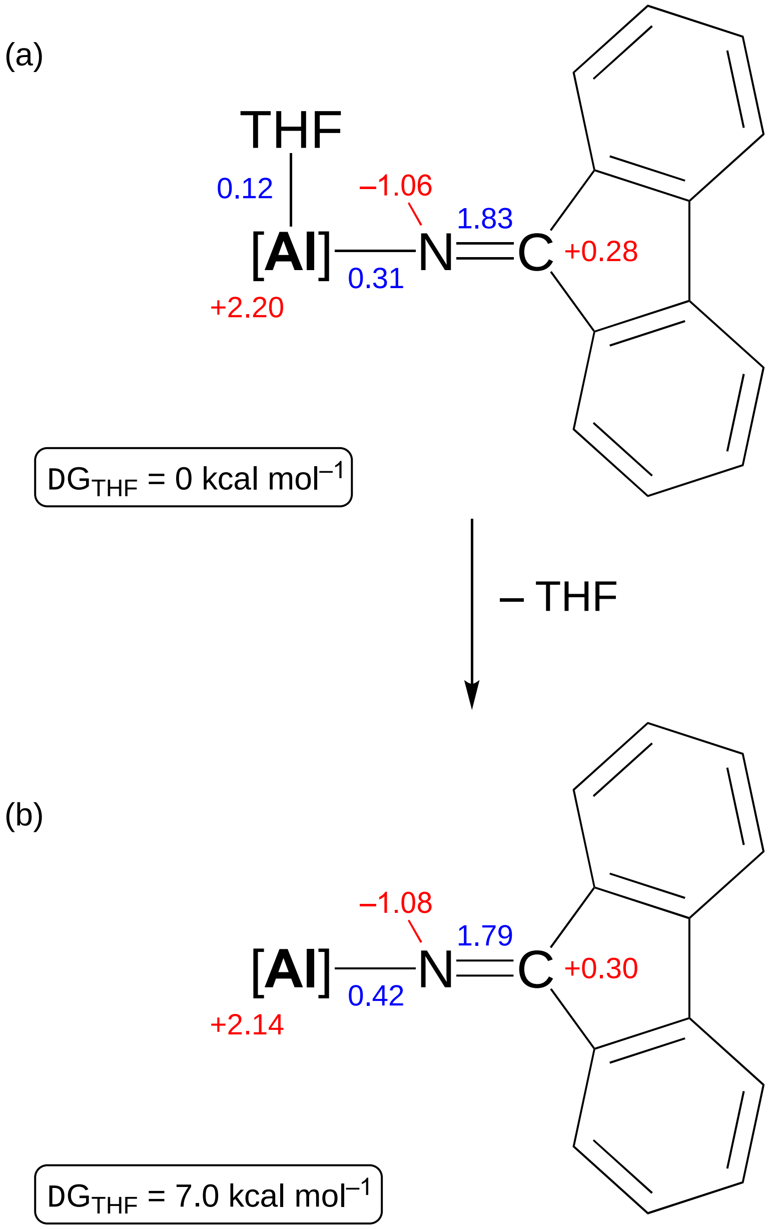

Table S6 Selected orbital contribution data for the [(fluN$_2$)$_2$] component from NBO7 calculation (BP86/BS2//BP86/BS1) for **2$_{DFT}$**

**Bond**<br>**Atom1-Atom2**	**Bond Number**	**Atom1**<br>**AO Contributions**	**Atom2**<br>**AO Contributions**
N3-C29	BD(1)	58.3% **N3**<br>*54.1% s*<br>*45.8% p*	41.7% **C29**<br>*35.8% s*<br>*64.1% p*
	BD(2)	62.0% **N3**<br>*99.97% p*	38.0% **C29**<br>*99.91% p*

Cartesian Coordinates and Computed Energies [in Hartrees] for Calculated Structures

K+
SCF (BP86) Energy = -28.1583832893

Enthalpy 0K = -28.158383
Enthalpy 298K = -28.156023
Free Energy 298K = -28.173559
SCF (BP86-D3BJ) Energy = -28.1583832894
SCF (THF) Energy = -28.2678486395
SCF (BS2) Energy = -599.799233449

K 0.0 0.0 0.0

THF
SCF (BP86) Energy = -232.447574761
Enthalpy 0K = -232.333967
Enthalpy 298K = -232.327978
Free Energy 298K = -232.363078
Lowest Frequency = 23.1459 cm^{-1}
Second Frequency = 275.4150 cm^{-1}
SCF (BP86-D3BJ) Energy = -232.461052284
SCF (THF) Energy = -232.449966822
SCF (BS2) Energy = -232.511411715

```
C   1.11953  0.49016  0.18507
O  -0.01624  1.21238 -0.30202
C  -1.14796  0.45256  0.13889
C  -0.75507 -1.04216 -0.02233
C   0.80213 -0.99935 -0.08328
H   2.01008  0.86631 -0.34439
H   1.25822  0.66776  1.27604
H  -1.37417  0.67882  1.20507
H  -2.01389  0.75889 -0.47051
H  -1.12708 -1.64979  0.81904
H  -1.17361 -1.46775 -0.94858
H   1.27911 -1.66696  0.65304
H   1.15943 -1.29360 -1.08365
```

iPrNCNiPr
SCF (BP86) Energy = -384.663514546
Enthalpy 0K = -384.465592
Enthalpy 298K = -384.452922
Free Energy 298K = -384.504951
Lowest Frequency = 15.3737 cm^{-1}
Second Frequency = 23.0662 cm^{-1}
SCF (BP86-D3BJ) Energy = -384.692421409
SCF (THF) Energy = -384.666823039
SCF (BS2) Energy = -384.762347875

```
N   1.03069  0.34424  0.49067
N  -1.22367  0.87240 -0.36198
C  -0.10044  0.51585  0.01601
C   3.27561  1.05762 -0.17613
H   2.88622  1.98340 -0.62989
H   4.19889  0.76982 -0.70771
H   3.52907  1.27122  0.87606
C   2.76824 -1.37933  0.35674
H   2.01942 -2.18478  0.28203
H   3.00756 -1.23187  1.42352
H   3.68371 -1.70550 -0.16588
C  -2.45289  0.06336 -0.43646
H  -2.88279  0.26533 -1.43693
C  -3.44745  0.58341  0.61743
H  -4.42425  0.08247  0.50585
H  -3.59514  1.66975  0.51005
H  -3.06912  0.38651  1.63525
C  -2.20540 -1.44702 -0.30239
H  -1.50313 -1.80398 -1.07451
H  -3.14982 -2.00667 -0.41291
H  -1.78029 -1.68509  0.68844
C   2.23660 -0.07301 -0.25433
H   1.98498 -0.25221 -1.31941
```

1_{DFT}
SCF (BP86) Energy = -2537.31717402
Enthalpy 0K = -2536.305376
Enthalpy 298K = -2536.236589
Free Energy 298K = -2536.407401
Lowest Frequency = 14.9142 cm^{-1}
Second Frequency = 18.6346 cm^{-1}
SCF (BP86-D3BJ) Energy = -2537.69244444
SCF (THF) Energy = -2537.33929884
SCF (BS2) Energy = -3921.25897500

```
K  -2.97541 -0.39339 -2.20503
Si  2.98044 -2.42393  1.35383
Si  2.68663 -2.27909 -1.77802
Al  0.80026 -0.70127  0.10367
O   3.39963 -2.03084 -0.25975
N   1.57080 -1.38475  1.64376
N   1.05125 -1.59767 -1.53376
N   0.72826  1.32127 -0.10056
N  -0.60452  1.41991  0.11611
N  -0.98125  0.08392  0.28683
N  -2.23089 -0.29162  0.46478
C   1.16447 -1.09190  3.00812
C   1.67633  0.05712  3.69591
C   1.31638  0.27190  5.04138
H   1.71512  1.15197  5.55842
C   0.46494 -0.59751  5.72696
H   0.20269 -0.41172  6.77400
C  -0.06002 -1.69839  5.04663
H  -0.74837 -2.37356  5.56755
C   0.26510 -1.96464  3.70156
C   2.56388  1.11429  3.03719
H   2.78951  0.77652  2.01168
C   1.81941  2.46739  2.94443
H   0.85172  2.36378  2.42847
H   2.42527  3.20738  2.39417
H   1.61959  2.87471  3.95166
C   3.90575  1.32027  3.77757
H   3.74736  1.74846  4.78337
H   4.54401  2.02480  3.21641
H   4.46156  0.37770  3.90255
C  -0.41101 -3.17075  3.04732
H  -0.00374 -3.26573  2.02568
C  -1.93802 -2.94630  2.94036
H  -2.38402 -2.79979  3.93983
H  -2.43083 -3.82344  2.48397
H  -2.17536 -2.05943  2.33030
C  -0.12723 -4.48672  3.80799
H   0.95154 -4.65247  3.96355
H  -0.53158 -5.35208  3.25322
H  -0.60520 -4.48440  4.80350
C   2.65921 -4.29106  1.45380
H   1.75458 -4.59820  0.90452
H   2.53916 -4.61072  2.50192
H   3.52335 -4.83600  1.03568
C   4.47819 -2.02278  2.42667
H   5.26576 -2.77842  2.26481
H   4.21188 -2.02941  3.49654
```

H	4.88969	-1.03292	2.17646
C	2.69736	-4.10664	-2.27788
H	3.74325	-4.42387	-2.43213
H	2.16409	-4.25067	-3.23326
H	2.24630	-4.76909	-1.52426
C	3.77811	-1.37016	-3.02377
H	3.83385	-0.28825	-2.82841
H	3.42596	-1.52917	-4.05645
H	4.80031	-1.78226	-2.95639
C	0.06353	-1.73733	-2.56200
C	-0.08436	-0.75317	-3.60155
C	-1.04371	-0.95417	-4.62075
H	-1.12500	-0.20937	-5.42121
C	-1.87154	-2.08523	-4.64773
H	-2.58904	-2.23377	-5.46266
C	-1.74540	-3.03662	-3.62433
H	-2.38275	-3.92810	-3.63835
C	-0.79538	-2.89383	-2.59030
C	0.73918	0.53460	-3.65217
H	1.47045	0.49220	-2.82846
C	-0.15652	1.77578	-3.42634
H	-0.92335	1.86239	-4.21994
H	0.44541	2.69883	-3.44872
H	-0.65719	1.75205	-2.44217
C	1.50882	0.68653	-4.98431
H	2.12386	-0.19900	-5.20929
H	2.17647	1.56326	-4.93655
H	0.82140	0.84184	-5.83524
C	-0.74005	-3.97552	-1.51114
H	0.16277	-3.77815	-0.90958
C	-0.64282	-5.40346	-2.09215
H	-1.56917	-5.69505	-2.61847
H	-0.49310	-6.13280	-1.27733
H	0.19387	-5.50692	-2.80094
C	-1.95999	-3.87092	-0.56868
H	-2.02717	-2.87862	-0.08950
H	-1.89723	-4.62698	0.23243
H	-2.89920	-4.05766	-1.12356
C	1.45244	2.40447	-0.42122
C	0.98544	3.77598	-0.62585
C	-0.29744	4.35724	-0.56594
H	-1.16826	3.75426	-0.30217
C	-0.43108	5.73087	-0.83505
H	-1.42344	6.19112	-0.78143
C	0.68781	6.52101	-1.16360
H	0.55829	7.58928	-1.36777
C	1.97171	5.94983	-1.23036
H	2.83898	6.56831	-1.48748
C	2.12431	4.58320	-0.96346
C	3.31358	3.72720	-0.96090
C	4.66317	4.01618	-1.20737
H	4.97697	5.03554	-1.45867
C	5.61233	2.98293	-1.12236
H	6.66953	3.19767	-1.31026
C	5.21211	1.67275	-0.79042
H	5.96066	0.87657	-0.72135
C	3.86232	1.36804	-0.54104
H	3.58525	0.34140	-0.27929
C	2.90279	2.39357	-0.62972
C	-3.29147	0.52604	0.66375
C	-3.51614	1.91698	1.10169
C	-2.67424	2.89840	1.66881
H	-1.60825	2.70214	1.79179
C	-3.21629	4.12969	2.06786
H	-2.55360	4.88683	2.50029
C	-4.59288	4.40405	1.92977
H	-4.99230	5.37201	2.25036
C	-5.45507	3.42655	1.41181
H	-6.53170	3.61923	1.34008
C	-4.92904	2.18668	1.01517
C	-5.61094	0.98362	0.54547
C	-6.96040	0.71747	0.26050
H	-7.71647	1.50362	0.36762
C	-7.33646	-0.56958	-0.16054
H	-8.38725	-0.78590	-0.37967
C	-6.37044	-1.59241	-0.28253
H	-6.68406	-2.60111	-0.57430
C	-5.01487	-1.33916	-0.00859
H	-4.27793	-2.14994	-0.05582
C	-4.62363	-0.04515	0.40208

1′$_{DFT}$

SCF (BP86) Energy = -2509.00432073
Enthalpy 0K = -2507.994469
Enthalpy 298K = -2507.927625
Free Energy 298K = -2508.094208
Lowest Frequency = 12.3922 cm^{-1}
Second Frequency = 16.6889 cm^{-1}
SCF (BP86-D3BJ) Energy = -2509.36576877
SCF (THF) Energy = -2509.03661083
SCF (BS2) Energy = -3321.30786371

Si	2.40029	-2.52536	1.76551
Si	2.80214	-2.40981	-1.32730
Al	0.59115	-0.70523	0.06285
O	3.23543	-2.22659	0.30953
N	1.03740	-1.39071	1.74465
N	1.18823	-1.67989	-1.42031
N	0.69523	1.31963	-0.15215
N	-0.63319	1.49527	-0.18805
N	-1.12651	0.18673	-0.06921
N	-2.39758	-0.12989	-0.11301
C	0.40153	-1.05144	3.00235
C	0.83332	0.08512	3.76415
C	0.24959	0.33974	5.02113
H	0.59190	1.21092	5.59187
C	-0.75456	-0.47532	5.54900
H	-1.19333	-0.25834	6.52947
C	-1.20550	-1.55805	4.79063
H	-2.01566	-2.18643	5.17873
C	-0.65643	-1.86302	3.52902
C	1.86855	1.09161	3.26164
H	2.25430	0.72517	2.29632
C	1.21160	2.47151	3.02212
H	0.34587	2.39477	2.34605
H	1.93571	3.17243	2.57319
H	0.85731	2.90782	3.97401
C	3.06919	1.25569	4.22175
H	2.75885	1.71151	5.17978
H	3.82598	1.91953	3.76851
H	3.55138	0.29242	4.45282
C	-1.27364	-3.03677	2.76661
H	-0.67879	-3.18693	1.84953
C	-2.72681	-2.70946	2.34989
H	-3.34953	-2.49490	3.23695
H	-3.18253	-3.56552	1.82106
H	-2.77004	-1.83453	1.68120
C	-1.24663	-4.34943	3.58365
H	-0.23860	-4.58247	3.96608
H	-1.58368	-5.19851	2.96244
H	-1.92372	-4.29340	4.45510
C	1.93623	-4.36863	1.81114
H	1.17129	-4.62923	1.06179
H	1.54790	-4.65467	2.80243
H	2.83706	-4.97600	1.61393
C	3.62980	-2.21143	3.16848

H	4.35294	-3.04319	3.22650
H	3.11511	-2.13213	4.14024
H	4.18792	-1.27866	2.99276
C	2.85025	-4.23730	-1.84270
H	3.89721	-4.58754	-1.84086
H	2.45512	-4.35577	-2.86600
H	2.26736	-4.88599	-1.17152
C	4.17178	-1.54837	-2.30944
H	4.22702	-0.47036	-2.09327
H	4.02427	-1.68504	-3.39358
H	5.14205	-2.00369	-2.04262
C	0.44539	-1.85217	-2.65135
C	0.55136	-0.91036	-3.72390
C	-0.15131	-1.14967	-4.92270
H	-0.06801	-0.41931	-5.73599
C	-0.95481	-2.27929	-5.09500
H	-1.49225	-2.44291	-6.03591
C	-1.07756	-3.18805	-4.03912
H	-1.72645	-4.06390	-4.15507
C	-0.39761	-2.99938	-2.82121
C	1.35447	0.38666	-3.61971
H	1.89244	0.37204	-2.65707
C	0.41306	1.61380	-3.61978
H	-0.14322	1.68166	-4.57267
H	0.99006	2.54566	-3.49385
H	-0.32391	1.56140	-2.80283
C	2.39749	0.53161	-4.75137
H	3.07117	-0.33920	-4.80961
H	3.01546	1.43156	-4.58661
H	1.91000	0.64224	-5.73708
C	-0.65072	-3.99675	-1.69082
H	0.10823	-3.80319	-0.91338
C	-0.53007	-5.47233	-2.12974
H	-1.32793	-5.75225	-2.84094
H	-0.63063	-6.13983	-1.25486
H	0.43833	-5.68370	-2.61209
C	-2.03936	-3.74278	-1.06264
H	-2.14020	-2.70403	-0.70420
H	-2.21605	-4.42544	-0.21201
H	-2.83682	-3.91741	-1.80810
C	1.54010	2.35824	-0.34150
C	1.21257	3.74004	-0.67298
C	-0.01321	4.38725	-0.94297
H	-0.95139	3.83051	-0.90665
C	-0.00343	5.75468	-1.26180
H	-0.95340	6.25990	-1.46910
C	1.20258	6.48541	-1.31791
H	1.18227	7.55271	-1.56645
C	2.43007	5.85098	-1.06211
H	3.36767	6.41756	-1.11501
C	2.44288	4.48500	-0.74342
C	3.54560	3.57337	-0.45164
C	4.93106	3.78437	-0.37746
H	5.34917	4.78176	-0.55971
C	5.77733	2.70798	-0.06399
H	6.86035	2.86294	-0.00293
C	5.23582	1.42625	0.17565
H	5.90178	0.59133	0.42017
C	3.85190	1.19686	0.10609
H	3.46701	0.18994	0.29930
C	2.99034	2.26723	-0.21244
C	-3.45553	0.69445	-0.08202
C	-3.72005	2.10307	0.28539
C	-2.92217	3.13339	0.82725
H	-1.85349	2.97222	0.97433
C	-3.50210	4.36832	1.15833
H	-2.86516	5.16139	1.56604
C	-4.88040	4.60158	0.97513
H	-5.31273	5.57376	1.23871
C	-5.70121	3.57960	0.47405
H	-6.77957	3.74187	0.35610
C	-5.13532	2.33848	0.14269
C	-5.77660	1.10705	-0.31047
C	-7.11585	0.80271	-0.60740
H	-7.89311	1.57286	-0.53117
C	-7.44817	-0.50270	-1.00359
H	-8.48908	-0.75386	-1.23825
C	-6.44635	-1.49371	-1.10681
H	-6.72089	-2.50814	-1.41942
C	-5.10478	-1.19698	-0.82276
H	-4.32662	-1.95950	-0.91762
C	-4.76382	0.10524	-0.41381

2·THF$_{DFT}$

SCF (BP86) Energy = -2076.94907687
Enthalpy 0K = -2075.998984
Enthalpy 298K = -2075.938083
Free Energy 298K = -2076.092590
Lowest Frequency = 16.2225 cm^{-1}
Second Frequency = 18.4170 cm^{-1}
SCF (BP86-D3BJ) Energy = -2077.27127671
SCF (THF) Energy = -2076.95852704
SCF (BS2) Energy = -2889.14310458

Si	1.71265	-3.21527	0.18383
Si	-1.25268	-3.23064	-0.69221
Al	0.00105	-0.55656	0.25438
O	0.07203	-3.65297	0.29954
O	-0.29309	-0.44735	2.25577
N	1.65672	-1.44857	0.04792
N	-1.50815	-1.50359	-0.36145
N	0.16344	1.23075	0.00586
C	2.89505	-0.78240	-0.29869
C	3.25906	-0.58646	-1.67244
C	4.50268	-0.00607	-1.99005
H	4.76521	0.13784	-3.04420
C	5.40574	0.39177	-0.99934
H	6.37324	0.82916	-1.26833
C	5.04264	0.23311	0.34042
H	5.73164	0.56386	1.12652
C	3.80686	-0.33465	0.71173
C	2.31428	-0.91318	-2.82810
H	1.48363	-1.50774	-2.40979
C	2.97776	-1.73134	-3.95797
H	3.74768	-1.14293	-4.48790
H	2.22355	-2.02402	-4.70994
H	3.45872	-2.64786	-3.58068
C	1.72051	0.39230	-3.40435
H	1.19643	0.97079	-2.62629
H	1.00773	0.17106	-4.21846
H	2.51864	1.03298	-3.82038
C	3.47509	-0.37578	2.20310
H	2.49057	-0.86616	2.29639
C	4.50452	-1.17756	3.03030
H	4.61701	-2.20843	2.66045
H	4.20113	-1.22686	4.09216
H	5.50028	-0.70112	2.99869
C	3.36512	1.06231	2.76440
H	4.34806	1.56565	2.75320
H	3.01405	1.05730	3.81317
H	2.68049	1.68012	2.15933
C	2.52110	-3.92929	1.75099
H	2.25944	-3.40538	2.68381
H	3.61979	-3.91512	1.65477
H	2.21140	-4.98413	1.85329
C	2.57739	-4.09274	-1.25592

```
H   2.63304 -5.17390 -1.04035
H   3.60865 -3.71783 -1.37013
H   2.05391 -3.95907 -2.21398
C  -2.67987 -4.36333 -0.18558
H  -2.47795 -5.37967 -0.56554
H  -3.63601 -4.02471 -0.61761
H  -2.78876 -4.42397  0.90795
C  -0.85339 -3.64573 -2.50218
H  -0.10085 -2.97417 -2.94490
H  -1.76701 -3.57786 -3.11667
H  -0.48243 -4.68286 -2.57435
C  -2.79614 -0.90707 -0.64054
C  -3.06028 -0.26517 -1.89613
C  -4.33265  0.28791 -2.14493
H  -4.51600  0.77329 -3.11024
C  -5.36030  0.23769 -1.20074
H  -6.34183  0.67228 -1.41826
C  -5.10843 -0.37837  0.02783
H  -5.90453 -0.42110  0.78028
C  -3.85705 -0.95272  0.32845
C  -2.01818 -0.12646 -3.00650
H  -1.08578 -0.59619 -2.64791
C  -1.72828  1.36028 -3.31489
H  -2.63687  1.87664 -3.67171
H  -0.96256  1.45388 -4.10357
H  -1.36948  1.89283 -2.42089
C  -2.45801 -0.85130 -4.30000
H  -2.70902 -1.90854 -4.11656
H  -1.65475 -0.81596 -5.05717
H  -3.34965 -0.37181 -4.74192
C  -3.69442 -1.56981  1.71858
H  -2.71053 -2.06877  1.73187
C  -3.70363 -0.47092  2.80631
H  -2.93720  0.29552  2.61164
H  -3.52890 -0.90199  3.80973
H  -4.68138  0.04159  2.83571
C  -4.78169 -2.61736  2.05184
H  -5.77735 -2.14887  2.14478
H  -4.56239 -3.10570  3.01850
H  -4.85284 -3.40072  1.28223
C  -0.01377  2.49108 -0.02098
C  -1.27881  3.28455  0.20379
C  -2.58255  2.83470  0.42878
H  -2.80695  1.76171  0.42744
C  -3.60054  3.78961  0.62239
H  -4.62992  3.45762  0.79118
C  -3.30481  5.16437  0.58529
H  -4.10780  5.89414  0.73613
C  -1.99200  5.61942  0.34651
H  -1.77885  6.69351  0.31094
C  -0.97816  4.67302  0.15138
C   0.46743  4.83130 -0.13222
C   1.26288  5.97354 -0.28923
H   0.83649  6.97903 -0.20201
C   2.63484  5.80605 -0.56631
H   3.26932  6.68999 -0.69350
C   3.20113  4.52290 -0.68299
H   4.26908  4.41876 -0.90109
C   2.40466  3.37201 -0.52277
H   2.82749  2.36514 -0.61183
C   1.04464  3.53952 -0.24977
C  -0.26258 -1.60157  3.16924
H   0.36778 -2.37066  2.70317
H  -1.29279 -1.98397  3.26486
C   0.26258 -1.03093  4.48466
H   1.36463 -0.97516  4.47010
H  -0.04258 -1.64125  5.34970
C  -0.35597  0.38236  4.49357
H  -1.41160  0.33680  4.81087
H   0.17376  1.08096  5.16052
C  -0.25127  0.81629  3.02832
H  -1.08039  1.44905  2.67869
H   0.70422  1.30824  2.79823
```

2_{DFT}

SCF (BP86) Energy = -1844.48668924
Enthalpy 0K = -1843.653853
Enthalpy 298K = -1843.597932
Free Energy 298K = -1843.746286
Lowest Frequency = 7.9414 cm^{-1}
Second Frequency = 13.8927 cm^{-1}
SCF (BP86-D3BJ) Energy = -1844.76155834
SCF (THF) Energy = -1844.49387248
SCF (BS2) Energy = -2656.62266236

```
Si  3.05251 -1.81390  0.74880
Si  0.83847 -3.40467 -0.84939
Al  0.34215 -0.42445 -0.01841
O   2.30297 -3.11366 -0.04422
N   2.13523 -0.33413  0.33208
N  -0.29534 -2.10083 -0.38052
N  -0.64483  1.04527 -0.06185
C   2.82381  0.92897  0.18059
C   3.30959  1.32051 -1.10690
C   3.95592  2.56381 -1.24507
H   4.32531  2.86720 -2.23088
C   4.14038  3.41717 -0.14987
H   4.64932  4.37868 -0.27700
C   3.66701  3.02760  1.10679
H   3.80271  3.69632  1.96462
C   3.00921  1.79590  1.29814
C   3.09669  0.44367 -2.34232
H   2.87451 -0.57520 -1.97956
C   4.33761  0.35345 -3.25478
H   4.56582  1.31869 -3.74033
H   4.16578 -0.38101 -4.06087
H   5.23374  0.04225 -2.69202
C   1.86732  0.92634 -3.14855
H   0.95813  0.97401 -2.52121
H   1.66368  0.25706 -4.00360
H   2.03655  1.94280 -3.54672
C   2.48478  1.45155  2.69213
H   2.06856  0.43136  2.63954
C   3.60737  1.45189  3.75344
H   4.43219  0.77370  3.47482
H   3.21440  1.13070  4.73421
H   4.04031  2.45927  3.88598
C   1.33870  2.40196  3.10852
H   1.69143  3.44623  3.17892
H   0.93423  2.11693  4.09602
H   0.51523  2.37695  2.37627
C   3.00614 -2.22479  2.59811
H   1.97059 -2.25636  2.97637
H   3.56496 -1.48432  3.19415
H   3.46062 -3.21557  2.77055
C   4.84441 -1.68599  0.16593
H   5.39989 -2.58410  0.48505
H   5.34191 -0.80224  0.59830
H   4.90884 -1.61503 -0.93126
C   0.17306 -5.09426 -0.33049
H   0.87234 -5.88183 -0.65862
H  -0.80872 -5.29254 -0.79135
H   0.06093 -5.16873  0.76262
C   1.23390 -3.40601 -2.70252
H   1.57276 -2.41246 -3.04116
H   0.35529 -3.69051 -3.30515
```

```
H   2.04098 -4.12886 -2.91226
C  -1.69679 -2.38754 -0.17608
C  -2.62510 -2.29545 -1.25531
C  -3.98736 -2.56075 -1.00969
H  -4.70145 -2.48279 -1.83762
C  -4.44659 -2.91373  0.26308
H  -5.50933 -3.11809  0.43179
C  -3.53195 -3.00625  1.32020
H  -3.88974 -3.28542  2.31717
C  -2.16001 -2.75404  1.12759
C  -2.19530 -1.89261 -2.66568
H  -1.09847 -1.77645 -2.64893
C  -2.80300 -0.53089 -3.07251
H  -3.90586 -0.57851 -3.10791
H  -2.44816 -0.22968 -4.07408
H  -2.52553  0.25877 -2.35550
C  -2.54139 -2.97966 -3.70778
H  -2.10368 -3.95636 -3.43860
H  -2.16204 -2.69604 -4.70536
H  -3.63301 -3.12164 -3.79775
C  -1.20242 -2.83228  2.31844
H  -0.18649 -2.96306  1.90653
C  -1.20857 -1.50565  3.11431
H  -0.96687 -0.63724  2.47222
H  -0.47621 -1.53261  3.94076
H  -2.20580 -1.31292  3.54818
C  -1.48060 -4.02351  3.25826
H  -2.43647 -3.91142  3.79981
H  -0.68542 -4.10098  4.01991
H  -1.51907 -4.97665  2.70441
C  -1.46786  2.01977 -0.02306
C  -2.87947  2.03072  0.49271
C  -3.64403  0.98315  1.01133
H  -3.22364 -0.02499  1.09207
C  -4.96771  1.25362  1.41008
H  -5.58636  0.44805  1.81822
C  -5.50261  2.54789  1.27883
H  -6.53433  2.74024  1.59256
C  -4.73554  3.60171  0.74053
H  -5.16981  4.60169  0.63404
C  -3.41970  3.33704  0.34403
C  -2.38435  4.20718 -0.26173
C  -2.39474  5.56544 -0.59958
H  -3.28190  6.18558 -0.43143
C  -1.23027  6.12907 -1.16075
H  -1.22352  7.19085 -1.42983
C  -0.07606  5.35299 -1.37295
H   0.81834  5.81724 -1.80087
C  -0.06141  3.98632 -1.03229
H   0.83387  3.37174 -1.17511
C  -1.22005  3.42605 -0.48955
```

A

SCF (BP86) Energy = -2229.15655076
Enthalpy 0K = -2228.122269
Enthalpy 298K = -2228.054718
Free Energy 298K = -2228.222605
Lowest Frequency = 17.5553 cm^{-1}
Second Frequency = 21.0864 cm^{-1}
SCF (BP86-D3BJ) Energy = -2229.51123006
SCF (THF) Energy = -2229.16552526
SCF (BS2) Energy = -3041.38550144

```
Al -0.63101  0.36117  0.24796
Si -2.21403  2.93595 -0.45126
Si -3.77967  0.40061  0.41244
O  -3.21384  1.99862  0.57110
N  -0.61812  2.16800 -0.31478
N  -2.30340 -0.52510  0.08164
N   0.77932 -0.67111 -0.24985
N   2.18207 -0.36635  2.79152
N  -0.16141  0.28315  2.32449
C   0.55456  2.93238 -0.69176
C   1.24429  3.75109  0.26883
C   2.35787  4.51812 -0.12839
H   2.86709  5.13484  0.62137
C   2.82201  4.51774 -1.44579
H   3.68249  5.12936 -1.73793
C   2.16464  3.71657 -2.38123
H   2.52442  3.69826 -3.41617
C   1.05128  2.92209 -2.03869
C   0.85333  3.82066  1.74442
H  -0.06384  3.22222  1.85816
C   0.56700  5.26203  2.22656
H   1.48428  5.87739  2.21526
H   0.19620  5.25323  3.26768
H  -0.18222  5.77107  1.60191
C   1.95613  3.20622  2.63772
H   2.23147  2.18808  2.32348
H   1.63311  3.16743  3.69466
H   2.87425  3.82000  2.60128
C   0.45604  2.06736 -3.15884
H  -0.39533  1.50702 -2.73548
C  -0.06117  2.93106 -4.33340
H  -0.76094  3.71303 -3.99811
H  -0.58057  2.30242 -5.07840
H   0.77273  3.43562 -4.85321
C   1.49655  1.05326 -3.68820
H   2.38077  1.57197 -4.09859
H   1.06878  0.43797 -4.49766
H   1.84595  0.38069 -2.89023
C  -2.97400  2.97389 -2.19216
H  -2.86719  2.02349 -2.73831
H  -2.50446  3.76600 -2.79872
H  -4.04899  3.21225 -2.11297
C  -2.30462  4.70674  0.20652
H  -3.29426  5.12976 -0.03793
H  -1.53738  5.34732 -0.25887
H  -2.17739  4.74421  1.29928
C  -5.11241  0.25603 -0.92717
H  -6.01452  0.80221 -0.60160
H  -5.39353 -0.79999 -1.07873
H  -4.79055  0.66890 -1.89431
C  -4.67627  0.02665  2.04569
H  -4.03961  0.13364  2.93770
H  -5.09275 -0.99447  2.04134
H  -5.52115  0.72957  2.15184
C  -2.45597 -1.88884 -0.38085
C  -2.51798 -2.98383  0.54252
C  -2.70331 -4.29855  0.07039
H  -2.74305 -5.12093  0.79408
C  -2.83645 -4.57627 -1.29265
H  -2.98826 -5.60298 -1.64279
C  -2.76811 -3.51547 -2.20017
H  -2.85715 -3.72164 -3.27279
C  -2.58148 -2.18361 -1.77926
C  -2.33366 -2.79345  2.04474
H  -2.35720 -1.70701  2.22327
C  -3.45219 -3.44534  2.88593
H  -4.45221 -3.10844  2.56682
H  -3.33122 -3.19290  3.95454
H  -3.43329 -4.54671  2.80786
C  -0.95071 -3.32370  2.48788
H  -0.87259 -4.41157  2.31335
H  -0.78858 -3.14841  3.56797
```

H	-0.14109	-2.83747	1.91840
C	-2.45891	-1.11825	-2.86698
H	-2.45109	-0.13663	-2.36205
C	-3.62704	-1.14053	-3.87885
H	-3.61235	-2.05840	-4.49300
H	-3.54551	-0.28654	-4.57467
H	-4.60935	-1.08662	-3.38373
C	-1.11916	-1.28114	-3.61806
H	-0.26172	-1.21113	-2.93018
H	-1.01113	-0.50317	-4.39392
H	-1.06916	-2.26443	-4.11897
C	1.86328	-1.24345	-0.58903
H	2.88935	-2.12265	1.92367
C	3.25583	-0.66111	-0.66908
C	3.68360	0.65046	-0.44521
H	2.96112	1.43102	-0.18276
C	5.05438	0.94456	-0.58934
H	5.40802	1.96789	-0.42813
C	5.96715	-0.06398	-0.95129
H	7.02894	0.18223	-1.06149
C	5.53512	-1.38539	-1.18415
H	6.25559	-2.15819	-1.47432
C	4.17246	-1.68050	-1.04496
C	3.42096	-2.94333	-1.23408
C	3.84360	-4.22702	-1.60367
H	4.89890	-4.44072	-1.80671
C	2.87827	-5.24791	-1.71565
H	3.19310	-6.25611	-2.00649
C	1.51765	-4.99105	-1.46345
H	0.78439	-5.79813	-1.56226
C	1.08922	-3.70254	-1.08902
H	0.03317	-3.48631	-0.89421
C	2.04685	-2.69101	-0.97711
C	1.01452	-0.13554	2.55933
C	3.05205	-1.55634	2.85907
C	4.51234	-1.09315	2.92562
H	4.76393	-0.46396	2.05822
H	5.18007	-1.97006	2.92614
H	4.69452	-0.51521	3.84761
C	2.65998	-2.43300	4.05981
H	1.62203	-2.79214	3.97293
H	2.76682	-1.87296	5.00403
H	3.32454	-3.31163	4.10395
C	-1.11890	0.44056	3.49426
H	-1.97695	-0.19804	3.22627
C	-1.60650	1.88876	3.59999
H	-2.38553	1.94611	4.37996
H	-2.05108	2.22946	2.65224
H	-0.78420	2.56383	3.88826
C	-0.52373	-0.05644	4.81639
H	-0.21168	-1.11233	4.76044
H	-1.28615	0.02808	5.60802
H	0.34525	0.55210	5.12074

TS(A-B)

SCF (BP86) Energy = -2229.14420155
Enthalpy 0K = -2228.110013
Enthalpy 298K = -2228.043781
Free Energy 298K = -2228.206620
Lowest Frequency = -211.9979 cm^{-1}
Second Frequency = 19.6483 cm^{-1}
SCF (BP86-D3BJ) Energy = -2229.50126265
SCF (THF) Energy = -2229.15147978
SCF (BS2) Energy = -3041.37051464

Al	0.56039	-0.61199	0.37136
Si	1.14871	-3.26061	-1.00475
Si	3.44067	-1.65890	0.37277
O	2.31464	-2.92623	0.20650
N	-0.09964	-2.04471	-0.68063
N	2.40773	-0.22935	0.17141
N	-0.72301	0.78778	0.42714
N	-2.02087	0.55321	2.93536
N	-0.05795	-0.71730	2.24345
C	-1.46295	-2.23324	-1.12504
C	-2.42674	-2.93283	-0.31816
C	-3.74853	-3.08598	-0.78429
H	-4.46849	-3.62285	-0.15552
C	-4.16148	-2.58736	-2.02201
H	-5.19285	-2.72285	-2.36517
C	-3.22574	-1.92293	-2.81711
H	-3.53234	-1.53645	-3.79584
C	-1.89233	-1.73677	-2.40145
C	-2.10811	-3.55514	1.04314
H	-1.03799	-3.37969	1.23934
C	-2.37511	-5.08032	1.06136
H	-3.45579	-5.29477	0.98153
H	-2.02519	-5.52138	2.01223
H	-1.87203	-5.60478	0.23541
C	-2.92700	-2.90324	2.18162
H	-2.76612	-1.81704	2.25386
H	-2.66149	-3.35083	3.15703
H	-4.00872	-3.07437	2.03375
C	-0.96045	-1.01733	-3.37741
H	0.02416	-0.92564	-2.88769
C	-0.78238	-1.82868	-4.68294
H	-0.46494	-2.86507	-4.48365
H	-0.02753	-1.35734	-5.33742
H	-1.72895	-1.87871	-5.25029
C	-1.47062	0.40023	-3.71979
H	-2.46606	0.36309	-4.19623
H	-0.78597	0.90273	-4.42424
H	-1.55810	1.03106	-2.82120
C	1.97484	-3.18892	-2.71480
H	2.24397	-2.16914	-3.03089
H	1.30137	-3.61016	-3.47995
H	2.89034	-3.80588	-2.70604
C	0.62527	-5.05923	-0.75370
H	1.45301	-5.71641	-1.07197
H	-0.25847	-5.31032	-1.36333
H	0.39932	-5.27741	0.30073
C	4.87254	-1.80278	-0.85904
H	5.47769	-2.68834	-0.59846
H	5.52844	-0.91801	-0.79224
H	4.53867	-1.90778	-1.90147
C	4.21228	-1.89914	2.08933
H	3.47092	-1.85733	2.90233
H	4.98248	-1.13492	2.28728
H	4.70417	-2.88681	2.12872
C	3.01954	1.06370	-0.02117
C	3.30845	1.92089	1.09121
C	3.89806	3.18201	0.87367
H	4.10134	3.82731	1.73618
C	4.23355	3.62421	-0.40917
H	4.69948	4.60431	-0.55821
C	3.97052	2.78640	-1.49768
H	4.23127	3.12107	-2.50831
C	3.37467	1.51952	-1.33561
C	2.97544	1.52958	2.52781
H	2.63348	0.48219	2.49808
C	4.19967	1.61012	3.46624
H	5.04813	1.02043	3.08154
H	3.94378	1.22704	4.47002
H	4.54894	2.65032	3.59171
C	1.81875	2.39198	3.07920
H	2.10838	3.45675	3.13313

H	1.53998	2.06851	4.09841
H	0.93000	2.31833	2.43086
C	3.09333	0.70488	-2.59727
H	2.75052	-0.28909	-2.26491
C	4.34141	0.52599	-3.49127
H	4.65795	1.48492	-3.93877
H	4.11838	-0.16176	-4.32639
H	5.19925	0.11968	-2.93292
C	1.95607	1.34462	-3.42315
H	1.03482	1.45636	-2.83063
H	1.72371	0.72778	-4.30955
H	2.24477	2.34979	-3.77935
C	-1.46173	1.69586	-0.12638
H	-2.55454	2.44391	2.20287
C	-2.95531	1.71391	-0.23162
C	-3.88010	0.70972	0.07575
H	-3.54764	-0.23642	0.51175
C	-5.23847	0.93532	-0.22313
H	-5.97839	0.16279	0.00799
C	-5.64717	2.13363	-0.83501
H	-6.70664	2.28883	-1.06512
C	-4.70936	3.12589	-1.18868
H	-5.03594	4.03539	-1.70422
C	-3.35946	2.90774	-0.89354
C	-2.15069	3.69951	-1.20619
C	-1.99695	4.96178	-1.78925
H	-2.86582	5.54594	-2.11096
C	-0.69429	5.47910	-1.94601
H	-0.55932	6.46605	-2.40142
C	0.43063	4.75321	-1.51581
H	1.43381	5.17289	-1.63695
C	0.28165	3.48485	-0.92189
H	1.15416	2.92427	-0.57247
C	-1.00790	2.95992	-0.78816
C	-1.07089	0.12249	2.25721
C	-2.63280	1.86160	3.14242
C	-4.11733	1.68030	3.49536
H	-4.65669	1.16815	2.68395
H	-4.59177	2.66166	3.66246
H	-4.22051	1.08160	4.41644
C	-1.89029	2.63170	4.25295
H	-0.82918	2.77889	3.99840
H	-1.94968	2.08499	5.20953
H	-2.35264	3.62353	4.39490
C	0.54543	-1.27620	3.49541
H	1.56763	-0.85087	3.53635
C	0.68251	-2.80225	3.36259
H	1.29373	-3.19139	4.19544
H	1.17279	-3.08170	2.41597
H	-0.30592	-3.28785	3.40838
C	-0.17003	-0.89707	4.79830
H	-0.21367	0.19239	4.94913
H	0.38818	-1.33719	5.64238
H	-1.19942	-1.28822	4.82389

B
SCF (BP86) Energy = -2229.16074141
Enthalpy 0K = -2228.124338
Enthalpy 298K = -2228.058579
Free Energy 298K = -2228.220021
Lowest Frequency = 15.6907 cm^{-1}
Second Frequency = 23.7052 cm^{-1}
SCF (BP86-D3BJ) Energy = -2229.51590413
SCF (THF) Energy = -2229.16899716
SCF (BS2) Energy = -3041.3827806

Al	0.70302	-0.66739	0.62583
Si	0.70458	-3.45675	0.11725
Si	3.36001	-1.71314	0.52923
O	1.90620	-2.54634	0.98866
N	-0.40116	-2.09704	-0.00837
N	2.47641	-0.30404	-0.03396
N	-0.48176	1.11793	0.39720
N	-0.29764	2.50701	2.38949
N	0.25171	0.16379	2.31722
C	-1.81618	-2.15050	-0.25872
C	-2.74990	-2.29129	0.82182
C	-4.13176	-2.21387	0.55845
H	-4.83471	-2.30435	1.39476
C	-4.62771	-2.04438	-0.73662
H	-5.70584	-1.97650	-0.91699
C	-3.72348	-2.00192	-1.80153
H	-4.10458	-1.92659	-2.82583
C	-2.33184	-2.06833	-1.59522
C	-2.32955	-2.62629	2.25500
H	-1.22730	-2.64597	2.27991
C	-2.85959	-4.02367	2.66110
H	-3.96138	-4.02172	2.73819
H	-2.45935	-4.32088	3.64721
H	-2.58273	-4.79933	1.92945
C	-2.79559	-1.58126	3.28784
H	-2.31335	-0.61115	3.09947
H	-2.52561	-1.90132	4.31045
H	-3.89098	-1.44232	3.26298
C	-1.43040	-2.17693	-2.82496
H	-0.39405	-1.99243	-2.49122
C	-1.51504	-3.61502	-3.39626
H	-1.32534	-4.37788	-2.62339
H	-0.78842	-3.76216	-4.21542
H	-2.52509	-3.80750	-3.80037
C	-1.77010	-1.17076	-3.94355
H	-2.76608	-1.36738	-4.37866
H	-1.03915	-1.25441	-4.76639
H	-1.76100	-0.13234	-3.58019
C	1.43900	-4.15187	-1.48696
H	1.70039	-3.36239	-2.20874
H	0.70998	-4.82211	-1.97171
H	2.34138	-4.74895	-1.27124
C	0.21747	-4.93895	1.18323
H	1.05377	-5.65965	1.19605
H	-0.66159	-5.45755	0.76509
H	-0.00956	-4.65080	2.22022
C	4.44079	-2.67123	-0.69736
H	4.70456	-3.64987	-0.25907
H	5.38604	-2.12199	-0.85152
H	3.97836	-2.83797	-1.67944
C	4.38145	-1.61731	2.12316
H	3.84199	-1.12322	2.94546
H	5.32883	-1.07768	1.96153
H	4.63161	-2.64383	2.44510
C	2.99044	0.87440	-0.67031
C	3.46760	1.98646	0.09829
C	3.79149	3.19028	-0.55851
H	4.13430	4.04340	0.03843
C	3.71434	3.31285	-1.95039
H	3.95855	4.26173	-2.43992
C	3.37726	2.18673	-2.70961
H	3.39715	2.24961	-3.80428
C	3.03512	0.96151	-2.10162
C	3.80148	1.85454	1.58586
H	3.37747	0.89649	1.92851
C	5.34192	1.79109	1.74691
H	5.79403	1.02320	1.09612
H	5.61895	1.56736	2.79263
H	5.80147	2.75849	1.47613
C	3.22960	2.96315	2.49045

```
H   3.55762  3.96781  2.16839
H   3.58866  2.82316  3.52629
H   2.12890  2.94458  2.51074
C   2.86562 -0.27149 -2.99418
H   2.52107 -1.09176 -2.34097
C   4.23726 -0.66176 -3.59932
H   4.58621  0.11471 -4.30325
H   4.16616 -1.61240 -4.15844
H   5.01167 -0.77434 -2.82388
C   1.83726 -0.11097 -4.13096
H   0.82624  0.09275 -3.74871
H   1.79045 -1.03463 -4.73510
H   2.11342  0.70986 -4.81748
C  -1.54858  1.54043 -0.25241
H  -1.37011  3.51469  0.84510
C  -2.95077  1.72924  0.21995
C  -3.53576  1.53252  1.47733
H  -2.92283  1.26679  2.34138
C  -4.92544  1.70810  1.61458
H  -5.39283  1.56671  2.59400
C  -5.71542  2.06721  0.50856
H  -6.79503  2.20008  0.63436
C  -5.13575  2.25461 -0.76152
H  -5.76002  2.52328 -1.62015
C  -3.75488  2.08540 -0.90325
C  -2.87717  2.23378 -2.07972
C  -3.12965  2.70666 -3.37118
H  -4.14297  2.97609 -3.68696
C  -2.04291  2.86442 -4.25638
H  -2.22443  3.24083 -5.26862
C  -0.72825  2.57456 -3.84917
H   0.10346  2.73212 -4.54229
C  -0.46814  2.08966 -2.55282
H   0.55571  1.89326 -2.21859
C  -1.55117  1.89363 -1.68889
C  -0.25953  1.33862  1.81938
C  -0.84042  3.72334  1.80251
C  -1.86719  4.34732  2.77153
H  -2.70728  3.66216  2.96758
H  -2.27683  5.28204  2.35169
H  -1.38099  4.58009  3.73423
C   0.28378  4.73024  1.48906
H   1.00759  4.30809  0.77343
H   0.82754  5.00556  2.40861
H  -0.14330  5.64999  1.05191
C   0.80852  0.07916  3.68703
H   1.77816  0.62409  3.69668
C   1.07271 -1.39406  4.04035
H   1.61627 -1.46058  4.99842
H   1.66089 -1.91380  3.26871
H   0.11717 -1.93469  4.15606
C  -0.08033  0.70985  4.77799
H  -0.24666  1.77904  4.58719
H   0.41147  0.58803  5.75899
H  -1.05802  0.20077  4.82679
```

TS(B-C)

TS_CH_ME5_rotR

SCF (BP86) Energy = -2229.15063010

Enthalpy 0K = -2228.119189

Enthalpy 298K = -2228.053536

Free Energy 298K = -2228.214404

Lowest Frequency = -419.2452 cm^{-1}

Second Frequency = 20.7816 cm^{-1}

SCF (BP86-D3BJ) Energy = -2229.50887473

SCF (THF) Energy = -2229.15788941

SCF (BS2) Energy = -3041.37484718

```
Al  0.39508 -0.85400  0.37407
Si -0.04248 -3.43743 -1.09505
Si  2.84477 -2.49623 -0.33451
O   1.41056 -3.40988 -0.19071
N  -0.83927 -1.95304 -0.53504
N   2.19054 -0.85109 -0.24313
N  -0.33410  0.94990  0.87523
N  -0.11267  1.52772  3.17750
N   0.21502 -0.71033  2.31621
C  -2.27376 -1.82886 -0.65765
C  -3.15315 -2.27912  0.38595
C  -4.54898 -2.17826  0.21522
H  -5.20479 -2.53021  1.02019
C  -5.11643 -1.66030 -0.95184
H  -6.20424 -1.60456 -1.06868
C  -4.26581 -1.22169 -1.96884
H  -4.69729 -0.81086 -2.88876
C  -2.86355 -1.29067 -1.84956
C  -2.66303 -2.87140  1.70837
H  -1.56537 -2.95329  1.64661
C  -3.24993 -4.27639  1.98320
H  -4.33752 -4.22969  2.17062
H  -2.78424 -4.71683  2.88308
H  -3.08784 -4.96695  1.14147
C  -3.00661 -1.94121  2.89367
H  -2.51044 -0.96284  2.79353
H  -2.67649 -2.38811  3.84867
H  -4.09693 -1.77710  2.96559
C  -2.04293 -0.76587 -3.02837
H  -0.97747 -0.90529 -2.77758
C  -2.34653 -1.54194 -4.33147
H  -2.24152 -2.63100 -4.19875
H  -1.66406 -1.22694 -5.14071
H  -3.37819 -1.35190 -4.67767
C  -2.29747  0.74141 -3.25514
H  -3.36270  0.93551 -3.47168
H  -1.70947  1.11525 -4.11070
H  -2.02962  1.33674 -2.36821
C   0.36706 -3.52777 -2.94706
H   0.79814 -2.59449 -3.34229
H  -0.54110 -3.75278 -3.53050
H   1.08493 -4.34743 -3.12446
C  -0.94312 -5.03712 -0.64893
H  -0.44923 -5.88682 -1.15088
H  -1.99435 -5.01045 -0.98059
H  -0.92142 -5.21848  0.43636
C   3.81331 -2.88337 -1.91592
H   4.20200 -3.91489 -1.85891
H   4.67942 -2.20511 -2.00497
H   3.20824 -2.79035 -2.82930
C   3.92623 -3.10669  1.09942
H   3.51642 -2.85283  2.08900
H   4.94951 -2.70263  1.03643
H   3.99698 -4.20670  1.03202
C   3.02943  0.28231 -0.54843
C   3.83669  0.90962  0.45878
C   4.62781  2.02779  0.12279
H   5.23417  2.49923  0.90502
C   4.67365  2.53507 -1.17910
H   5.30473  3.39750 -1.42050
C   3.91307  1.90716 -2.17036
H   3.94895  2.28817 -3.19747
C   3.09708  0.79441 -1.88738
C   3.92427  0.39732  1.89653
H   3.26627 -0.48482  1.96569
C   5.36704 -0.03894  2.24731
H   5.78041 -0.73741  1.50174
H   5.39504 -0.53292  3.23463
```

```
H   6.04543  0.83164  2.29342
C   3.43699  1.44109  2.92559
H   4.04605  2.36187  2.87671
H   3.52725  1.04235  3.95218
H   2.38126  1.71434  2.77022
C   2.30059  0.19216 -3.04426
H   1.80609 -0.71473 -2.65566
C   3.20047 -0.20909 -4.23596
H   3.64637  0.67803 -4.71999
H   2.60704 -0.73442 -5.00559
H   4.02604 -0.87025 -3.92903
C   1.20325  1.16231 -3.53149
H   0.50883  1.43529 -2.72159
H   0.61884  0.70657 -4.35008
H   1.64550  2.09866 -3.91586
C  -0.95705  2.11809  0.49882
H  -0.90772  2.79024  1.77703
C  -2.43415  2.29477  0.25967
C  -3.51469  1.49879  0.65146
H  -3.35518  0.56149  1.19311
C  -4.81401  1.90395  0.29067
H  -5.66849  1.28428  0.57782
C  -5.01812  3.08052 -0.45238
H  -6.03607  3.37937 -0.72386
C  -3.92839  3.86405 -0.87890
H  -4.09549  4.75793 -1.48945
C  -2.63187  3.46312 -0.53046
C  -1.31347  4.01653 -0.89089
C  -0.95349  5.14574 -1.63723
H  -1.71551  5.80553 -2.06573
C   0.41492  5.41861 -1.83208
H   0.71020  6.29610 -2.41724
C   1.40620  4.58128 -1.28837
H   2.46411  4.80123 -1.45901
C   1.05428  3.44986 -0.52743
H   1.82429  2.79792 -0.10267
C  -0.30493  3.17373 -0.34247
C  -0.14764  0.59064  2.24760
C  -0.75768  2.73811  3.04642
C  -2.20725  2.81491  3.54945
H  -2.79274  1.93880  3.23447
H  -2.70950  3.72846  3.19227
H  -2.19317  2.83996  4.65352
C   0.08661  3.96331  3.40972
H   1.06031  3.93402  2.89773
H   0.27098  3.97462  4.49842
H  -0.43546  4.89657  3.14330
C   0.81677 -1.32493  3.51684
H   1.87591 -0.99323  3.57878
C   0.80670 -2.85419  3.35142
H   1.42781 -3.32468  4.13274
H   1.19095 -3.16664  2.36693
H  -0.21938 -3.24715  3.45332
C   0.13771 -0.93815  4.84470
H   0.20026  0.14392  5.03264
H   0.63447 -1.47029  5.67465
H  -0.92569 -1.23066  4.83964
```

C

SCF (BP86) Energy = -2229.18124553
Enthalpy 0K = -2228.145385
Enthalpy 298K = -2228.078975
Free Energy 298K = -2228.242539
Lowest Frequency = 17.7804 cm-1
Second Frequency = 24.4745 cm-1
SCF (BP86-D3BJ) Energy = -2229.53917773
SCF (THF) Energy = -2229.19031652
SCF (BS2) Energy = -3041.40643164

```
Al  0.08737 -0.82211  0.21548
Si -1.11490 -3.00407 -1.51869
Si  1.94365 -2.98326 -0.92152
O   0.33442 -3.53225 -0.78426
N  -1.44386 -1.47355 -0.67054
N   1.77198 -1.25603 -0.54310
N   0.01440  0.95503  1.10948
N   0.74350  1.04032  3.41544
N   0.19096 -0.99041  2.20930
C  -2.81217 -1.00588 -0.59970
C  -3.66127 -1.35721  0.50488
C  -4.98980 -0.88762  0.54384
H  -5.62111 -1.16118  1.39745
C  -5.52882 -0.10954 -0.48387
H  -6.56872  0.23244 -0.44083
C  -4.71718  0.20701 -1.57523
H  -5.12985  0.80731 -2.39389
C  -3.37662 -0.21941 -1.65821
C  -3.21348 -2.25154  1.66186
H  -2.19280 -2.59853  1.43221
C  -4.12562 -3.48935  1.83575
H  -5.13521 -3.20159  2.17916
H  -3.70516 -4.17207  2.59602
H  -4.24272 -4.05255  0.89726
C  -3.16462 -1.46771  2.99101
H  -2.43488 -0.64584  2.93409
H  -2.86704 -2.12693  3.82684
H  -4.15385 -1.04296  3.23904
C  -2.60216  0.18562 -2.91303
H  -1.58447 -0.23065 -2.82359
C  -3.26124 -0.38747 -4.19079
H  -3.44716 -1.47141 -4.11301
H  -2.62233 -0.20865 -5.07360
H  -4.23527  0.09612 -4.38508
C  -2.48902  1.72081 -3.03879
H  -3.48700  2.18890 -3.09889
H  -1.93830  1.99723 -3.95481
H  -1.96839  2.16348 -2.17627
C  -0.83964 -2.90256 -3.39504
H  -0.17894 -2.07278 -3.69136
H  -1.79566 -2.77958 -3.92873
H  -0.38593 -3.84807 -3.74015
C  -2.41221 -4.34451 -1.21541
H  -2.22806 -5.19249 -1.89707
H  -3.43134 -3.96932 -1.40552
H  -2.36308 -4.71874 -0.18142
C   2.70217 -3.31487 -2.62544
H   2.85773 -4.39981 -2.75450
H   3.68579 -2.82094 -2.70429
H   2.07108 -2.95448 -3.45077
C   2.88373 -4.09377  0.29761
H   2.62399 -3.90334  1.35015
H   3.97494 -3.99326  0.18771
H   2.61904 -5.14098  0.06744
C   2.88471 -0.38110 -0.84127
C   3.98271 -0.22764  0.06997
C   5.06383  0.61034 -0.27348
H   5.89493  0.71592  0.43368
C   5.11020  1.29253 -1.49202
H   5.96572  1.92861 -1.74441
C   4.04708  1.13884 -2.38615
H   4.07076  1.66924 -3.34484
C   2.94050  0.31962 -2.09162
C   4.06664 -0.95336  1.41332
H   3.17949 -1.60440  1.48393
C   5.33306 -1.83739  1.50732
H   5.44349 -2.49730  0.63168
```

```
H   5.30202 -2.46856  2.41312
H   6.24636 -1.21910  1.56819
C   4.04046  0.02803  2.60714
H   4.90322  0.71735  2.56922
H   4.10264 -0.52384  3.56303
H   3.12026  0.63291  2.63168
C   1.83054  0.23551 -3.13834
H   1.11148 -0.52566 -2.78819
C   2.35569 -0.19415 -4.52789
H   3.00523  0.58171 -4.97077
H   1.51325 -0.34792 -5.22579
H   2.93850 -1.12784 -4.48114
C   1.08388  1.58132 -3.26088
H   0.63105  1.88860 -2.30503
H   0.28345  1.51371 -4.01738
H   1.77248  2.38763 -3.56914
C  -0.16764  2.39878  1.02354
H  -0.07606  2.79833  2.05595
C  -1.51432  2.92660  0.51913
C  -2.80848  2.48343  0.79991
H  -2.98170  1.55497  1.35432
C  -3.90133  3.22879  0.31546
H  -4.91889  2.87802  0.51151
C  -3.69385  4.40271 -0.43133
H  -4.55528  4.97355 -0.79435
C  -2.39315  4.83964 -0.73447
H  -2.23748  5.74349 -1.33353
C  -1.30088  4.08953 -0.26768
C   0.14700  4.27208 -0.46010
C   0.86369  5.24981 -1.16958
H   0.34264  6.06122 -1.68949
C   2.26560  5.16434 -1.20789
H   2.83891  5.91538 -1.76209
C   2.93996  4.11986 -0.54907
H   4.03048  4.05229 -0.60544
C   2.22648  3.14585  0.17613
H   2.75869  2.33050  0.67592
C   0.83346  3.22857  0.21484
C   0.25594  0.34985  2.31245
C   0.07192  1.72008  4.28403
C  -1.43431  1.84268  4.33901
H  -1.92536  1.38134  3.47142
H  -1.73134  2.90448  4.39448
H  -1.81210  1.36019  5.25896
C   0.84438  2.42726  5.37314
H   1.92447  2.26813  5.24962
H   0.52963  2.05855  6.36656
H   0.62905  3.51064  5.35767
C   0.72809 -1.97590  3.17095
H   1.83122 -2.00698  3.04559
C   0.17077 -3.36418  2.81017
H   0.72018 -4.14556  3.36256
H   0.25628 -3.57656  1.73232
H  -0.89368 -3.43812  3.08999
C   0.43910 -1.67951  4.65625
H   0.99992 -0.81022  5.02689
H   0.73499 -2.55382  5.26123
H  -0.63950 -1.50847  4.81759
```

4

SCF (BP86) Energy = -2229.19028815
Enthalpy 0K = -2228.154288
Enthalpy 298K = -2228.087909
Free Energy 298K = -2228.251427
Lowest Frequency = 22.3132 cm^{-1}
Second Frequency = 27.7277 cm^{-1}
SCF (BP86-D3BJ) Energy = -2229.54650973
SCF (THF) Energy = -2229.19827457
SCF (BS2) Energy = -3041.41593642

```
Al  0.85885  0.06675  0.40514
Si  3.66854 -0.99763 -0.06998
Si  3.23673  2.00317  0.66273
O   3.70508  0.38618  0.92997
N   1.93564 -1.38858 -0.11912
N   1.55855  1.80731  0.11975
N  -1.06013 -0.08752  0.05749
N  -2.58676  0.30099  1.87416
N  -0.18268 -0.05467  2.10766
C   1.49903 -2.73433 -0.41529
C   1.35746 -3.71387  0.62657
C   0.92919 -5.01815  0.30735
H   0.81897 -5.74938  1.11645
C   0.65494 -5.40692 -1.00613
H   0.33339 -6.42928 -1.23224
C   0.81058 -4.46534 -2.02598
H   0.60878 -4.75985 -3.06207
C   1.22210 -3.14250 -1.76399
C   1.64407 -3.42655  2.10175
H   2.05781 -2.40608  2.16583
C   2.66913 -4.41357  2.70915
H   2.25358 -5.43450  2.77893
H   2.93875 -4.10191  3.73409
H   3.59390 -4.47122  2.11479
C   0.34303 -3.46794  2.93321
H  -0.35599 -2.68614  2.59851
H   0.55530 -3.30196  4.00518
H  -0.15563 -4.44929  2.84177
C   1.36300 -2.21368 -2.97104
H   1.62450 -1.21152 -2.58883
C   2.49272 -2.68843 -3.91602
H   3.44542 -2.82965 -3.38144
H   2.65844 -1.95830 -4.72801
H   2.23279 -3.65436 -4.38471
C   0.04432 -2.11026 -3.77113
H  -0.23463 -3.08475 -4.20897
H   0.14589 -1.39411 -4.60404
H  -0.79848 -1.78746 -3.13870
C   4.47664 -0.58395 -1.73906
H   3.85356  0.06289 -2.37657
H   4.69325 -1.50604 -2.30334
H   5.43933 -0.07410 -1.56032
C   4.75642 -2.30604  0.75194
H   5.81682 -2.03867  0.60356
H   4.59281 -3.30166  0.30706
H   4.56663 -2.36914  1.83407
C   4.34933  2.89326 -0.58603
H   5.35417  3.02933 -0.15026
H   3.94309  3.89433 -0.81144
H   4.45378  2.34340 -1.53280
C   3.55196  2.83969  2.33652
H   2.87750  2.48939  3.13278
H   3.46520  3.93584  2.26877
H   4.58718  2.60465  2.64090
C   0.86049  2.93263 -0.46050
C   0.18326  3.89933  0.35592
C  -0.47445  4.98952 -0.25065
H  -0.98635  5.71782  0.38895
C  -0.48957  5.16585 -1.63671
H  -1.00672  6.01995 -2.08685
C   0.17636  4.23276 -2.43635
H   0.17740  4.36237 -3.52477
C   0.85635  3.13034 -1.88272
C   0.13773  3.82367  1.88197
H   0.72139  2.93616  2.17897
C   0.77861  5.07190  2.53378
```

H	1.78638	5.27471	2.13693
H	0.85899	4.94268	3.62765
H	0.16664	5.97339	2.35228
C	-1.30694	3.65523	2.40700
H	-1.92758	4.52861	2.13770
H	-1.31158	3.58056	3.50996
H	-1.80023	2.75770	2.00097
C	1.56150	2.18445	-2.85451
H	2.10080	1.43776	-2.24607
C	2.58535	2.91690	-3.75299
H	2.08336	3.61204	-4.44911
H	3.14818	2.19097	-4.36675
H	3.30853	3.50183	-3.16364
C	0.54404	1.43759	-3.74307
H	-0.16925	0.85209	-3.14026
H	1.05955	0.74506	-4.43105
H	-0.04367	2.14422	-4.35536
C	-2.08584	-0.19669	-0.97651
H	-1.52536	-0.16760	-1.93251
C	-3.15707	0.89966	-1.05371
C	-2.99421	2.28679	-1.06259
H	-2.00484	2.73930	-0.94375
C	-4.13458	3.09783	-1.21914
H	-4.02158	4.18685	-1.22180
C	-5.41057	2.52544	-1.37844
H	-6.28431	3.17354	-1.50664
C	-5.57447	1.12984	-1.37881
H	-6.56874	0.68860	-1.51135
C	-4.44065	0.31662	-1.21144
C	-4.30463	-1.14956	-1.16028
C	-5.26824	-2.16997	-1.24434
H	-6.32864	-1.93155	-1.38364
C	-4.84789	-3.50882	-1.15088
H	-5.58790	-4.31397	-1.21523
C	-3.48645	-3.82536	-0.98199
H	-3.17420	-4.87257	-0.91525
C	-2.51593	-2.80660	-0.90391
H	-1.45741	-3.05751	-0.77576
C	-2.93374	-1.47519	-0.98808
C	-1.33044	-0.01186	1.39356
C	-3.52027	-0.50252	2.25813
C	-3.41466	-2.00670	2.33254
H	-2.42820	-2.37374	2.01960
H	-4.18165	-2.47111	1.68797
H	-3.61491	-2.33959	3.36719
C	-4.83496	0.10441	2.68577
H	-4.79504	1.20048	2.62578
H	-5.09063	-0.20590	3.71505
H	-5.64441	-0.26407	2.03065
C	0.00648	0.36523	3.51043
H	-0.06983	1.47128	3.55212
C	1.43209	-0.02622	3.93606
H	1.68736	0.45132	4.89733
H	2.18087	0.28004	3.18754
H	1.51372	-1.11811	4.06606
C	-1.01084	-0.20381	4.51965
H	-2.02018	0.20488	4.37060
H	-0.69151	0.06291	5.54198
H	-1.05492	-1.30414	4.45679
